# Synthesis, Structure–Activity Relationships, Radiofluorination, and Biological Evaluation of [^18^F]RM365, a Novel Radioligand for Imaging the Human Cannabinoid Receptor Type 2 (CB2R) in the Brain with PET

**DOI:** 10.1021/acs.jmedchem.3c01035

**Published:** 2023-10-10

**Authors:** Rodrigo Teodoro, Daniel Gündel, Winnie Deuther-Conrad, Aleksandr Kazimir, Magali Toussaint, Barbara Wenzel, Guy Bormans, Evamarie Hey-Hawkins, Klaus Kopka, Peter Brust, Rareş-Petru Moldovan

**Affiliations:** † Institute of Radiopharmaceutical Cancer Research, Department of Neuroradiopharmaceuticals, Research Site Leipzig, Helmholtz-Zentrum Dresden-Rossendorf (HZDR), 04318 Leipzig, Germany; ‡ Faculty of Chemistry and Mineralogy, Institute of Inorganic Chemistry, Universität Leipzig, Johannisallee 29, 04103 Leipzig, Germany; § Radiopharmaceutical Research, Department of Pharmaceutical and Pharmacological Sciences, KU Leuven, BE-3000 Leuven, Belgium; ∥ Faculty of Chemistry and Food Chemistry, School of Science, TU Dresden, 01069 Dresden, Germany; ⊥ The Lübeck Institute of Experimental Dermatology, University Medical Center Schleswig-Holstein, 23562 Lübeck, Germany

## Abstract

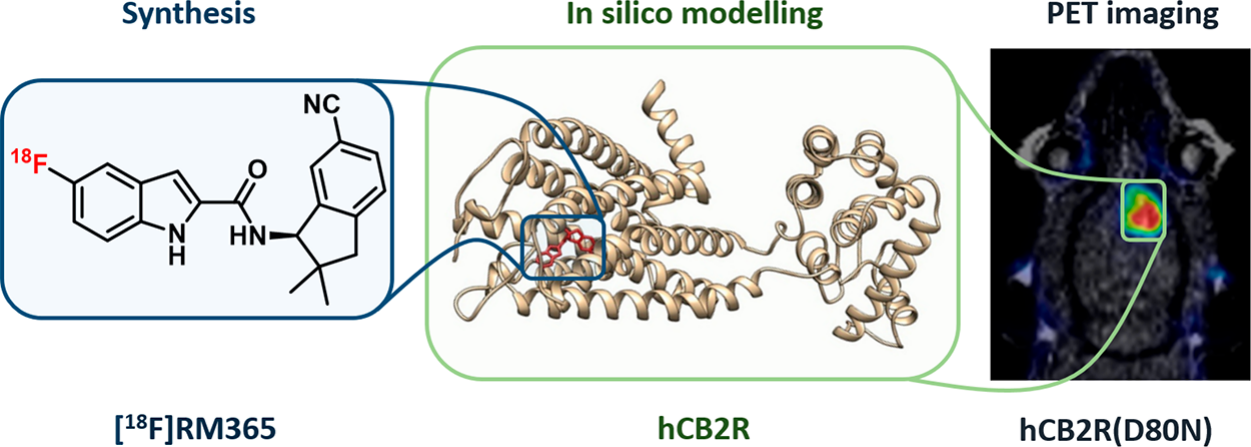

The development of cannabinoid receptor type 2 (CB2R) PET radioligands has been intensively explored due to the pronounced CB2R upregulation under various pathological conditions. Herein, we report on the synthesis of a series of CB2R affine fluorinated indole-2-carboxamide ligands. Compound RM365 was selected for PET radiotracer development due to its high CB2R affinity (*K*_i_ = 2.1 nM) and selectivity over CB1R (factor > 300). Preliminary *in vitro* evaluation of [^18^F]RM365 indicated species differences in the binding to CB2R (*K*_D_ of 2.32 nM for the hCB2R vs *K*_D_ > 10,000 nM for the rCB2R). Metabolism studies in mice revealed a high in vivo stability of [^18^F]RM365. PET imaging in a rat model of local hCB2R(D80N) overexpression in the brain demonstrates the ability of [^18^F]RM365 to reach and selectively label the hCB2R(D80N) with a high signal-to-background ratio. Thus, [^18^F]RM365 is a very promising PET radioligand for the imaging of upregulated hCB2R expression under pathological conditions.

## Introduction

Cannabinoid receptors (CBRs) belong to the G-protein-coupled receptors (GPCRs) and are activated by endocannabinoids, phytocannabinoids from the cannabis plant, and synthetic CBR ligands.^1−4^ CBRs are primarily located in the central and peripheral nervous systems and are involved in a variety of physiological processes, including pain, appetite, memory, and immune function.^3,5,6^ There are two main types of cannabinoid receptors: CB1R and CB2R. CB1R is a GPCR coupled to pertussis toxin-sensitive inhibitory G (Gi/o) protein, primarily found in the brain and spinal cord, where they are involved in the regulation of neurotransmitters, such as dopamine and glutamate.^7−10^ CB2R is a GPCR predominantly coupled to the inhibitory guanine nucleotide binding protein (Gi protein) receptors, primarily found in the immune system and are involved in the regulation of inflammatory processes.^11−13^ The most well-known cannabinoid is (−)-Δ^9^-trans-tetrahydrocannabinol (THC), which is responsible for the psychoactive effects of cannabis.^1,14^ Up to date, more than 200 cannabinoids from the cannabis plant were reported, including cannabidiol (CBD), which does not have psychoactive effects but has been shown to have therapeutic potential for a variety of conditions, such as anxiety, pain, and inflammation.^15,16^

While psychoactive effects are transmitted via the activation of CB1R, a selective targeting of CB2R was proposed as a safe and promising therapeutic approach for a wide range of medical applications, including cancer,^17^ neurological disorders, and metabolic disorders.^18^ When activated, CB2R leads to a decrease in cAMP production by inhibiting the activity of adenylyl cyclase, resulting in a decrease in the downstream effects of cAMP, such as the activation of protein kinase A (PKA) and the inhibition of the formation of cAMP-dependent protein kinases.^19^ The impact of the intracellular level of the secondary messenger cAMP on a great variety of cellular processes,^20^ and the location of CB2R in the immune system of both the brain and the periphery, as well as in microglia and endothelial cells, makes the receptor an interesting pharmacological target for the treatment of pain syndromes, neuroinflammation, and neurodegenerative processes.^21^ Accordingly, neuroimaging of CB2R by positron emission tomography (PET) has the potential to support the development of CB2R-directed drugs (e.g., improvement of clinical trials by stratification of patients) and research of CB2R-associated neurological diseases.^22−24^ While early studies failed to detect CB2R in brain tissue,^25^ the presence and localization of CB2R in the brain were demonstrated by, e.g., RT-PCR and immunohistochemical analysis.^26^ Given the low level of expression of CB2R along with the high level of CB1R under physiological conditions, for the neuroimaging of CB2R with PET, a radioligand binding to CB2R with high affinity with *K*_D_ in low nanomolar range and high selectivity versus CB1R is needed.^22^

**Figure 1:**
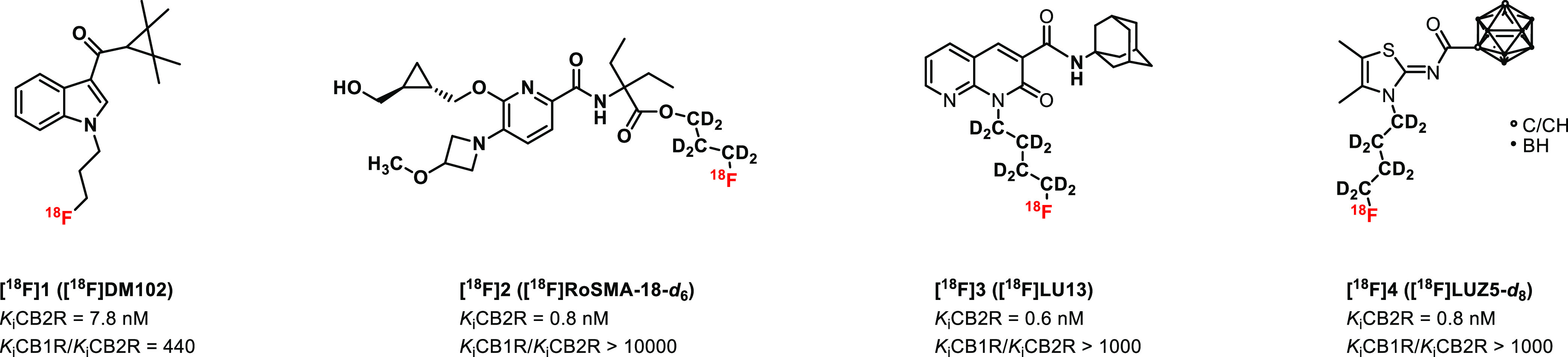
Most recently developed ^18^F-labeled CB2R ligands.

A number of PET radioligands were developed for CB2R to date and excellently summed-up in several recent reviews,^22,23,27^ with the most recent CB2R radioligands depicted in Figure 1. Compound [^18^F]DM102 was reported by Modemann and co-workers and proved by autoradiography with spleen tissue to bind to CB2R.^28^ The most recently ^18^F-labeled CB2R ligand reported by the group of Ametamey is [^18^F]RoSMA-18-*d*_6_,^29,30^ which besides the subnanomolar CB2R affinity and selectivity showed excellent imaging properties as demonstrated by *in vitro* autoradiography with rat spleen and spinal cord tissue from ALS patients as well as *in vivo* imaging in rat with PET. Gündel and co-workers reported [^18^F]LU13, a radioligand with high CB2R binding affinity and selectivity.^31^ Despite its inability to label CB2R in the spleen by autoradiography *in vitro*, compound [^18^F]LU13 is suitable to visualize intracranially overexpressed human CB2R(D80N) in a rat model.^32^ Recently, Ueberham and co-workers reported [^18^F]LUZ5-*d*_8_, the first carborane-based CB2R radioligand setting the stage toward theranostic application.^33^ Despite the large number of CB2R-targeting radioligands reported to date, little progress has been made in imaging this receptor in pathological conditions, which may be related either to the insufficient affinity of the radioligands or the too low expression of CB2R to be detected by PET.

**Figure 2:**
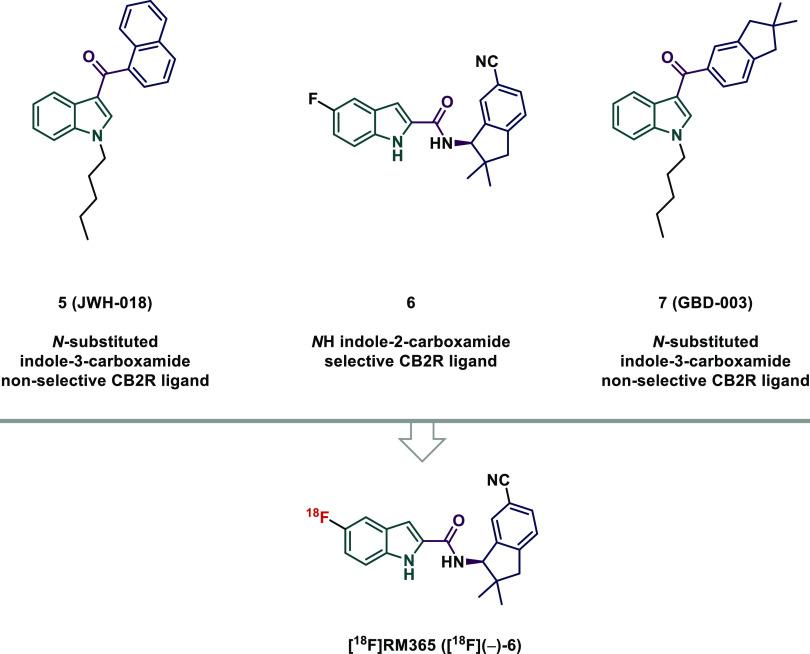
Rational design of a novel high affinity and selectivity ^18^F-labeled CB2R radioligand.

Besides quinolinones,^34−45^ naphthyridones,^46−48^ and thiazoles,^49−54^ indole^55−65^ is one of the most widely investigated scaffolds in the medicinal chemistry of CB2R-targeted small molecules. As the most broadly used substitution pattern, the *N*-alkyl-3-carbonyl indole plays a central role; however, the cannabinoid receptor ligands based on this scaffold most often suffer from low selectivity against CB1R as, e.g., JWH-018^65^ and the more recently reported compound GBD-003^59^ (Figure 2). On the other hand, a few structurally related ligands lacking the *N*-alkyl residue and bearing a 2-carboxamide instead possess high CB2R affinity and selectivity (e.g., **6**, Figure 2).^60^ In our continuous efforts to develop an ^18^F-labeled radioligand for the imaging of CB2R in the brain with PET, we carried out a medicinal chemistry study on the structure of compound **6** (Figure 2)^60^ due to its low nanomolar affinity for CB2R (CB2R *K*_i_ = 0.26 nM^60^). For efficient synthesis and easy access to a library of fluorinated derivatives and a precursor compound for radiofluorination based on the structure of **6**, an enantioselective synthesis was envisaged. The medicinal chemistry was carried out aiming at a fluorinated analogue of **6** with (i) high affinity toward CB2R, (ii) high selectivity toward CB1R, and (iii) an easily accessible position for aliphatic radiofluorination. The best candidate according to affinity and selectivity has been radiofluorinated and subsequently investigated by PET regarding the ability to image CB2R in the brain of a well-established and characterized rat model of local hCB2R overexpression.^32,66^

## Results and Discussion

### Chemistry

**Figure 3:**
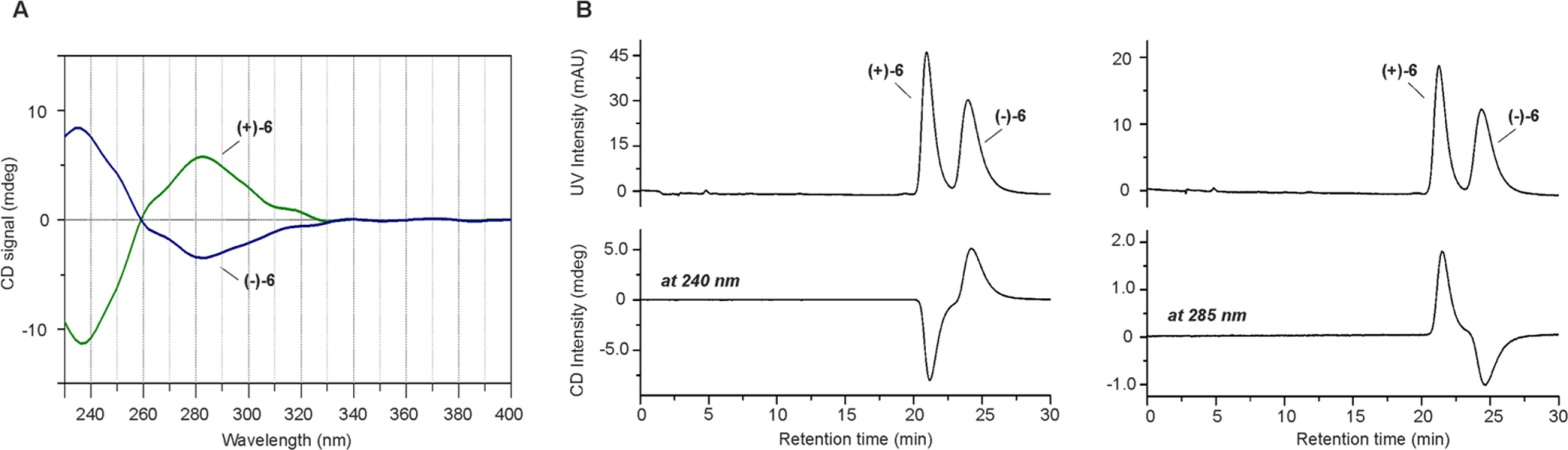
(A) CD spectra of (−)-**6** and (+)-**6** measured with chiral HPLC in stopped-flow mode and (B) chromatograms of the chiral separation of (±)-**6** measured at 240 and 285 nm.

**Scheme 1:**
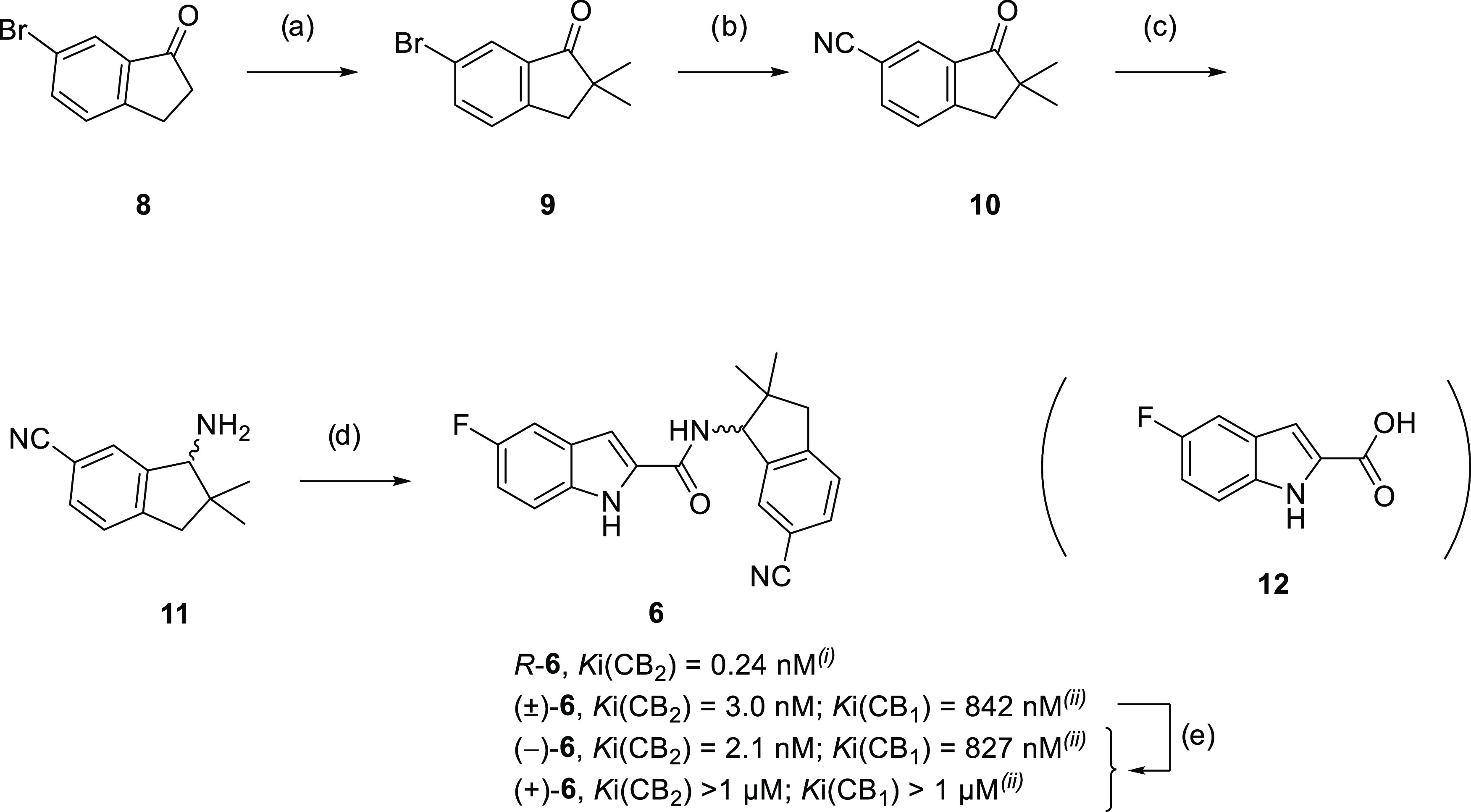
Synthesis of (±)-**6** and Chiral HPLC Resolution of (−)-**6** and (+)-**6** Reagents and conditions: (a) MeI, NaH (55% mineral oil suspension), toluene, 90 °C, 16 h, 72%; (b) CuCN, NMP, 175 °C, 4 h, 88%; (c) AA, EtOH, NaCNBH_4_, 80 °C, 21 h, 55%; (d) **12**, BOP, NEt_3_, CH_2_Cl_2_, 16 h, rt, 83%; (e) chiral HPLC separation. (i) Reported in ref (60). (ii) Determined in-house according to protocols previously reported by us.^53^ Abbreviations: MeI, methyl iodide; NaH, natrium hydride; CuCN, copper cyanide; NMP, *N*-methyl-2-pyrrolidon; AA, ammonium acetate; NaCNBH_4_, sodium cyanoborohydride; BOP, [(1*H*-benzo[*d*][1–3]triazol-1-yl)oxy]tris(dimethylamino)phosphonium hexafluorophosphate(V).

The synthesis of compound (±)-**6** was performed by a modified 4-step sequence starting from the commercially available 5-bromo-1-indanone (**8**), which is converted into the racemic amine **11** within three steps and 37% overall yield. In the first step, 5-bromo-1-indanone (**8**) was selectively double-methylated with MeI at position 2 in deprotonative (NaH) reaction conditions (Scheme 1). For the aromatic nitrilation, the Rosenmund–von Braun reaction^67−69^ was then employed after which the molecule was reductively aminated with ammonium acetate in the presence of sodium cyanoborhydride. In the last step, amine **11** was coupled with the commercially available acid **12** in the presence of the BOP reagent. Previously,^60^ the enantiomeric separation of amine **11** was performed by subcritical fluid chromatography using a chiralpack AD column, and only for (*R*)-**6** (optical rotation not mentioned), the CB2R affinity was reported.^60^ In the present study, we synthesized (±)-**6** and resolved the racemic mixture at the last step by chiral HPLC. This strategy allowed us to perform the organic synthesis in an easy-to-handle scale with final access to both enantiomers which could be investigated regarding their binding affinity toward CB1R and CB2R. The quantitative separation of the enantiomers was achieved by HPLC using a chiral column. A total of 3 mg of each enantiomer was separated, and the enantiopurity was determined by analytical chiral HPLC coupled with a chiral detector measuring the optical rotation (Figure S1 in the Supporting Information). Thus, we can assign the *R*-enantiomer to a minus rotation and the *S*-enantiomer to a plus rotation. Moreover, both enantiomers were investigated using another chiral detector, which is based on circular dichroism, a feature allowing optically active compounds to differently absorb right and left circularly polarized light. This difference is recorded and the value is referred to as ellipticity, often given in mdeg (millidegrees). As the ellipticity value depends on the absorbance wavelength and thus on the chromophores of a compound, the resulting CD spectrum is a typical characteristic for a chiral compound and the two enantiomers are usually giving mirror image spectra. The selection of the most suitable wavelength for CD monitoring of (+)-**6** and (−)-**6** was the first procedure to be performed, and the respective CD spectra were recorded with chiral HPLC in stopped-flow mode. As shown in Figure 3A, the enantiomers have two strong CD bands in the ranges of 230–250 and 270–290 nm. At ∼240 nm, a positive CD band is related to (−)-**6** (*R*-configuration) and a negative CD band to (+)-**6** (*S*-configuration). In contrast, at ∼285 nm, a positive CD band is related to (+)-**6** (*S*-configuration) and a negative CD band to (−)-**6** (*R*-configuration). As a consequence, the amplitudes in the respective chromatograms of both enantiomers recorded with the CD detector are dependent on the wavelength used, as can be seen in Figure 3B.

The investigation of the binding assays revealed that only (−)-**6** (RM365) possesses CB2R affinity with *K*_i_ (CB2R) = 2.1 nM (0.24 nM reported^60^) and (+)-**6** proved to be inactive. Compound (−)-**6** also possesses high CB2R selectivity with a *K*_i_ (CB1R) of 827 nM. To further investigate the potential of this scaffold to bind CB2R by developing fluorinated derivatives of (−)-**6**, an enantioselective synthesis was needed. Several highly efficient methods for enantioselective reductive amination were reported to date.^70^ In our efforts to synthesize enantiopure RM365 ((−)-**6**), the method developed by Ellman^71^ using sulfinamide **13** as chiral auxiliary was selected.

**Scheme 2:**
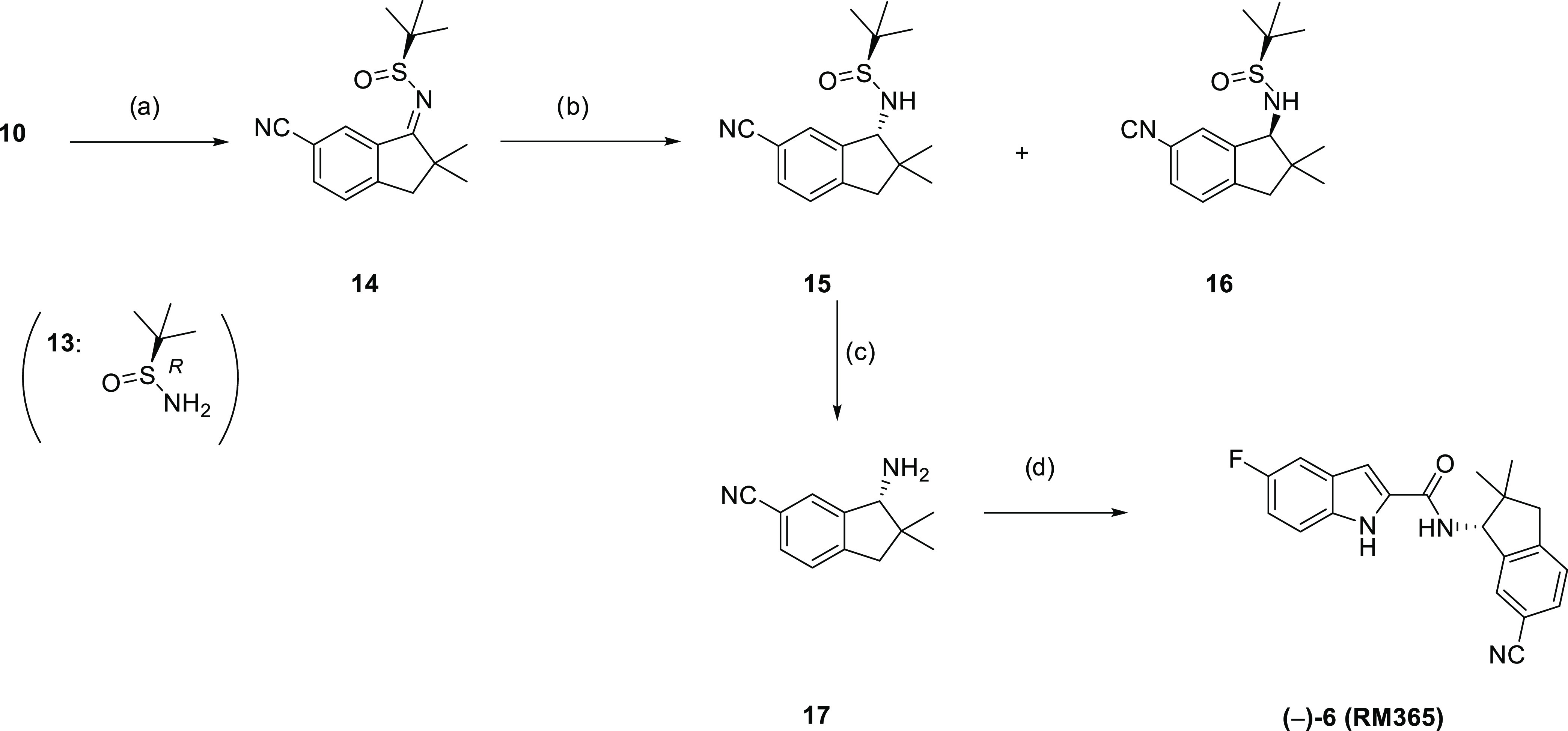
Enantioselective Synthesis of RM365 and Its Derivatives Reagents and conditions: (a) Ti(OEt)_4_, toluol, 70 °C, 2 h, 82%; (b) NaBH_4_, THF, −40 °C to rt, overnight, 32% yield for **15** and 28% yield for **16**; (c) TFA, DCM, quant; (d) **12**, BOP, NEt_3_, CH_2_Cl_2_, 16 h, rt, 82%. Abbreviations: Ti(OEt)_4_, titan(IV)-ethanolat; TFA, trifluoroacetic acid.

**Scheme 3:**
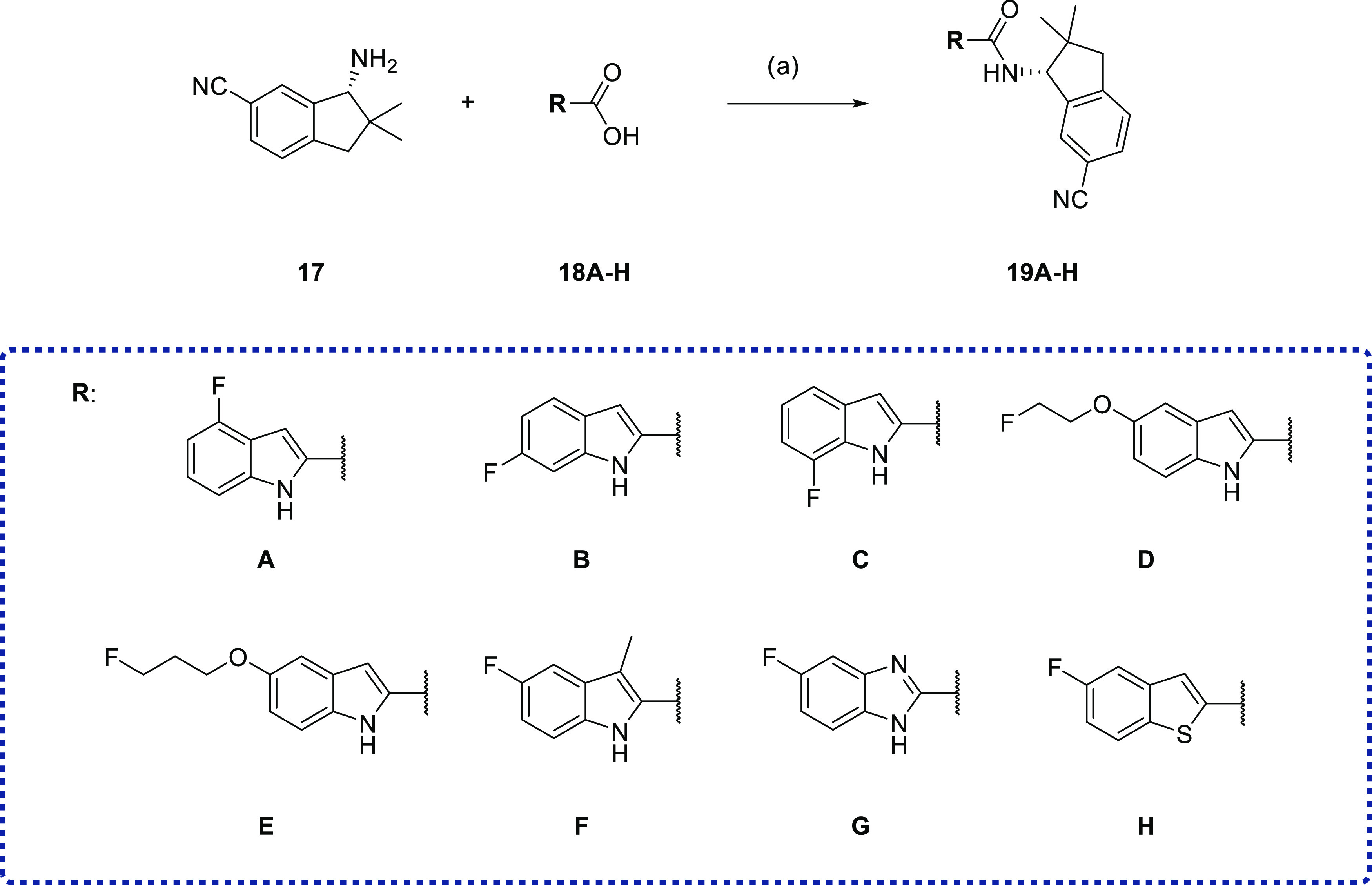
Synthesis of Target Compounds **19A–H** with the Modified 5-Fluoroindole Subunit Reagents and conditions: (a) BOP, NEt_3_, CH_2_Cl_2_, 16 h, rt.

**Scheme 4:**
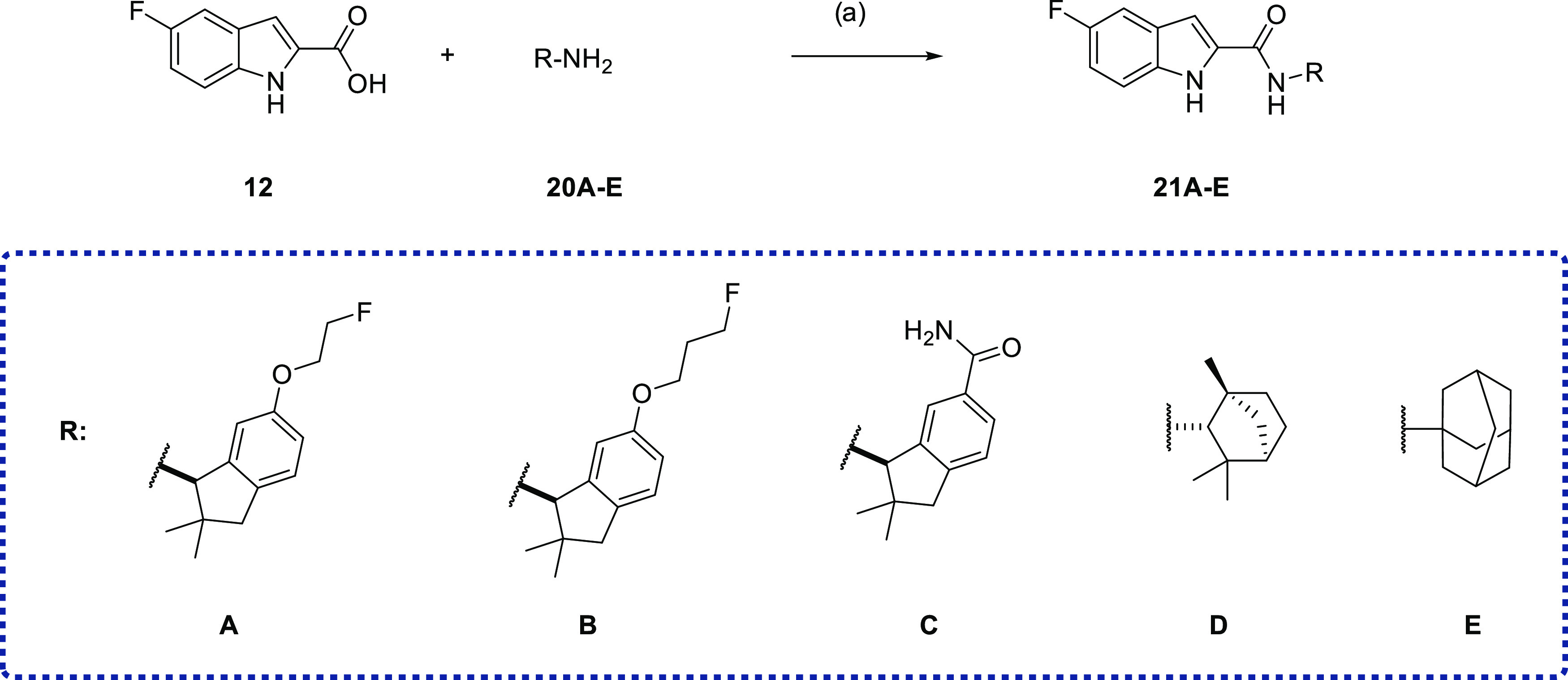
Synthesis of Target Compounds with Modified Indanone Subunit Reagents and conditions: (a) BOP, NEt_3_, CH_2_Cl_2_, 16 h, rt.

By reacting **10** with (*R*)-*tert*-butanesulfinamide (**13**), compound **14** was formed as a single enantiomer. Although examples in the literature show that the NaBH_4_-mediated reduction of such *tert*-butanesulfinyl imines of type **14** can lead to the formation of sulfonamides of type **15** in high diastereomeric excess, in our hands, a 1:1 mixture of diastereomers **15** and **16** was formed. Attempts to increase the diastereomeric excess by strictly controlling the reaction temperature (ranging from −40 to 0 °C) did not improve the diastereoselectivity of the reaction. However, compounds **15** and **16** were readily separated by flash chromatography on silica. TFA-mediated sulfonamide cleavage leads to compound **17**, which was coupled with the carboxylic acid **12** to give (−)-**6** (RM365, Scheme 2). With large amounts of enantiomeric pure amine **17** in our hands, a series of fluorinated derivatives of (−)-**6** was developed by coupling amine **17** with various fluorinated bioisosters of **12** (Scheme 3). A further series of derivatives was synthesized by keeping 5-fluoro-*N*-methyl-1*H*-indole-2-carboxamide intact and modifying the dihydroindene subunit (Scheme 4). First, to evaluate the need for a 5-indole substitution pattern, the regioisomers **19A–C** of RM365 were synthesized. Compounds bearing 5-fluoroethoxy and 5-fluoropropoxy residues (**19D-E**, **21A-B**) were synthesized bearing an aliphatic fluorine. Compounds **19F–H** were synthesized to evaluate the importance of the pyrrole ring for CB2R binding. Compounds **21A**–**D** were synthesized as racemic mixtures by the same procedure (Scheme 4). Compound **21C** was obtained by the hydrolysis of a nitrile group of 6. Furthermore, the dihydroindene subunit or RM365 was replaced by fenchone (**21D**) and adamantine (**21E**) residues, as these are frequently used scaffolds in the medicinal chemistry of CB2R ligands.^31,72^

### *In Vitro* Binding Assay

The binding affinity of the herein described compounds toward the human CB2R was determined by a competitive radioligand binding assay according to the established in-house protocol using cell homogenates obtained from CHO cells stably transfected with hCB2R (hCB2R-CHO).^53,73^ For compounds binding with high affinity to hCB2R, the affinity toward CB1R was determined as well by radioligand displacement studies performed either with homogenates obtained from the mouse brain or hCB1R-CHO cells.

According to the results summarized in Table 1, all derivatives with an (*R*)-configuration at the carboxamide-indenyl subunit bind with low nanomolar to nanomolar affinity toward CB2R and with micromolar affinity toward CB1R. As aforementioned, compound (*−*)-**6** (Scheme 1, Table 1) obtained by chiral HPLC separation of (±)-**6** is characterized by a binding affinity of 2.1 nM, while for the opposite enantiomer (+)-**6**, no binding to the CB2R has been measured in the investigated concentration range. The enantioselectively synthesized compound RM356 (Scheme 2) presented a binding affinity in the same range. Compound’s RM365 regioisomers, **19A–C**, showed low nanomolar CB2R affinity. The introduction of fluoroethoxy and fluoropropoxy residues at the 5-indole position slightly decreased the affinity in comparison to (*R*)-6 (compounds **19D** and **19E**, Table 1). The introduction of a methyl group at the 3-indole position also had a deleterious effect on the binding affinity (**19F**). The substitution of the indole subunit with benzimidazole in **19G** or benzothiophene in **19H** gave compounds with a high CB2R affinity, comparable to that of the starting compound RM365. Compounds **21A** and **21B** were obtained by substituting the nitrile function at the indane subunit with fluoroethoxy and fluoropropoxy, respectively, and showed a slightly reduced affinity toward CB2R. However, the substitution of the indane subunit by fenchone (compound **21D**) and adamantane (compound **21E**) resulted in a loss of CB2R binding.

**Table 1:**
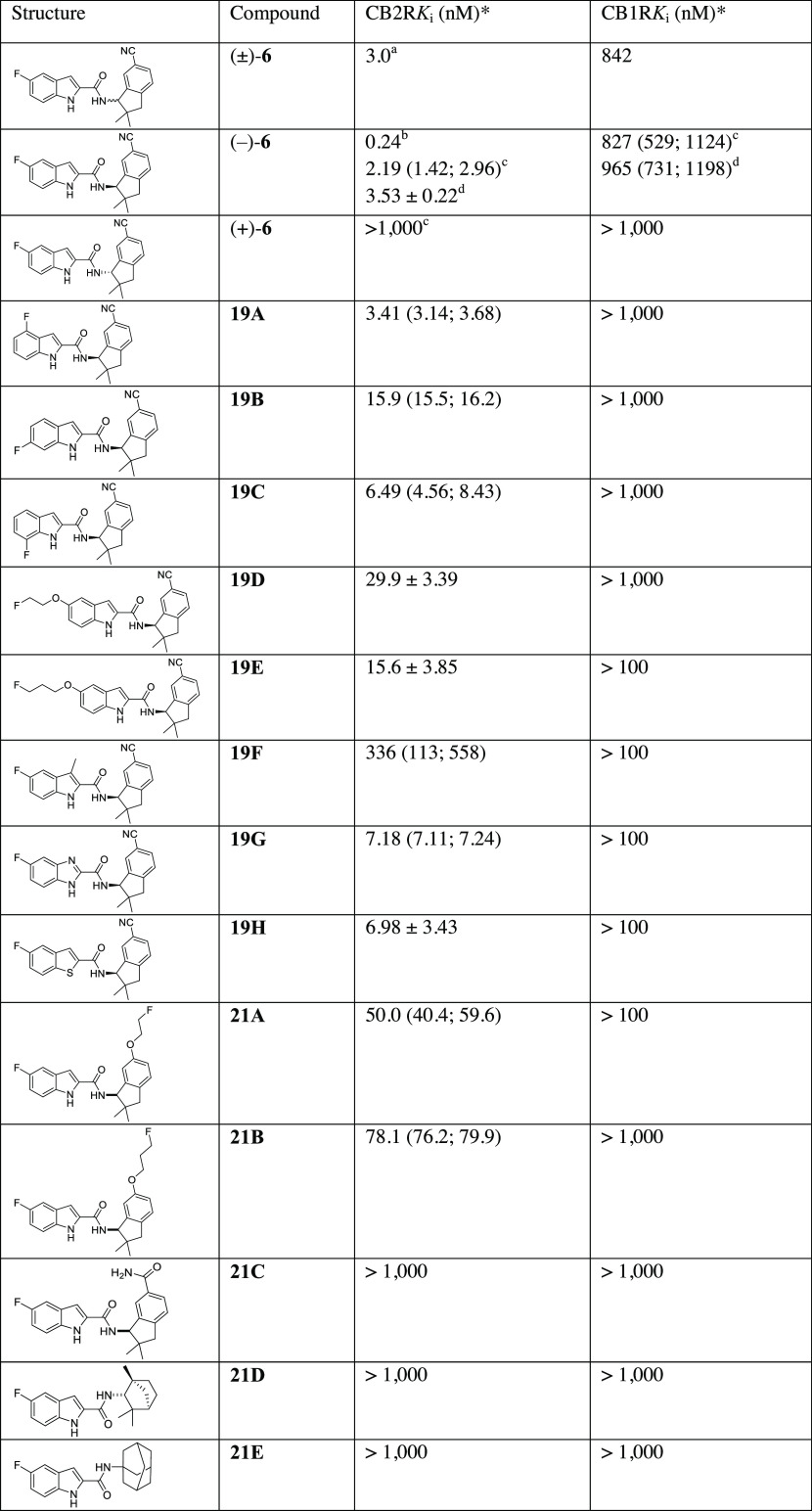
Binding Affinities of Compound 6 and New Derivatives

a Determined in-house according to protocols previously reported by us.^53^

b Reported in ref (60).

c Obtained by chiral HPLC separation (Scheme 1).

d Obtained by enantioselective synthesis (Scheme 2).

* Results are the *K*_i_ values determined in independent experiments, each performed with technical triplicates. CB2R *K*_i_ values were estimated from inhibition curves obtained by displacement of [^3^H]WIN55.2212–2 from hCB2R acquired by homogenization of stably transfected CHO cells. CB1R *K*_i_ values were estimated from inhibition curves obtained by displacement of [^3^H]SR141716A from hCB1R obtained by homogenization of stably transfected CHO cells, respectively.

Single *K*_i_ value was obtained by a single experiment.

Mean of *K*_i_ values of two independent experiments is reported with the single values of each experiment in brackets.

Mean of *K*_i_ values of ≥ three independent experiments is reported with the standard deviation.

### Radiochemistry

**Figure 4:**
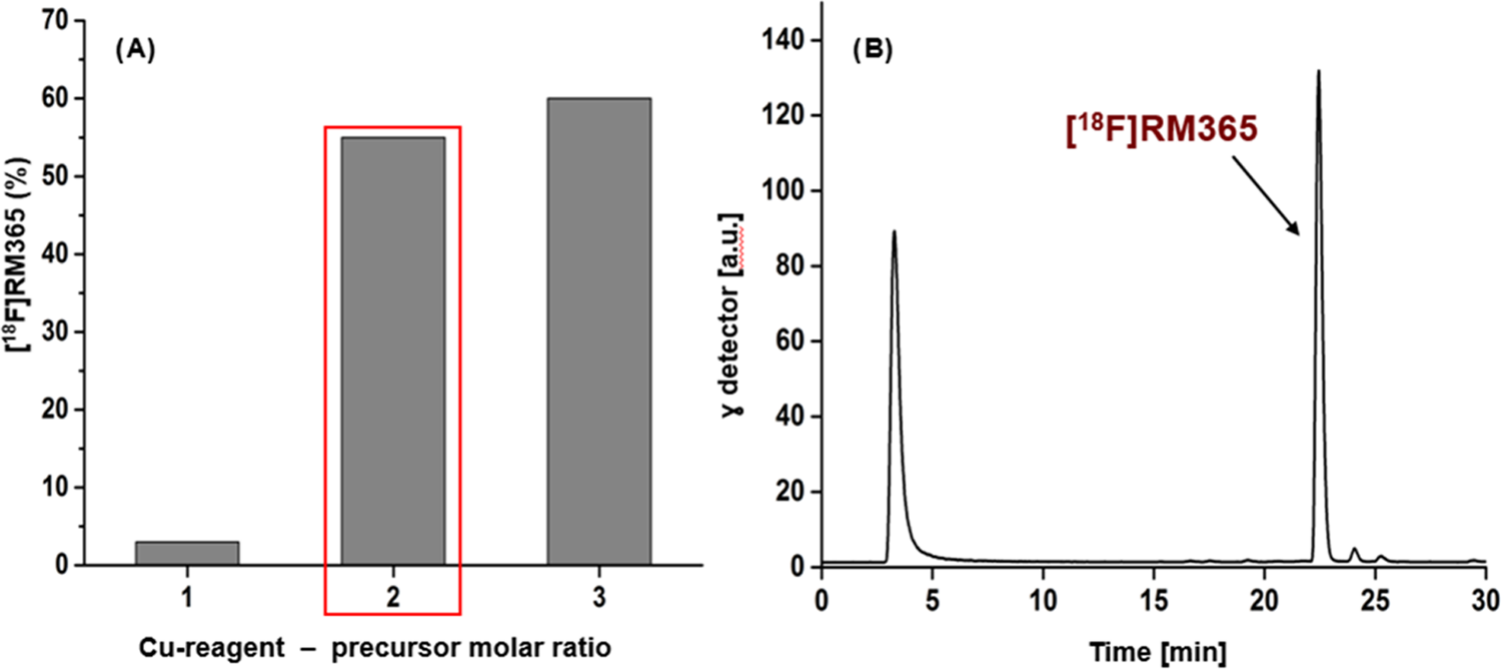
Radiosynthesis of [^18^F]RM365. (A) The influence of the Cu reagent–precursor molar ratio (**25**/**24**) on the yield of the formation of [^18^F]RM365 (yield calculated from the crude reaction mixture, nondecay-corrected). (B) Representative radio-HPLC chromatogram from aliquots of the reaction mixture using 3 mg precursor **24** and a **25**/**24** molar ratio of 2.

**Scheme 5:**
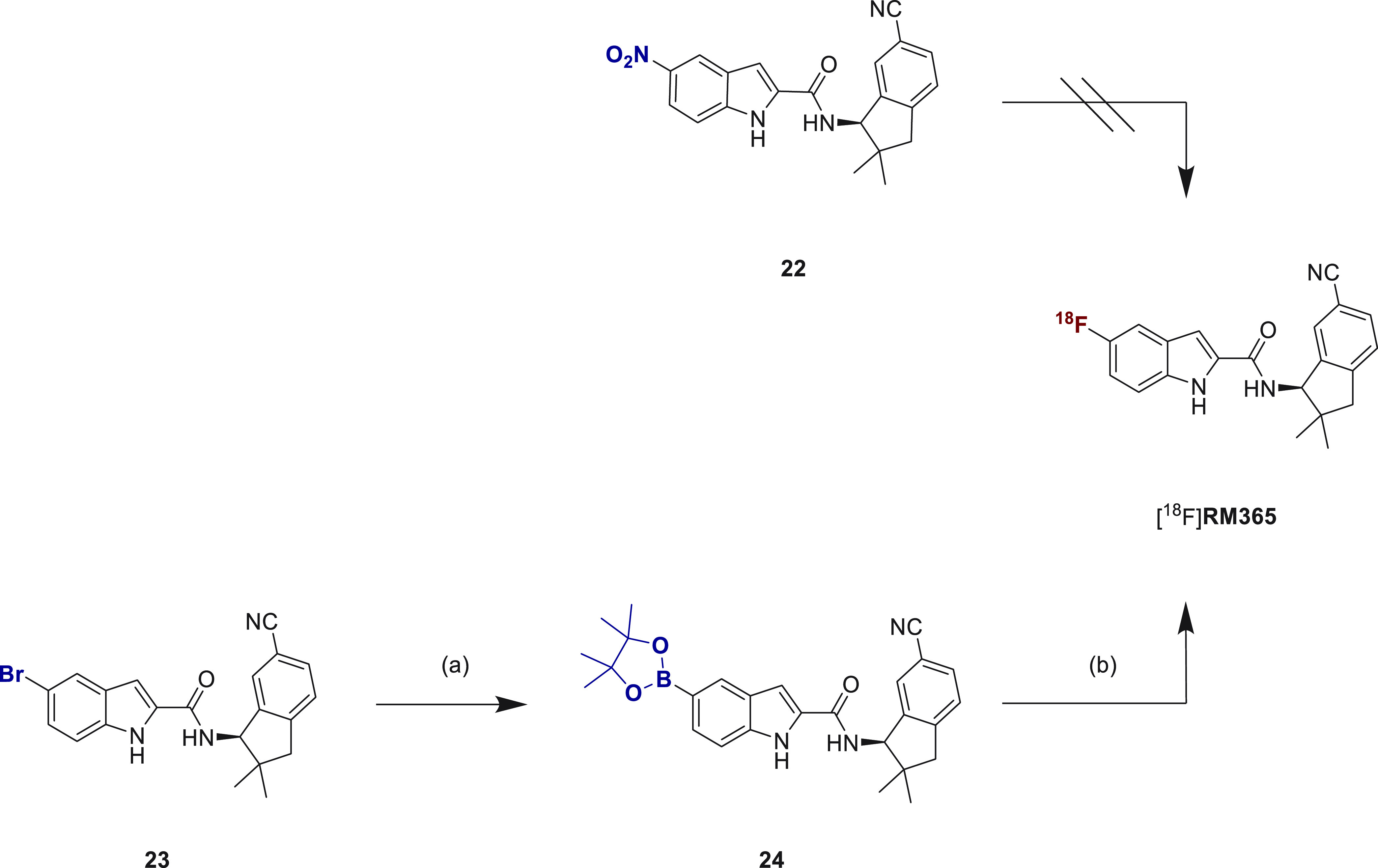
Radiosynthesis of [^18^F]RM365 Reagents and conditions: (a) Pd(dppf)Cl_2_, KOAc, Bis(pinakolato)-diboron, dioxane, 90 °C, 6 h, 85%; (b) [^18^F]TBAF, [Cu(OTf)_2_(Py)_4_] (**25**), DMA, *t*-BuOH, 120 °C, 10 min, 54% (RCC as judged by radio-HPLC analysis of the raw reaction mixture). Abbreviations: [Cu(OTf)_2_(Py)_4_], tetrakis(pyridine)copper(II) triflate; Pd(dppf)Cl_2_, [1,1′-bis(diphenylphosphino)ferrocene]dichloro-palladium(II); DMA, *N*,*N*-dimethylacetamide; KOAc, potassium acetate; TBAF, tetrabutylammonium fluoride; *t*-BuOH, *tert*-butanol; K_222_ 4,7,13,16,21,24-Hexaoxa-1,10-diazabicyclo[8.8.8]hexacosan.

For the radiosynthesis of [^18^F]RM365, a precursor compound bearing a leaving group at the indole 5-position had to be designed. Attempts to radiosynthesize [^18^F]RM365 starting from the 5-nitroindole precursor **22** by using either [^18^F]K_222_/K_2_CO_3_ or [^18^F]TBAF under both thermal and MW irradiation conditions failed. The low reactivity of **22** toward S_N_Ar can be explained by the electron-rich nature of the benzyl subunit of the indole. The S_N_Ar radiofluorination is generally challenging and applicable only for electron-deficient substrates. The introduction of ^18^F at aromatic positions which are not activated for S_N_Ar usually implies multistep radiosynthesis or chemical modifications of the target compounds. Recent advances using transitional metal for the introduction of 18F are however not dependent on the electron density of the aromatic ring, providing good radiolabeling yields for a large variety of substrates.^74^ Accordingly, the formation of [^18^F]RM365 was observed in low to moderate yield by using the boronic acid pinacol ester **24** under the well-known copper-mediated procedure.^75−77^ The boronic acid pinacol ester precursor **24** was synthesized in enantiomeric pure form in one step from the corresponding bromo-derivative **23** via the Miyaura borylation reaction^78^ in high yield (85%) as shown in Scheme 5. To optimize the yield of the radiofluorination reaction, several reaction conditions were tested by varying (a) the amount of precursor (1–5 mg), (b) the Cu reagent–precursor molar ratio (**25**), (c) the order of addition of reagents, (d) the atmosphere (air or argon), and (e) the temperature (110–140 °C) using the [^18^F]TBAF system in DMA and *t*-BuOH as solvents. While the atmosphere used seemed to play no or only a marginal role, the stepwise addition of the Cu reagent to [^18^F]TBAF, followed by the addition of the precursor seemed to greatly improve the formation efficiency of [^18^F]RM365. The time span between the addition of the Cu reagent and the precursor did not influence the labeling yield and a 10 s interval was found to be sufficient. Furthermore, by using an optimal (3 mg) amount of precursor at 120 °C, the critical factor determining the rate of the reaction proves to be the Cu reagent–precursor molar ratio (**25**/**24**) with the best result obtained by using a **25**/**24** ratio of 2 and 3, as shown in Figure 4. To keep the Cu reagent load in the system as low as possible, the following experiments were carried out by using a molar ratio **25**/**24** of 2.

**Figure 5:**
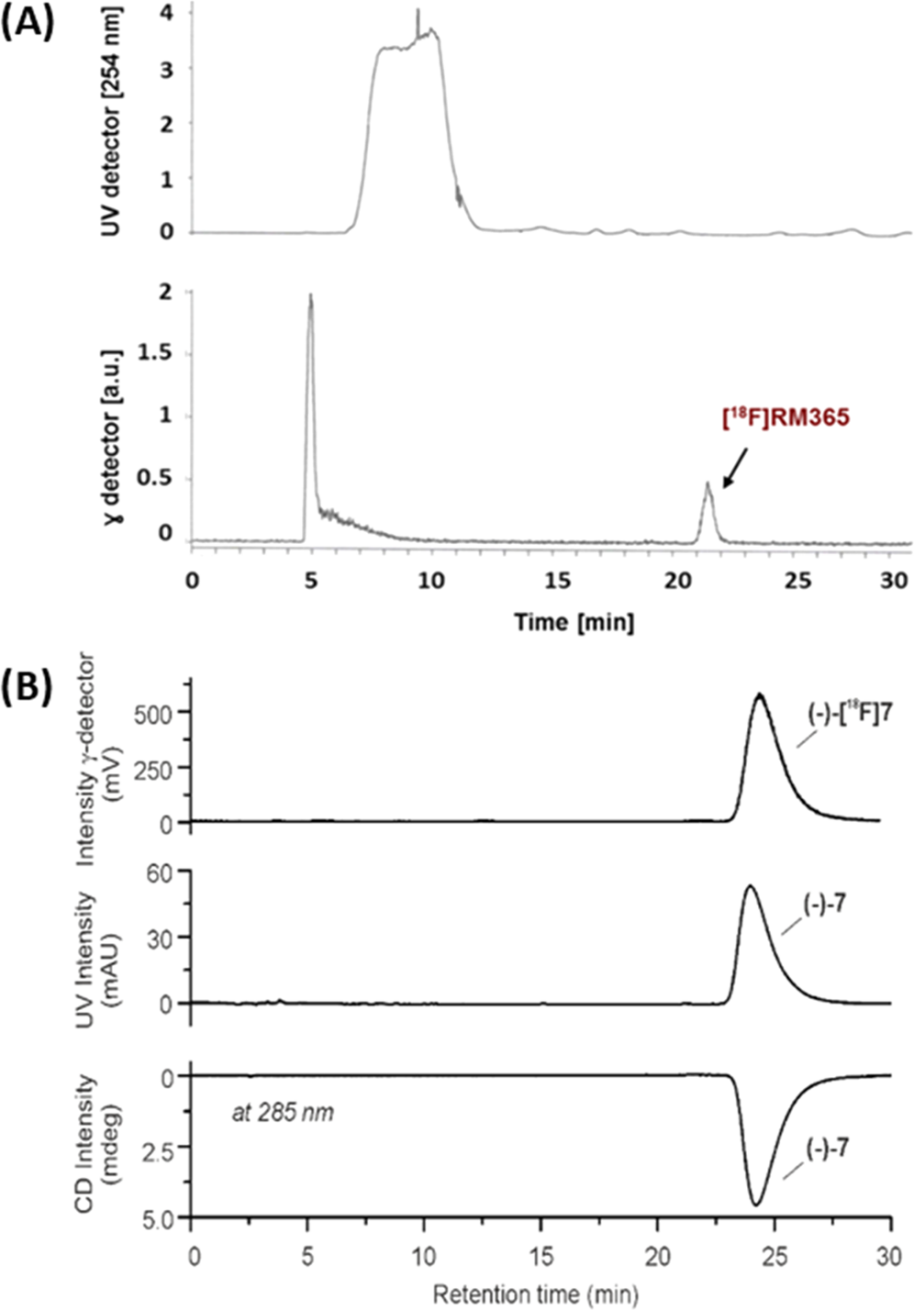
(A) Representative UV- and radio-HPLC chromatograms of [^18^F]RM365 isolation from the crude reaction mixture via semipreparative HPLC (Reprosil-Pur C18-AQ column (250 × 20 mm^2^, 5 μm), 65% MeCN/20 mM NH_4_OAc_aq._, flow: 8.5 mL·min^–1^). (B) Analytical radio-, UV-, and CD-HPLC chromatograms of the final product [^18^F]RM365 spiked with the nonradioactive reference (−)-**6**.

The most efficient protocol for the manual radiosynthesis of [^18^F]RM365 was transferred to an automated synthesis module (Elysia-Raytest radiosynthesizer). For the biological evaluation, [^18^F]RM365 was obtained with a moderate radiochemical yield of about 5% and high radiochemical purities (>99%), molar activity of about 80 GBq/μmol in a total synthesis time of 60 min (*n* = 6) starting with activities ranging from 8 to 13 GBq. A comparable decrease of the radiolabeling efficiency for the automated compared to the manual radiosynthesis for this type of Cu-mediated radiofluorination has been previously reported and extensively discussed in the literature.^79^ RP and chiral HPLC analysis of the final product coeluted with the corresponding reference compound RM365 confirmed the identity and enantiopurity of the radioligand (Figure 5B). A log *D*_7.4_ of 3.0 ± 0.1 was determined by the shake flask method.^80,81^

### *In Vitro* Evaluation of [^18^F]RM365

**Figure 6:**
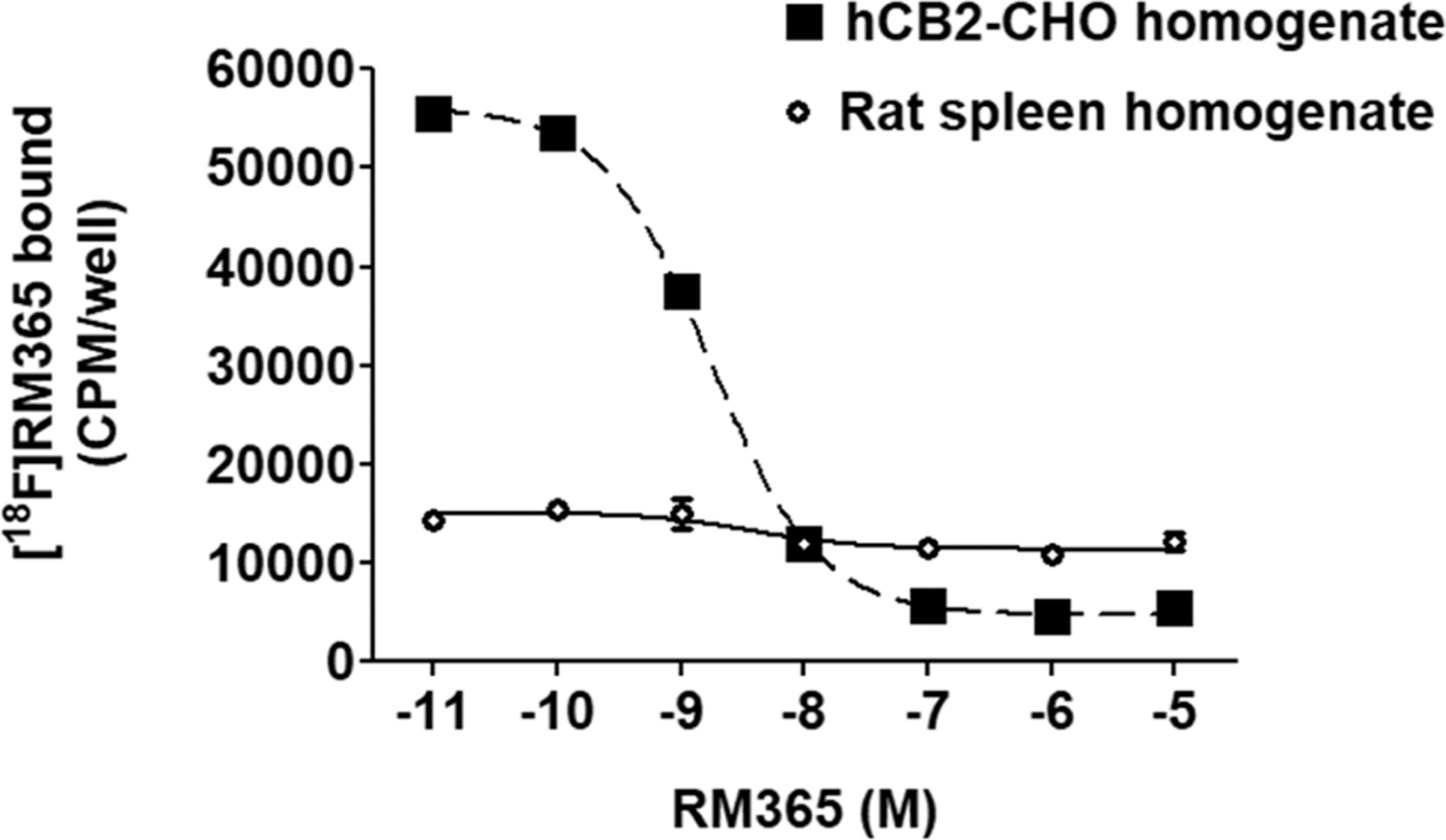
Determination of the equilibrium dissociation constant *K*_D_ of [^18^F]RM365 in vitro. A homologous competition binding experiment was performed by incubating membrane preparations obtained from CHO cells stably transfected with the human CB2R or from the spleen of a female SPRD rat, expressing high levels of CB2R in leukocytes under physiological conditions with 0.078 nM [^18^F]RM365 and increasing concentrations of RM365. Data are obtained from a single experiment performed in triplicate to give IC_50_,_hCB2R_ 2.40 nM (CI_95%_: 1.09–5.26 nM) and *K*_D_,_hCB2R_ 2.32 nM by the simplified Cheng–Prusoff equation *K*_D_ = IC_50_ – concentration radioligand.

To complement the indirect determination of the affinity of RM365 toward the CB2R *in vitro*, we performed a direct binding study with the radiofluorinated ligand using preparations of CHO cells stably transfected with the cloned human receptor and one spleen from a female SPRD rat expressing high levels of the receptor in the leukocytes.^12^ The results of the homologous competition study shown in Figure 6 confirm the good affinity of [^18^F]RM365 toward the human CB2R with a *K*_D_ value of about 2 nM. By contrast, with rat spleen homogenates, no detectable interaction of [^18^F]RM365 with a specific binding site could be observed. Furthermore, the measurement signal obtained from the incubation of [^18^F]RM365 with the homogenate of rat spleen was in the range of the values obtained for the nonspecific binding of the radioligand toward the preparation of CB2R-transfected CHO cells. We conclude that RM365 binds the rat CB2R with lower affinity than the human CB2R but expect no significant interactions of the radioligand with nonspecific binding sites.^82^ Because of the available rat model to assess the potential of [^18^F]RM365 to cross the blood–brain barrier and to specifically label human CB2R—the hCB2R(D80N) model with high expression of human CB2R in the brain^31,32,66,83^—we intended to confirm the initial findings from the rat homogenate by an autoradiographic study. The homogeneous and nondisplaceable distribution pattern of [^18^F]RM365 in cryosections of spleens from two male F433 rats (Figure S2) confirms the absence of a specific interaction of this radioligand with rat CB2R under physiological conditions.

### Molecular Docking

The unexpectedly high human–rat species differences in the binding affinity of [^18^F]RM365 prompted us to investigate the interaction mode of the ligand in the CB2R binding pocket by molecular modeling. *In silico* modeling of the interactions between (−)-**6** and hCB2R and between (−)-**6** and rat CB2R was done by docking of the optimized (at B3LYP D3BJ/def2-TZVP level of theory) structures of RM365 in the binding pockets of the hCB2R and rCB2R, respectively. The crystallographic structure of hCB2R is available from PDB under the code 3ZTY.^84^ The structure of rCB2R was generated by folding the primary sequence in I-TASSER^85^ and optimizing the structure of the best model with the molecular dynamic (see details in the SI).

The calculated binding energies (Δ*G*) demonstrated that the best docked poses of (−)-**6** have a higher affinity to hCB2R (−10.45 kcal mol^–1^) than to rCB2R (−6.64 kcal mol^–1^). The computed inhibitory constants (*EK*_i_) of (−)-**6** calculated based on the equation: Δ*G* = RTln(K_i_) made up of 27 nM for hCB2R and 14 μM for rCB2R, which are in agreement with the experimental data. The explanation of the observed difference could be given based on the binding mode between (−)-**6** and the amino acid residues of the ligand-binding domains (LBDs, Figure S6).

**Figure 7:**
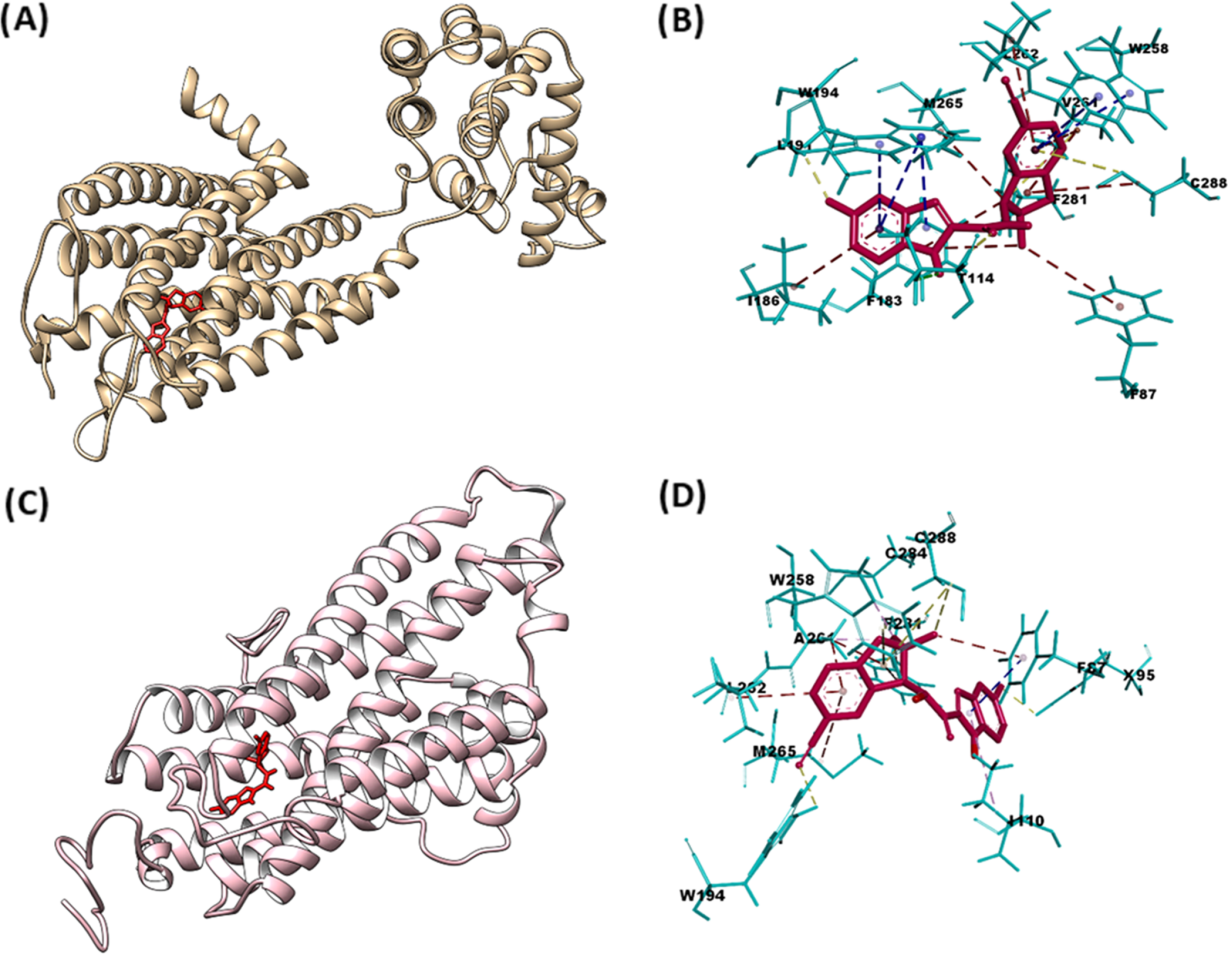
*In silico* investigation of the binding modes of compound (−)-**6** based on docking with hCB2R and rCB2R. The highest ranked docked position of the compound (−)-**6** is colored red in the ligand-binding domains (LBDs) of hCB2R (A) and rCB2R (C). The noncovalent interactions with W194, L191, I186, F183, F114, F87, F281, C288, V261, W258, L262, and M265 in hCB2R (B) and with F87, W258, L262, M265, C288, and A261 in rCB2R (D) of the different type are shown as blue (pi–pi–T interactions), magenta (pi–alkyl type), and yellow (other type) dashed lines. The PDB ID 5ZTY provides access to the crystal structure of the human CB2R used for docking.

Importantly, (−)-**6** forms multiple noncovalent bonds of different types with the ligand-binding domain (LBD) of hCB2R. For instance, the highly ranked pose of (−)-**6** is stabilized by several pi–pi–T-shaped contacts with M265, W194, W258, and V261 (Figure 7B) and pi–alkyl interactions with I186, F87, F281, M265, and C288 (Figure S6).

In contrast to this, the best docked pose of (−)-**6** forms a lower number of interactions with F87, W258, L262, M265, C288, and A261 in the LBD of rCB2R. These noncovalent bonds are dominated by the pi–alkyl interactions. The binding energy indicates the lower stabilizing influence of these contacts on the docked compound in rCB2R compared to multiple pi–pi–T-shaped interactions formed between (−)-**6** and hCB2R. Interestingly, in both receptor subtypes, the best docked poses interact with the toggle switch W258, which was reported as the crucial residue for the activity of CB2R^86^ (Figure 7).

We also investigated the correlation between the binding energies of the best ranked position of the S-isomer and the experimental affinity. Docking suggests a decrease of the binding energy of (+)-**6** inside the LBDs of both hCB2R and rCB2R (−9.71 and −6.15 kcal mol^–1^, respectively), which decreases the affinity of this isomer to both receptors. In general, we observed a different binding mode of (+)-**6** with less noncovalent contacts between (+)-**6** and the LBD amino acids of the receptors. Consequently, this has a lower stabilizing impact on the best ranked poses of (+)-**6** in hCB2R and in rCB2R (see details in the SI).

### *In Vivo* Metabolism

**Figure 8:**
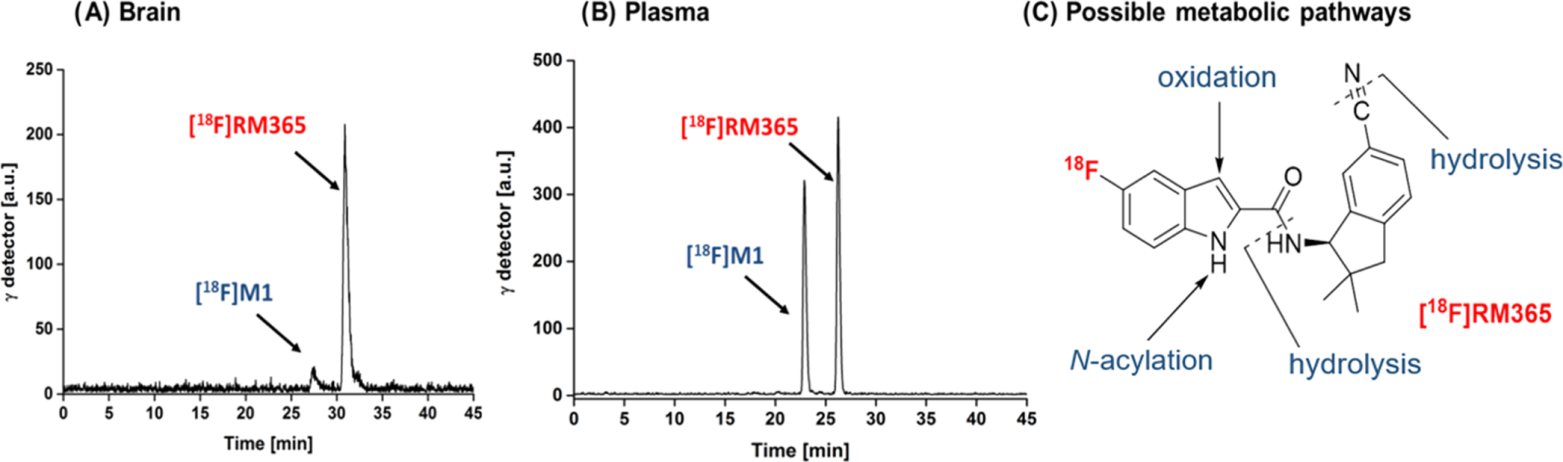
*In vivo* metabolism of [^18^F]RM365. Analytical radio-HPLC chromatograms of tissue and organ samples of female CD-1 mice obtained at 30 min p.i. (A) Brain homogenates [extraction with MeOH/H_2_O (8:2); extraction yield: > 96%]. (B) Blood plasma samples [extraction with MeOH/H_2_O (8:2); extraction yield: > 95%]; HPLC conditions: Reprosil-Pur 120 C18-AQ (5 μm, 250 mm × 4.6 mm); gradient mode (10–90–10% CH_3_CN/20 mM NH_4_OAc_aq_, 1 mL/min).

The good *in vitro* results obtained for [^18^F]RM365 justified its further evaluation *in vivo*, and we proceeded with the investigation of radioligand metabolism in mouse. The brain as well as blood samples were obtained from two female CD-1 mice 30 min after intravenous administration of [^18^F]RM365. The HPLC radiochromatograms obtained from the extracts of the samples are shown in Figure 8. About 90% of the radioactivity detected in the brain samples accounted for intact radioligand (Figure 8), while in blood plasma, the fraction of the intact radioligand represented only 55% of the detected radioactivity (Figure 8B). Accordingly, the absence of a significant fraction of radiometabolites of [^18^F]RM365 in the brain indicates the suitability of this radioligand for neuroimaging.

Regarding the structure of the slightly more polar radiolabeled metabolite M1 of [^18^F]RM365 detected in both plasma and brain samples (Figure 8), we assume that the metabolic degradation might take place at four different positions: (a) hydrolysis of the carboxamide group, (b) hydrolysis of the nitrile group to an amide and/or further to a carboxylic acid, (c) *N*-acylation at the indole, or (d) oxidation of the indole-3-position (Figure 8C). By analyzing commercially available 5-fluoro-1*H*-indole-2-carboxylic acid by HPLC, we could exclude an *in vivo* hydrolysis of the amide group of RM365. However, by hydrolyzing the nitrile group of RM365 with NaOH/H_2_O_2_, we obtained the corresponding amide (**21C**, Table 1) and identified M1 as **21C** from the same *R*_f_ values (not shown). According to the previously determined *K*_i_ value of **21C** (Table 1), the confounding interaction of [^18^F]M1 with hCB2R in PET studies is unlikely. It is, however, unclear why the radiometabolite [^18^F]M1 penetrates brain to a much lower extent as compared to [^18^F]RM365 and if species differences regarding the [^18^F]M1/[^18^F]RM365 ratio in the brain are to be expected. Therefore, prior to a potential human application of [^18^F]RM365, the passive diffusion of [^18^F]M1 and [^18^F]RM365 should be tested in vitro using the tissue of both rodent and human origin.

### PET Imaging

**Figure 9:**
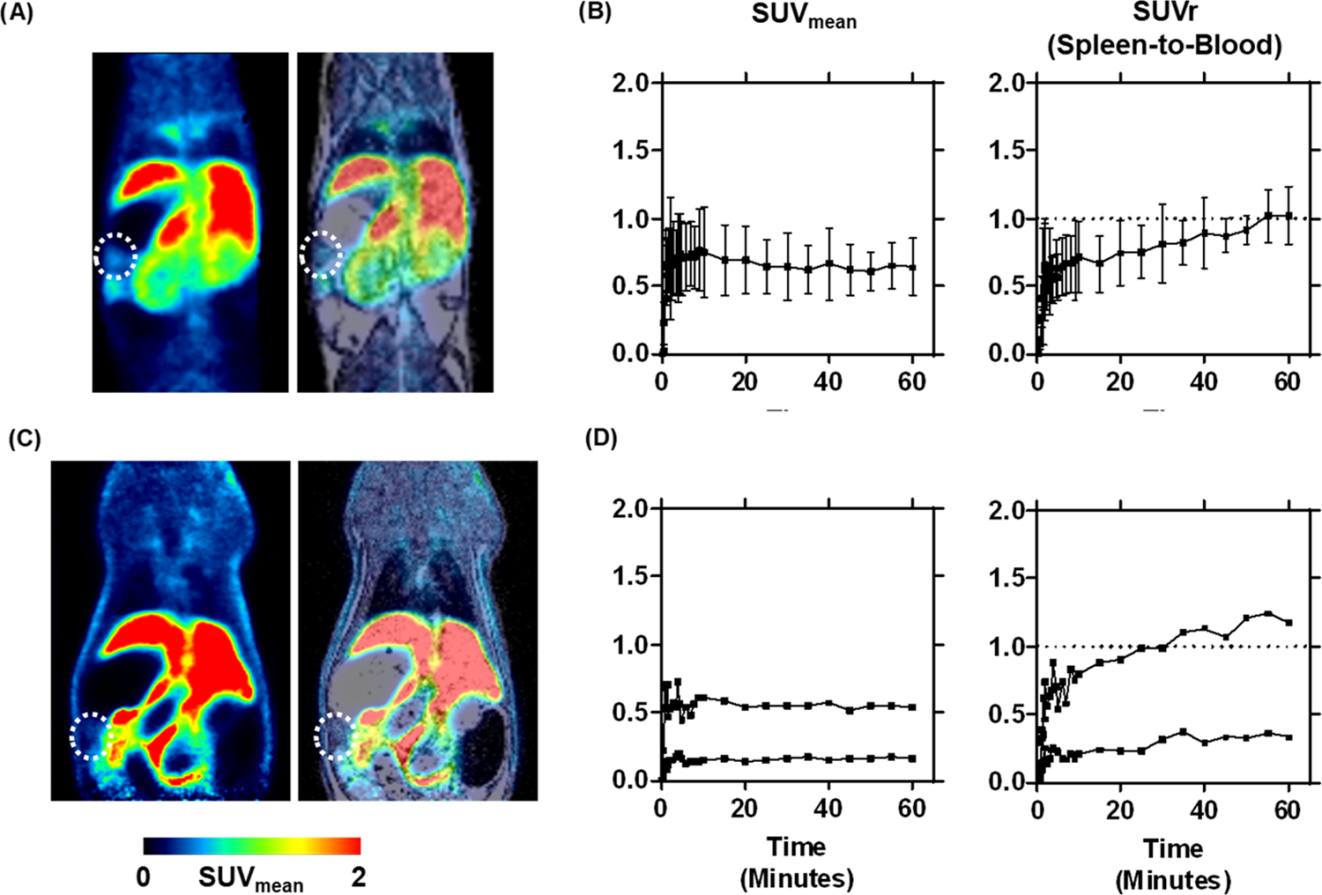
*In vivo* uptake of [^18^F]RM365 into the spleen of CD-1 mice (A, B) and Wistar rats (C, D). (A) and (C) Coronal section of a representative PET image (left), showing the averaged time frames from 0 to 60 min after intravenous administration of [^18^F]RM365 and PET merged with the T1w MR image (right), where the spleen region is marked with a dotted ellipse. (B) and (D) Time–activity curve (TAC) of the radiotracer uptake into the spleen in mean standardized uptake values (SUV_mean_) and the corresponding normalized TAC to the blood activity in the left ventricle (SUVr). Mice: *n* = 3, rats: *n* = 2, mean ± SD.

The biodistribution of [^18^F]RM365 was investigated *in vivo* in mice (*n* = 4) and rats (*n* = 2) by dynamic PET imaging studies. As shown in Figure 9, a constant SUV of 0.7 ± 0.1 between 1 and 60 min was observed in the mouse spleen (Figure 9A,B), which was comparable to rat, although the low number of animals and difference of the maximal SUVs in the plateau phase between 1 and 60 min are drawbacks of the study (Figure 9C,D). The normalized time–activity curve (TAC) of the rodent spleen to the blood TAC (Figure 9B,9D) suggests a low specific enrichment in both species and confirms the results of the *in vitro* binding studies.

The time-dependent uptake into the other tissues is presented in Figures S9 and S11 and was comparable for both investigated species. Among others, increasing activity in the duodenum over time and accumulation in the brown adipose tissue (BAT) were observed (Figure S10). In contrast, a slow washout from the salivary glands, muscle, bones, and kidneys could be observed. An initial comparably high TAC peak value with a SUV_mean_ > 4 in the liver, left ventricle (blood), heart wall, and lung followed by a fast washout and a subsequent slower washout phase could be observed. Furthermore, we conclude a mainly hepatic excretion of the activity, as shown by the liver and jejunum TACs in both species and a bladder activity of 0.24 ± 0.19% of injected dose at 60 min in mice (Figures S8 and S10).

**Figure 10:**
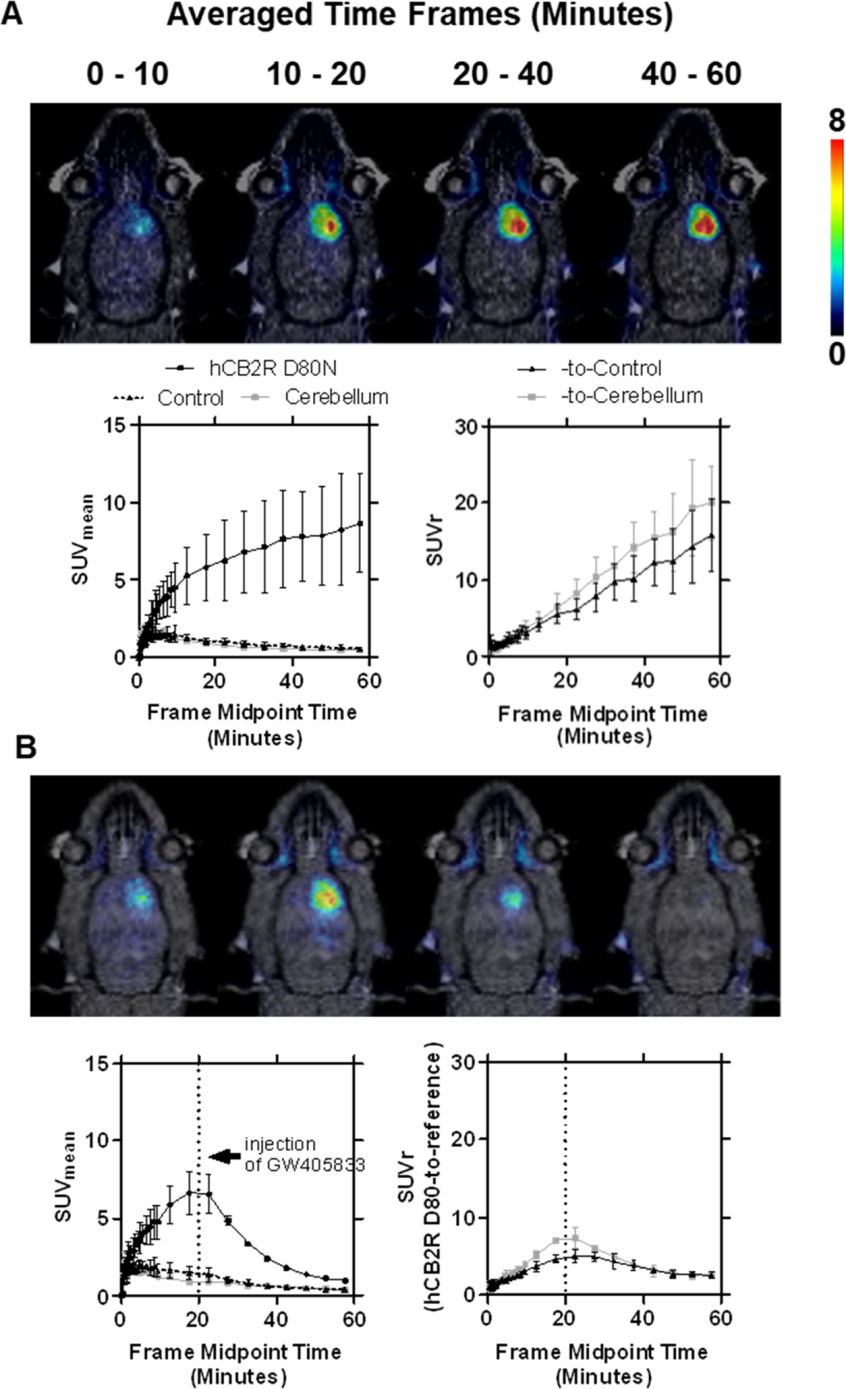
PET imaging of [^18^F]RM365 in a rat model with local hCB2R overexpression in the right striatal region of the brain. Representative coronal planes showing merged MR and PET images of averaged time frames of the control group (A) and displacement study (B) with the corresponding time–activity curves (TACs) of the right (target region, hCB2R D80N) and the reference regions (contralateral and cerebellar regions) expressed in mean standardized uptake values (SUV_mean_), as well as the normalized TACs to the reference regions (SUVr). Compared to the control group, in the displacement study, GW405833 (5 mg/kg bodyweight) was injected i.v. 20 min after administration of [^18^F]RM365. Mean ± SD, *n* = 3.

In a proof-of-concept study, the specific binding to the human CB2R and the ability to cross the blood–brain barrier of [^18^F]RM365 *in vivo* was investigated by PET imaging experiments using an established rat model with a local overexpression of hCB2R(D80N) in the right striatum (target region).^32,66^ Exemplary images of the averaged time frames of the PET study, as well as the TACs of the target region and both reference regions on the contralateral site and the cerebellum, are shown in Figure 10. The noncompartmental (semiquantitive) analysis of the TACs is presented in Table 2. Compared to the CB2R-directed radioligands previously evaluated by us in the same animal model (TAC peak SUV of 3.3 and 3.6 for [^18^F]LU14^83^ and [^18^F]LU13,^31^ respectively), the uptake of [^18^F]RM365 into the target region was more than two times higher, with a SUV of 8.6 ± 3.2 at 60 min postinjection, whereby, further activity accumulation after 60 min could be assumed. However, the TAC peak value was four to five times lower in the reference regions, which was reflected by the seven to eight times lower accumulated activity over time expressed as the area under the curve (AUC) of [^18^F]RM365 (Table 3). The high signal-to-background ratio is reflected by a SUVr of 15 ± 5 and 20 ± 5 for the target region-to-contralateral and -cerebellum ratios, respectively, at 60 min p.i. Hence, the results confirm the blood–brain barrier permeability and the high CB2R specific binding as well as the low signal-to-background ratio of [^18^F]RM365 in the brain of this rat model. Additionally, we investigated the reversibility of the binding toward the human CB2R receptor *in vivo* in displacement studies by administering the CB2R-specific agonist GW405833 into the tail vein 20 min after the radioligand injection into the hCB2R(D80N)-overexpressing rat model (Figure 10B, Table 3). The signal in the target region was reduced 15 min after the injection of GW405833 (*p* < 0.05, Bonferroni’s multiple comparison test) compared to the control group, resulting in a reduction of the normalized AUC by 64 and 70% between 20 and 60 min p.i., using the contralateral site or cerebellum as the reference region, respectively, indicating a reversible binding toward the human CB2R (D80N) receptor. The signal in the contralateral side (*p*-value = 0.1621, two-way ANOVA) and in the cerebellum (*p*-value = 0.6177, two-way ANOVA) was not affected by the treatment over time with GW405833 (Figure S6), underlining the eligibility of these reference regions.

**Table 2:** Noncompartmental (Semiquantitative) Analysis of TACs, Obtained from the Regions of Local Overexpression of hCB2R(D80N) in the rRight Striatum of Rats, the Contralateral Region in the Left Striatum, and the Cerebellum After i.v. Injection of [^18^F]RM365 (*n* = 3)

TAC parameter	hCB2R(D80N)	contralateral	cerebellum	*p*-value (contralateral vs cerebellum)
time-to-peak (min)	>60	3.6 ± 0.8	1.9 ± 1.2	0.054
TAC peak value (SUV_mean_)	8.6 ± 3.2 at 60 min p.i.^a^	1.6 ± 0.3	2.0 ± 0.3	0.148
AUC (SUV·min)	364 ± 141	51 ± 12	44 ± 7	0.218

Mean ± SD; *p*-value, one-sided Student’s *t*-test; TAC, time–activity curve, AUC, area under the curve 0–60 min p.i.

a TAC peak value not reached in the observation period.

**Table 3:** Uptake of [^18^F]RM365 into the hCB2R(D80N) Overexpressing Right Striatum Normalized to the Contralateral Left Striatum and Cerebellum (SUV Ratio, SUVr) With (*n* = 3) or Without (vehicle, *n* = 3) Administration of GW405833 (5 mg/kg Bodyweight, i.v.) at 20 min After Radiotracer Administration, Expressed as Area under the Curve Before (AUC_0-20 min_) and After (AUC_20-60 min_) Injection of the Interventional Drug

hCB2R D80N-to-	treatment	AUC_0–20 min_ (CI_95%_) in SUVr·min	AUC_20–60 min_ (CI_95%_) in SUVr·min
contralateral	vehicle	55 (42–68)	419 (288–550)
	displacement	49 (44–54)	151 (125–177) – 63.9%
cerebellum	vehicle	60 (45–75)	543 (384–703)
	displacement	65 (59–71)	164 (150–178) – 69.8%

Mean; 95% confidence interval (CI_95%_).

Taken together, the present *in vivo* study demonstrates a specific and reversible binding of [^18^F]RM365 toward the human CB2R. The high uptake into the target region and high signal-to-background ratio in the rat brain reveals the high potential of [^18^F]RM365 for the noninvasive assessment of disease-associated upregulation of CB2R in the human brain by PET.

## Conclusions

A novel series of indole-2-carboxamides was developed and evaluated for CBR binding properties *in vitro*. The compound with the best CB2R binding affinity, RM365 ((−)-**6**), was radiolabeled with fluorine-18 and further biologically characterized. *In vitro* binding experiments revealed a high affinity of [^18^F]RM365 for hCB2R and no or low affinity for rCB2R. Attempts to resolve the binding mechanism by *in silico* modeling indicated the higher stability of the interactions between RM365 and hCB2R than RM365 and rCB2R. *In vivo* evaluation in rodents revealed high metabolic stability with >90% intact [^18^F]RM365 determined at 30 min p.i. in the mouse brain. Furthermore, PET scans with a rat model of local hCB2R overexpression demonstrated that [^18^F]RM365 is able to selectively and reversibly label cerebral CB2R. Thus, [^18^F]RM365 is a novel PET radioligand suitable for the imaging of human CB2R in the brain.

## Experimental Section

### General Information

All chemicals and reagents were purchased from commercial sources and used without further purification. Moisture-sensitive reactions were conducted under argon with oven-dried glassware and anhydrous solvents. Reaction progress was monitored by thin-layer chromatography (TLC) using Alugram SIL G/UV_254_ precoated plates (Macherey-Nagel; Düren; Germany). The spots were identified by using a UV lamp or by dipping the plates into a potassium permanganate solution (3 g of KMnO_4_, 20 g of K_2_CO_3_, 0.25 mL of glacial acid, 300 mL of water). For purification of products, flash column chromatography was used with silica gel 40–63 μm (VWR International Chemicals, Darmstadt; Germany). ^1^H-, ^13^C-, and ^19^F-NMR spectra were recorded on VARIAN Mercury plus (300 MHz for ^1^H NMR, 75 MHz for ^13^C NMR, 282 MHz for ^19^F-NMR) and BRUKER DRX-400 (400 MHz for ^1^H NMR, 100 MHz for ^13^C NMR, 377 MHz for ^19^F-NMR); chemical shifts (δ) in parts per million (ppm) are related to internal tetramethylsilane and coupling constants (*J*) are given with 0.1 Hz. High-resolution mass spectra (HRFT-MS) were recorded on an FT-ICR APEX II spectrometer (Bruker Daltonics; Bruker Corporation; Billerica; USA) using electrospray ionization (ESI). The purity of all of the tested compounds was ≥95% as determined by HPLC [Jasco, MD-2010Plus, LG-2080-04S, DG-2080-54, AS-2055Plus, LC-NetII/ADC, λ = 280 nm, column ReproSil-Pur Basic C18-HD (250 × 4.6 mm, 5 μm, Dr. Maisch GmbH), gradient MeCN/20 mMAA from 10/90 to 90/10, to 10/90 (v/v) over 30 min, flow rate 1 mL/min].

### Chemical Synthesis

#### (*rac*)-*N*-(6-Cyano-2,2-dimethyl-2,3-dihydro-1*H*-inden-1-yl)-5-fluoro-1*H*-indole-2-carboxamide ((±)**-6**)

A solution of 11 (100 mg, 0.53 mmol, 1 equiv) and 12 (96 mg, 0.53 mmol, 1 equiv) in DCM (20 mL) was treated with BOP (351 mg, 0.75 mmol, 1.5 equiv) and Et_3_N (220 mL, 1.59 mmol, 3 equiv) at rt for 16 h. Upon completion of the reaction (TLC, EA/Hex. 1:4), NaHCO_3_ (2% aq, 20 mL) was added, vigorously stirred, and separated, and the aqueous phase was washed one more time with 15 mL of DCM. The combined organic phases were dried over MgSO_4_, the solvent was evaporated under reduced pressure, and the resulting residue was purified by flash chromatography on silica (SiO_2_, EA/Hex, 1:4) to give compound (±)-6 (135 mg, 0.39 mmol, 73% yield). ^1^H NMR (400 MHz, CDCl_3_) δ 9.82 (s, 1H), 7.53 (d, *J* = 4.1 Hz, 2H), 7.43 (dd, *J* = 9.0, 4.3 Hz, 1H), 7.34 (m, 1H), 7.30 (dd, *J* = 9.7, 2.6 Hz, 1H), 7.09 (td, *J* = 9.1, 2.4 Hz, 1H), 6.94 (d, *J* = 1.6 Hz, 1H), 6.43 (d, *J* = 9.8 Hz, 1H), 5.57 (d, *J* = 9.8 Hz, 1H), 2.91 (q, *J* = 16.4 Hz, 2H), 1.70 (m, 1H), 1.39 (s, 3H), 1.06 (s, 3H).

Analytical chiral chromatographic measurements of (±)-**6**, (−)-**6**, and (+)-**6** were performed on a JASCO LC-2000 system, incorporating a PU-2080*Plus* pump, an AS-2055*Plus* auto injector (100 μL sample loop), and a DAD detector MD-2010 (wavelength range 195–650 nm) coupled with the chiral detector OR-2090, which was used for the polarimetric assignment of the enantiomers (JASCO Deutschland GmbH, Pfungstadt, Germany) or on a JASCO LC-4000 system incorporating a PU-4180-LPG pump, an AS-4050 auto injector (100 μL sample loop), and a UV-diode array detector MD-4015 (monitoring at 254 nm) coupled with a circular dichroism chiral detector CD-4095. Data analysis was performed with Galaxie chromatography software (Agilent Technologies) or ChromNAV 2.3C software (JASCO Deutschland GmbH, Pfungstadt, Germany). For the chiral separations, a CHIRALPAK IA column (250 × 4.6 mm, 5 μm, Daicel, Chiral Technologies Europe, France) was used in isocratic mode with 58% ACN/aq. Twenty mM NH_4_OAc was used as the mobile phase and the flow rate was 1 mL/min. The CD spectra were recorded with the CD-4095 detector during the HPLC run in stopped-flow mode at which the flow through the detector cell was bypassed using a software-controlled switching valve. Each spectrum was obtained with a scanning speed of 10 nm/s.

For the semipreparative chiral separation of (±)-**6**, a Merck-Hitachi Model D-6000 with an L-6100 pump, a Rheodyne injection valve with a 4 mL sample loop, and an L-6400 UV detector (monitoring at 254 nm) were used. The separation was performed on a CHIRALPAK IA column (250 × 10 mm) using the same eluent conditions as for the analytical separation. When 3.5 mg of the racemate dissolved in 500 μL of MeCN was injected at a flow rate of 3.0 mL/min, the plus-enantiomer eluted with a *t*_R_ = 27 min and the minus-enantiomer with a *t*_R_ = 29 min. They could be collected without impurities of the other enantiomer. Conveniently, this sample was injected several times every 30 min, without stopping the run.

#### *N*-((*R*)-6-Cyano-2,2-dimethyl-2,3-dihydro-1*H*-inden-1-yl)-2-methylpropane-2-sulfinamide (**14**)

Ti(OEt)_4_ (500 μL, 2.1 mmol, 2 equiv) and compound **13** (130 mg, 2 mmol, 1 equiv) were added to a solution of compound **10** (200 mg, 1 mmol, 1 equiv) in toluol (20 mL) and the whole was heated at 70 °C for 2 h. After cooling to room temperature, H_2_O (20 mL) was added, the precipitate was removed by filtration, and the resulting aqueous phase was washed three times with EtOAc (3 × 10 mL). The combined organic solutions were dried over MgSO_4_, and the solvent was evaporated under reduced pressure. The resulting residue was dissolved in THF (20 mL) and cooled to −40 °C, and NaBH_4_ (82 mg, 2.1 mmol, 2 equiv) was added. The reaction temperature was kept at −40 °C after which it was allowed to slowly warm up to rt overnight. An aqueous NaHCO_3_ solution (2% aq, 20 mL) and DCM (20 mL) were added, vigorously stirred, and separated. The resulting aqueous phase was washed one more time with DCM (10 mL). The combined organic solutions were dried over MgSO_4_, the solvent was evaporated under reduced pressure, and the resulting residue was purified by flash chromatography on silica (SiO_2_, EA/Hex, 1:4 to 1:1) to give compounds **15** (first eluting fraction, 80 mg, 0.03 mmol, 31%) and **16** (second eluting fraction, 75 mg, 0.03 mmol, 29%).

Compound **14**, TLC (silica gel, EA/IH, 1:1): *R*_f_ = 0.45; ^1^H NMR (400 MHz, CDCl_3_) δ 7.90 (d, *J* = 1.8 Hz, 1H), 7.70 (dd, *J* = 8.1, 1.9 Hz, 1H), 7.33 (d, *J* = 8.1 Hz, 1H), 2.96 (s, 2H), 1.25 (s, 8H).

Compound **15**, TLC (silica gel, EA/IH, 1:1): *R*_f_ = 0.35; ^1^H NMR (400 MHz, CDCl_3_) δ 7.90 (d, *J* = 1.8 Hz, 1H), 7.70 (dd, *J* = 8.1, 1.9 Hz, 1H), 7.33 (d, *J* = 8.1 Hz, 1H), 2.96 (s, 2H), 1.25 (s, 8H).

#### (*R*)-3-amino-2,2-dimethyl-2,3-dihydro-1*H*-indene-5-carbonitrile (**17**)

A solution of compound **14** (50 mg, 0.2 mmol) in DCM (0.5 mL) was then treated with TFA (100 μL) at room temperature for 1 h. The solvent was evaporated, and the residue (**17**, 37 mg, 0.2 mmol) was used in the next step without purification.

^1^H NMR (300 MHz, CDCl_3_) δ 7.62 (s, 1H), 7.50 (dd, *J* = 7.7, 1.3 Hz, 1H), 7.32–7.21 (m, 1H), 4.71 (s, 1H), 2.75 (q, *J* = 16.3 Hz, 2H), 2.09 (s, 1H), 1.20 (s, 3H), 0.99 (s, 3H).

### General Procedure for the Synthesis of the Target Compounds **19A–H** and **21A–E**

A solution of the corresponding amine (1 equiv) and carboxylic acid (1 equiv) in DCM was treated with BOP (1.5 equiv) and Et_3_N (3 equiv) at rt for 16 h. Upon completion of the reaction, NaHCO_3_ (2% aq) was added, vigorously stirred, and separated, and the aqueous phase was washed one more time with DCM. The combined organic phases were dried over MgSO_4_, the solvent was evaporated under reduced pressure, and the resulting residue was purified by flash chromatography on silica.

#### (*R*)-*N*-(6-Cyano-2,2-dimethyl-2,3-dihydro-1*H*-inden-1-yl)-5-fluoro-1*H*-indole-2-carboxamide ((−)-**6**)

^1^H NMR (400 MHz, CDCl_3_) δ 9.76 (s, 1H), 7.54–7.48 (m, 2H), 7.41 (dd, *J* = 9.0, 4.4 Hz, 1H), 7.34–7.24 (m, 2H), 7.06 (td, *J* = 9.1, 2.5 Hz, 1H), 6.91 (d, *J* = 1.6 Hz, 1H), 6.38 (d, *J* = 9.8 Hz, 1H), 5.54 (d, *J* = 9.8 Hz, 1H), 2.89 (q, *J* = 16.4 Hz, 2H), 1.36 (s, 3H), 1.04 (s, 3H). ^13^C NMR (101 MHz, CDCl_3_) δ 161.67, 159.39, 157.04, 147.70, 143.78, 133.17, 132.15, 131.52, 127.75, 126.00, 118.94, 113.88 (d, *J* = 26.9 Hz), 112.98 (d, *J* = 9.6 Hz), 110.64, 106.24 (d, *J* = 23.4 Hz), 102.39 (d, *J* = 5.2 Hz), 61.93, 45.92, 45.61, 26.88, 22.25. HRMS (ESI^+^): *m*/*z* = 348.1056, calcd. 348.1057 for C_21_H_19_FN_3_O^+^ [M + H]^+^; [α]_*D*_^21^ = −47° (c = 1 mg/mL DMSO).

#### (*R*)-*N*-(6-Cyano-2,2-dimethyl-2,3-dihydro-1*H*-inden-1-yl)-4-fluoro-1*H*-indole-2-carboxamide (**19A**)

^1^H NMR (400 MHz, CDCl_3_/MeOD 9:1) δ 7.52 (d, *J* = 6.5 Hz, 2H), 7.31 (d, *J* = 8.2 Hz, 1H), 7.21 (dt, *J* = 7.6, 6.6 Hz, 1H), 7.09 (s, 1H), 6.82–6.73 (m, 1H), 5.48 (s, 1H), 2.87 (d, *J* = 6.2 Hz, 4H), 2.03 (s, 1H), 1.33 (s, 3H), 1.02 (s, 3H). ^13^C NMR (101 MHz, CDCl_3_) δ 161.88, 158.14, 155.66, 147.95, 143.80, 132.08, 127.85, 125.96, 125.10 (d, *J* = 7.7 Hz), 119.01, 119.01, 110.35, 108.14 (d, *J* = 3.9 Hz), 104.84 (d, *J* = 18.5 Hz), 99.09, 61.86, 45.90, 45.61, 29.65, 26.94, 22.20. HRMS (ESI^+^): *m*/*z* = 348.1055, calcd. 348.1057 for C_21_H_19_FN_3_O^+^ [M + H]^+^.

#### (*R*)-*N*-(6-Cyano-2,2-dimethyl-2,3-dihydro-1*H*-inden-1-yl)-6-fluoro-1*H*-indole-2-carboxamide (**19B**)

^1^H NMR (400 MHz, CDCl_3_) δ 10.02 (s, 1H), 7.53 (m, 2H), 7.34 (d, *J* = 7.6 Hz, 1H), 7.17 (d, *J* = 9.1 Hz, 1H), 6.97 (m, 1H), 6.47 (d, *J* = 9.5 Hz, 1H), 5.59 (d, *J* = 9.7 Hz, 1H), 4.14 (dd, *J* = 14.1, 7.0 Hz, 1H), 2.92 (q, *J* = 16.4 Hz, 2H), 2.06 (s, 1H), 1.40 (s, 3H), 1.07 (s, 3H). ^13^C NMR (101 MHz, CDCl_3_) δ 162.56, 161.89, 160.16, 147.80, 143.85, 136.77 (d, *J* = 12.9 Hz), 132.11, 130.61 (d, *J* = 3.4 Hz), 127.79, 126.00, 124.12, 123.13 (d, *J* = 10.3 Hz), 119.02, 110.35 (d, *J* = 25.3 Hz), 102.84, 98.06 (d, *J* = 26.1 Hz), 61.94, 45.95, 45.72, 26.92, 22.30. HRMS (ESI^+^): *m*/*z* = 348.1055, calcd. 348.1057 for C_21_H_19_FN_3_O^+^ [M + H]^+^.

#### (*R*)-*N*-(6-Cyano-2,2-dimethyl-2,3-dihydro-1*H*-inden-1-yl)-6-fluoro-1*H*-indole-2-carboxamide (**19C**)

^1^H NMR (400 MHz, CDCl_3_) δ 9.74 (s, 1H), 7.55 (s, 1H), 7.52 (d, *J* = 7.8 Hz, 1H), 7.43 (d, *J* = 7.8 Hz, 1H), 7.34 (d, *J* = 7.7 Hz, 1H), 7.05 (m, 3H), 6.42 (d, *J* = 9.7 Hz, 1H), 5.59 (d, *J* = 9.8 Hz, 1H), 2.91 (q, *J* = 16.4 Hz, 2H), 2.06 (s, 1H), 1.38 (s, 3H), 1.07 (s, 3H). ^13^C NMR (101 MHz, CDCl_3_) δ 161.44, 150.94, 148.49, 147.77, 143.85, 132.13, 130.95, 130.94, 127.81, 125.99, 125.38 (d, *J* = 13.8 Hz), 121.03 (d, *J* = 5.8 Hz), 119.00, 117.69 (d, *J* = 3.7 Hz), 110.60, 109.22 (d, *J* = 15.8 Hz), 102.98, 61.91, 45.94, 45.67, 26.82, 22.28. HRMS (ESI^+^): *m*/*z* = 348.1058, calcd. 348.1057 for C_21_H_19_FN_3_O^+^ [M + H]^+^.

#### (*R*)-*N*-(6-Cyano-2,2-dimethyl-2,3-dihydro-1*H*-inden-1-yl)-5-(2-fluoroethoxy)-1*H*-indole-2-carboxamide (**19D**)

^1^H NMR (400 MHz, CDCl_3_) δ 9.14 (s, 1H), 7.55 (m, 2H), 7.40 (d, *J* = 8.8 Hz, 1H), 7.35 (d, *J* = 7.8 Hz, 1H), 7.11 (d, *J* = 2.3 Hz, 1H), 7.07 (dd, *J* = 8.9, 2.4 Hz, 1H), 6.84 (d, *J* = 1.5 Hz, 1H), 6.21 (d, *J* = 9.8 Hz, 1H), 5.54 (d, *J* = 9.9 Hz, 1H), 4.90–4.69 (m, 2H), 4.38–4.19 (m, 2H), 2.91 (q, *J* = 16.4 Hz, 2H), 2.06 (s, 1H), 1.38 (s, 3H), 1.05 (s, 1H). ^13^C NMR (76 MHz, CDCl_3_) δ 161.8, 153.9, 147.6, 143.9, 132.1, 131.9, 130.4, 127.9, 127.7, 125.9, 118.9, 116.6, 112.9, 110.7, 103.5, 102.0, 82.0, 61.8, 45.7 (d, *J* = 21.5 Hz), 30.5 (d, *J* = 19.9 Hz), 29.7, 26.8, 22.2. HRMS (ESI^+^): *m*/*z* = 392.1678, calcd. 392.1679 for C_23_H_23_FN_3_O_2_^+^ [M + H]^+^.

#### (*R*)-*N*-(6-Cyano-2,2-dimethyl-2,3-dihydro-1*H*-inden-1-yl)-5-(3-fluoropropoxy)-1*H*-indole-2-carboxamide (**19E**)

^1^H NMR (400 MHz, CDCl_3_) δ 9.30 (s, 1H), 7.52 (d, *J* = 8.3 Hz, 1H), 7.51 (s, 1H), 7.36 (d, *J* = 8.9 Hz, 1H), 7.32 (d, *J* = 7.7 Hz, 1H), 7.06 (d, *J* = 2.3 Hz, 1H), 6.99 (dd, *J* = 8.9, 2.4 Hz, 1H), 6.83 (d, *J* = 1.5 Hz, 1H), 6.25 (d, *J* = 9.8 Hz, 1H), 5.52 (d, *J* = 9.8 Hz, 1H), 4.68 (dt, *J* = 47.1, 5.8 Hz, 2H), 4.13 (t, *J* = 6.1 Hz, 2H), 2.88 (q, *J* = 16.4 Hz, 2H), 2.20 (dp, *J* = 26.0, 6.0 Hz, 2H), 2.04 (s, 1H), 1.35 (s, 3H), 1.03 (s, 3H). ^13^C NMR (76 MHz, CDCl_3_) δ 161.79, 153.92, 147.66, 143.94, 132.15, 131.93, 130.42, 127.95, 127.76, 125.99, 118.98, 116.62, 112.91, 110.70, 103.54, 102.03, 82.02, 64.17 (d, *J* = 5.2 Hz), 61.84, 45.78 (d, *J* = 21.5 Hz), 30.55 (d, *J* = 19.9 Hz), 29.71, 26.83, 22.24. HRMS (ESI^+^): *m*/*z* = 406.1926, calcd. 406.1926 for C_24_H_25_FN_3_O_2_^+^ [M + H]^+^.

#### (*R*)-*N*-(6-Cyano-2,2-dimethyl-2,3-dihydro-1*H*-inden-1-yl)-5-fluoro-3-methyl-1*H*-indole-2-carboxamide (**19F**)

^1^H NMR (400 MHz, CDCl_3_) δ 9.37 (s, 2H), 7.43 (d, *J* = 7.2 Hz, 4H), 7.32–7.21 (m, 4H), 7.18 (q, *J* = 2.5 Hz, 3H), 6.98 (td, *J* = 9.0, 2.5 Hz, 2H), 6.16 (d, *J* = 9.6 Hz, 2H), 5.51 (d, *J* = 9.5 Hz, 2H), 2.82 (q, *J* = 16.3 Hz, 4H), 2.47 (s, 6H), 1.90–1.74 (m, 2H), 1.30 (s, 6H), 0.98 (s, 6H). ^13^C NMR (101 MHz, CDCl_3_) δ 162.62, 159.13, 147.42, 144.15, 132.14, 131.92, 128.95 (d, *J* = 9.6 Hz), 128.41, 127.55, 125.97, 118.98, 114.02 (d, *J* = 26.8 Hz), 112.74 (d, *J* = 9.5 Hz), 111.85 (d, *J* = 5.5 Hz), 110.78, 104.60 (d, *J* = 23.3 Hz), 62.07, 45.93, 45.36, 26.69, 22.46, 10.64. HRMS (ESI^+^): *m*/*z* = 362.1665, calcd. 362.1665 for C_22_H_21_FN_3_O^+^ [M + H]^+^.

#### (*R*)-*N*-(6-Cyano-2,2-dimethyl-2,3-dihydro-1*H*-inden-1-yl)-5-fluoro-1*H*-benzo[*d*]imidazole-2-carboxamide (**19G**)

^1^H NMR (400 MHz, CDCl_3_) δ 11.59 (d, *J* = 28.1 Hz, 1H), 7.80 (t, *J* = 9.5 Hz, 1H), 7.73 (dd, *J* = 9.0, 4.8 Hz, 1H), 7.62–7.54 (m, 2H), 7.54–7.42 (m, 1H), 7.36 (d, *J* = 7.7 Hz, 1H), 7.31–7.23 (m, 1H), 7.14 (dtd, *J* = 21.0, 9.2, 2.4 Hz, 1H), 5.50 (dd, *J* = 10.0, 2.6 Hz, 1H), 2.93 (q, *J* = 16.4 Hz, 2H), 1.68 (s, 1H), 1.39 (d, *J* = 2.0 Hz, 3H), 1.09 (s, 3H). ^13^C NMR (101 MHz, CDCl_3_) δ 159.46, 147.71, 145.27 (d, *J* = 69.3 Hz), 143.13, 139.38, 132.40, 130.77, 127.96, 126.08, 121.80 (d, *J* = 10.4 Hz), 118.83, 114.49, 110.89, 106.09 (d, *J* = 24.1 Hz), 98.53 (d, *J* = 27.5 Hz), 62.29, 45.85 (d, *J* = 19.7 Hz), 27.08, 22.37. HRMS (ESI^+^): *m*/*z* = 349.1459, calcd. 362.1665 for C_20_H_18_FN_4_O^+^ [M + H]^+^.

#### (*R*)-*N*-(6-Cyano-2,2-dimethyl-2,3-dihydro-1*H*-inden-1-yl)-5-fluorobenzo[*b*]thiophene-2-carboxamide (**19H**)

^1^H NMR (400 MHz, CDCl_3_) δ 7.88–7.77 (m, 2H), 7.55–7.49 (m, 3H), 7.32 (d, *J* = 8.3 Hz, 2H), 7.22 (td, *J* = 8.8, 2.5 Hz, 1H), 6.28 (d, *J* = 9.5 Hz, 1H), 5.50 (d, *J* = 9.6 Hz, 1H), 2.88 (q, *J* = 16.4 Hz, 2H), 1.36 (s, 3H), 1.04 (s, 3H). ^13^C NMR (101 MHz, CDCl_3_) δ 162.19, 159.82, 147.73, 143.72, 140.18, 136.36, 132.20, 127.76, 126.02, 125.06 (d, *J* = 4.6 Hz), 124.09 (d, *J* = 9.3 Hz), 118.96, 115.83 (d, *J* = 25.5 Hz), 110.70, 110.31 (d, *J* = 22.9 Hz), 62.36, 45.95, 45.59, 29.70, 26.90, 22.32. HRMS (ESI^+^): *m*/*z* = 365.1118, calcd. 365.1115 for C_21_H_18_FN_2_OS^+^ [M + H]^+^.

#### (*R*)-5-Fluoro-*N*-(6-(2-fluoroethoxy)-2,2-dimethyl-2,3-dihydro-1*H*-inden-1-yl)-1*H*-indole-2-carboxamide (**21A**)

^1^H NMR (400 MHz, CDCl_3_) δ 9.58 (s, 1H), 7.41 (dd, *J* = 8.9, 4.4 Hz, 1H), 7.28–7.26 (m, 1H), 7.26 (s, 1H), 7.13 (d, *J* = 8.0 Hz, 1H), 7.11–7.02 (m, 1H), 6.87–6.78 (m, 3H), 6.24 (d, *J* = 9.8 Hz, 1H), 5.45 (d, *J* = 9.8 Hz, 1H), 4.85–4.57 (m, 2H), 4.25–4.08 (m, 2H), 2.77 (q, *J* = 15.3 Hz, 2H), 1.33 (s, 3H), 1.05 (s, 3H). ^13^C NMR (101 MHz, CDCl_3_) δ 161.57, 159.38, 157.95, 143.71, 134.71, 133.17, 132.01, 127.73 (d, *J* = 10.3 Hz), 125.96, 114.80, 113.63 (d, *J* = 26.8 Hz), 113.06 (d, *J* = 9.6 Hz), 110.31, 106.14 (d, *J* = 23.4 Hz), 101.91 (d, *J* = 5.1 Hz), 82.77, 81.08, 67.42 (d, *J* = 20.4 Hz), 62.71, 45.35 (d, *J* = 58.4 Hz), 27.42, 22.65. HRMS (ESI^+^): *m*/*z* = 385.1769, calcd. 385.1772 for C_22_H_23_F_2_N_2_O_2_^+^ [M + H]^+^.

#### (*R*)-5-Fluoro-*N*-(6-(3-fluoropropoxy)-2,2-dimethyl-2,3-dihydro-1*H*-inden-1-yl)-1*H*-indole-2-carboxamide (**21B**)

^1^H NMR (400 MHz, CDCl_3_) δ 9.60 (s, 1H), 7.34 (dd, *J* = 8.9, 4.4 Hz, 1H), 7.19 (s, 1H), 7.05 (d, *J* = 8.1 Hz, 1H), 6.98 (td, *J* = 9.1, 2.5 Hz, 1H), 6.83–6.64 (m, 1H), 6.18 (d, *J* = 9.7 Hz, 1H), 5.38 (d, *J* = 9.8 Hz, 1H), 4.54 (dt, *J* = 47.1, 5.8 Hz, 2H), 3.97 (td, *J* = 6.1, 1.6 Hz, 2H), 2.69 (q, *J* = 15.3 Hz, 2H), 2.19–1.90 (m, 2H), 1.26 (s, 3H), 0.98 (s, 3H). ^13^C NMR (101 MHz, CDCl_3_) δ 161.6, 159.4, 158.0, 143.7, 134.7, 133.2, 132.0, 127.7 (d, *J* = 10.3 Hz), 125.9, 114.8, 113.6 (d, *J* = 26.8 Hz), 113.0 (d, *J* = 9.6 Hz), 110.3, 106.14 (d, *J* = 23.4 Hz), 101.91 (d, *J* = 5.1 Hz), 82.7, 81.1, 67.1 (d, *J* = 20.4 Hz), 62.7, 54.8, 45.0 (d, *J* = 58.4 Hz), 27.42, 22.65. HRMS (ESI^+^): *m*/*z* = 399.1877, calcd. 399.1879 for C_23_H_25_F_2_N_2_O_2_^+^ [M + H]^+^.

#### (*R*)-*N*-(6-Carbamoyl-2,2-dimethyl-2,3-dihydro-1*H*-inden-1-yl)-5-fluoro-1*H*-indole-2-carboxamide (**21C**)

^1^H NMR (400 MHz, DMSO-*d*_6_) δ 11.73 (s, 1H), 7.93 (s, 1H), 7.83–7.74 (m, 2H), 7.45 (dd, *J* = 8.9, 4.7 Hz, 1H), 7.39 (dd, *J* = 9.8, 2.5 Hz, 1H), 7.36–7.30 (m, 2H), 7.23 (s, 1H), 7.06 (td, *J* = 9.3, 2.6 Hz, 1H), 5.39 (d, *J* = 9.3 Hz, 1H), 3.32 (s, 3H), 2.83 (q, *J* = 16.0 Hz, 2H), 1.23 (s, 3H), 0.98 (s, 3H). ^13^C NMR (101 MHz, CDCl_3_) δ 173.14, 166.28, 163.48, 161.17, 151.12, 148.10, 138.41, 138.38, 137.87, 129.73, 128.90, 118.72, 118.63, 117.29 (d, *J* = 26.6 Hz), 110.88 (d, *J* = 21.8 Hz), 108.72, 66.75, 50.41, 50.17, 32.90, 27.99.

HRMS (ESI^+^): *m*/*z* = 366.1616, calcd. 366.1612 for C_21_H_21_FN_3_O_2_^+^ [M + H]^+^.

#### *N*-(Adamantan-1-yl)-5-fluoro-1*H*-indole-2-carboxamide (**21E**)

^1^H NMR (300 MHz, cdcl_3_) δ 7.79–7.64 (m, 2H), 7.53–7.34 (m, 4H), 5.81 (s, 1H), 2.13 (s, 8H), 1.72 (s, 6H). ^13^C NMR (75 MHz, cdcl_3_) δ 166.65, 136.02, 133.21, 130.99, 130.05, 128.37, 128.30 (d, *J* = 6.9 Hz), 126.68, 52.28, 41.67, 36.37, 29.49. HRMS (ESI^+^): *m*/*z* = 313.3957, calcd. 313.3959 for C_19_H_21_N_2_O^+^ [M + H]^+^.

#### (*R*)-5-Bromo-*N*-(6-cyano-2,2-dimethyl-2,3-dihydro-1*H*-inden-1-yl)-1*H*-indole-2-carboxamide (**23**)

^1^H NMR (400 MHz, CDCl_3_) δ 10.08 (s, 1H), 7.77 (d, *J* = 0.7 Hz, 1H), 7.53 (d, *J* = 9.8 Hz, 2H), 7.46–7.21 (m, 3H), 6.91 (s, 1H), 5.51 (d, *J* = 5.9 Hz, 1H), 2.88 (q, *J* = 16.4 Hz, 2H), 1.83 (s, 3H), 1.35 (s, 3H). ^13^C NMR (101 MHz, CDCl_3_) δ 171.38, 161.70, 147.84, 143.76, 135.07, 132.14, 127.80, 127.69, 126.00, 124.25 (d, *J* = 2.4 Hz), 118.98, 113.81, 113.56, 110.50, 102.08, 61.86, 60.47, 26.92, 22.24, 21.01, 14.14. HRMS (ESI^+^): *m*/*z* = 408.0703, calcd. 408.0706 for C_21_H_19_^79^BrN_3_O^+^ [M + H]^+^.

#### (*R*)-*N*-(6-Cyano-2,2-dimethyl-2,3-dihydro-1*H*-inden-1-yl)-5-(4,4,5,5-tetramethyl-1,3,2-dioxaborolan-2-yl)-1*H*-indole-2-carboxamide (**24**)

Compound **23** (200 mg, 0.5 mmol, 1 equiv), 4,4,4′,4′,5,5,5′,5′-octamethyl-2,2′-bis(1,3,2- dioxaborolane) (124 mg, 0.5 mmol, 1 equiv), potassium acetate (196 mg, 1 mmol, 2 equiv), and [PdCl_2_(dppf)] (36 mg, 0.05 mmol, 0.1 equiv) were placed in a 50 mL round-bottom flask and secured three times. 1,4-Dioxane (12 mL) was added under argon countercurrent and the mixture was secured three times. The reaction mixture was then refluxed for 6 h. The cooled reaction solution was filtered through celite, the filter residue was washed with 50 mL of 1,4-dioxane, and the filtrate was concentrated to dryness under reduced pressure. The resulting residue was purified by flash chromatography (SiO_2_, EA/Hex, 1:4) to give **24** as a white solid in 88% yield. ^1^H NMR (300 MHz, cdcl_3_) δ 9.48 (s, 1H), 8.18 (d, *J* = 0.8 Hz, 1H), 7.75 (dd, *J* = 8.3, 1.1 Hz, 1H), 7.58–7.49 (m, 1H), 7.46 (d, *J* = 8.3 Hz, 1H), 7.36–7.28 (m, 1H), 6.29 (d, *J* = 9.7 Hz, 1H), 5.54 (d, *J* = 9.8 Hz, 1H), 2.89 (d, *J* = 7.1 Hz, 1H), 1.37 (s, 8H), 1.36 (s, 1H), 1.05 (s, 2H). HRMS (ESI^+^): *m*/*z* = 456.2455, calcd. 456.2453 for C_27_H_31_BN_3_O3^+^ [M + H]^+^.

### Radiochemistry

#### Automated Radiosynthesis of [^18^F]RM365

Remote controlled radiosynthesis was performed using a Synchrom R&D EVO III automated synthesizer (Elysia-Raytest, Straubenhardt, Germany). Briefly, [^18^F]fluoride (8–12 GBq) was trapped on a Waters QMA cartridge and eluted with a solution containing 100 μL of TBAHCO_3_ and 30 μL of K_2_CO_3_ dissolved in a mixture of H_2_O/MeCN (1 mL, 1:4, *v/v*) into the reaction vessel and dried via azeotropic distillation. A further 1.5 mL of dried MeCN was added. After complete dryness, a solution of 13.2 μmol of Cu(Py)_4_(OTf)_2_ in 450 μL of DMA/*t-*BuOH (2:1, *v/v*) was added, followed by a solution of 6.6 μmol of boronic acid pinacol ester **24** in 450 μL of DMA/*t*-BuOH (2:1, *v/v*), and the reaction mixture was stirred at 120 °C for 10 min. Upon cooling to 40 °C, the reaction mixture was diluted with H_2_O/MeCN (4 mL, 1:4, *v/v*), and the solution was transferred to the semipreparative HPLC. [^18^F]RM365 was collected in the HPLC collection vial containing 40 mL of H_2_O and trapped in the Sep-Pak C18 light cartridge. The cartridge was washed with 2 mL of H_2_O and [^18^F]RM365 eluted with 1.3 mL of EtOH. This ethanolic solution was transferred outside of the shielded cell, the solvent was evaporated at 70 °C in a gentle stream of nitrogen for 5–10 min, and [^18^F]RM365 was reconstituted in an isotonic saline solution for further biological characterization. The total synthesis time was about 75 min.

#### Quality Control

Radio-TLC was performed on Alugram SIL G/UV_254_ precoated plates (Macherey-Nagel; Düren, Germany) with PE/EA (1:1, *v*/*v*). The plates were exposed to storage phosphor screens (BAS IP MS 2025 E, GE Healthcare Europe GmbH, Freiburg, Germany) and recorded using an Amersham Typhoon RGB Biomolecular Imager (GE Healthcare Life Sciences). Images were quantified with ImageQuant TL8.1 software (GE Healthcare Life Sciences).

Analytical chromatographic separations were performed on a JASCO LC-2000 system, incorporating a PU-2080*Plus* pump, an AS-2055*Plus* auto injector (100 μL sample loop), and a UV-2070*Plus* detector coupled with a γ-detector (GABI Star; raytest Isotopenmessgeräte GmbH, Straubenhardt, Germany). Data analysis was performed with Galaxie chromatography software (Agilent Technologies, Santa Clara, CA, USA) using chromatograms obtained at 254 nm. Analytical chiral chromatographic measurements of (−)-[^18^F]7 were performed on a JASCO LC-4000 system incorporating a PU-4180-LPG pump, AS-4050 auto injector (100 μL sample loop), and a UV-diode array detector MD-4015 (monitoring at 254 nm) coupled with a circular dichroism chiral detector CD-4095 (monitoring at 285 nm). Data analysis was performed with ChromNAV 2.3C software (JASCO Deutschland GmbH, Pfungstadt, Germany).

Radiochemical yield, radiochemical purity, and analyses of plasma and brain samples were assessed via reversed-phase HPLC (RP-HPLC) in gradient mode (0–5 min: 10% MeCN/20 mM NH_4_OAc_aq._, 5–20 min: 10% → 90% MeCN/20 mM NH_4_OAc_aq._, 20–25 min: 90% MeCN/20 mM NH_4_OAc_aq._, 25–26 min: 90% → 10% MeCN/20 mM NH_4_OAc_aq._ 26–30 min: 10% MeCN/20 mM NH_4_OAc_aq._).

Molar activity was determined using analytical HPLC with a Reprosil-Pur C18-AQ column (250 × 4.6 mm, 5 μm) and 50% MeCN/20 mM NH_4_OAc_aq._ as an eluent at a flow rate of 1 mL·min^–1^ and UV detection at 254 nm.

#### Determination of Lipophilicity (LogD_7.4_)

Log *D*_7.4_ of [^18^F]RM365 was experimentally determined in n-octanol/PBS, 0.01 M, pH 7.4, at room temperature by the shake flask method. The measurement was performed twice in triplicate.^87^

### Biological Experiments

All studies involving animals were carried out according to the national law on the protection of animals and were approved by the responsible authorities (Landesdirektion Sachsen, No. DD24.1-5131/446/19; TVV 18/18).

#### Determination of Binding Affinities by Homogenate Assays

The binding affinities toward hCB1R and hCB2R were determined according to a previously published protocol.^22^ In brief, membrane preparations obtained from CHO cell lines stably transfected with either human CB1R (hCB1R-CHO; obtained from Euroscreen, Gosselies, Belgium) or human CB2R (hCB2R-CHO; obtained from Paul L. Prather, Department of Pharmacology and Toxicology, College of Medicine, University of Arkansas for Medical Sciences, USA) were incubated with [^3^H]SR141716A (1,554 GBq/mmol; PerkinElmer Life and Analytical Sciences, Rodgau, Germany; final concentration ∼2 nM) or [^3^H]WIN55212-2 (6,438 GBq/mmol; PerkinElmer Life and Analytical Sciences, Rodgau, Germany; final concentration ∼3 nM) and the respective test compounds at different concentrations (final concentration 10^–5^–10^–11^ M) diluted from DMSO stock solutions (1% DMSO final concentration) in incubation buffer (50 mM TRIS-HCl, pH 7.4, supplemented with 0.1% bovine serum albumin, 5 mM MgCl_2_, and 1 mM EDTA) at rt for 90 min.

Homologous radioligand displacement studies investigating the potential of RM365 to displace [^18^F]RM365 from binding sites in membrane homogenates of rat spleen or hCB2R-CHO cells were performed according to the same protocol.

The nonspecific binding of the respective radioligand was determined by coincubation with CP55,940 (final concentration 10^–5^ M). The normalized values of bound activity (% specific binding) were calculated and plotted vs the logarithm of the concentration of the respective test compound. The IC_50_ values of the resulting inhibition curves were estimated by nonlinear regression analysis (GraphPad Prism 2.01). To calculate the *K*_i_, the equation of Cheng and Prusoff was used. For homologous competition experiments, *K*_i_ = *K*_D_.^38^

#### In Vitro Autoradiography

The *in vitro* autoradiographic experiments were performed according to a previously published protocol.^88^ In brief, 10 μm cryosections of CD-1 mouse brain rat spleen (female SPRD rat,10–12 weeks) were incubated in binding buffer (50 mM Tris-HCl, pH 7.4, 5% bovine serum albumin (BSA), 5 mM MgCl_2_, 1 mM EDTA) with [^18^F]RM365 alone (total binding) or with coadministered RM365 at 1 μM or 10 nM (self-blocking), or of 1 μM CP55940 (CB1R/CB2R nonselective agonist), SR141716A (CB1R-selective antagonist), or SR144528 (CB2R-selective antagonist) for 1 h at room temperature. Afterward, the samples were washed and exposed to imaging plates (Fuji Photo Film, Co. Ltd., Tokyo, Japan) and eventually scanned using a HD-CR 35 scanner (Raytest Isotopenmessgeraete GmbH, Straubenhardt, Germany). The scan data were visualized and processed by computer-assisted microdensitometry (Aida version 2.31, Raytest Isotopenmessgeräte GmbH, Straubenhardt, Germany).

#### Quantification of Radiometabolites

About 30 MBq of [^18^F]RM365 dissolved in about 150 μL of isotonic saline was administered intravenously as a bolus in the tail vein of awake female CD-1 mice weighing about 33 g (*n* = 3). At 30 min p.i., the animals were anesthetized and blood was withdrawn by retrobulbar bleeding using glass capillaries. Immediately afterward, the animals were euthanized by cervical dislocation and released urine sampled. Blood plasma was obtained from the whole blood sample by centrifugation (2 min, 8000 rpm, room temperature). In addition, the brain was isolated and homogenized in 1 mL of demineralized water on ice (1000 rpm, 10 strokes; glass vessel, PTFE plunger; Potter S, B. Braun Biotech International, Goettingen, Germany).

The samples were further processed for subsequent radio-chromatographic analyses. Two consecutive extractions were performed in duplicate for plasma and brain determinations. Plasma and brain samples were added to an ice-cold MeOH/H_2_O mixture (9:1, *v*/*v*). The samples were vortexed for 3 min, incubated on ice for 5 min, and centrifuged at 10,000 rpm for 5 min. Supernatants were collected, the precipitates were redissolved in 100 μL of extraction solvent, and the extraction procedure was repeated. The activities of supernatants and precipitates were measured in an γ-counter (1480 WIZARD, Fa. PerkinElmer), and the extraction efficiencies were calculated as the ratio of the radioactivity in the supernatant to the radioactivity in the original sample (supernatant + precipitate). The supernatants from both extractions were combined, concentrated at 70 °C under an argon stream up to a remaining volume of 100 μL, and subsequently analyzed by analytical radio-HPLC with a gradient system as applied in Section 5.2.2.

#### PET Imaging

*In vivo* biodistribution studies of [^18^F]RM365 were performed in female CD-1 mice (*n* = 4, 31 ± 3 g) and female Wistar rats (*n* = 4, 150 ± 11 g). For the uptake studies into the brain, male Wistar rats carrying the stereotactically injected AAV2/7-CaMKII0.4-intron-hCB2R(D80N) (right striatum) and AAV2/7-CaMKII0.4-intron-3flag-eGFP (control/contralateral, left striatum) were used (*n* = 6; bodyweights were 260 ± 11 g). Data were assessed by dynamic small animal PET (Nanoscan, Mediso, Budapest, Hungary) in 60 min recordings, followed by T1-weighted (GRE, TR/TE = 15.0/2.4 ms, 252/252, FA = 25°) MR imaging with whole-body coils for anatomical correlation and attenuation correction.

The PET scans were performed 3−4 months after the stereotactic injections. Animals were initially anesthetized with 5% isoflurane and placed on a thermostatically heated animal bed, where anesthesia was maintained with 2% isoflurane in 60% oxygen/38% room air. Biodistribution studies were performed as baseline scans, whereas the rats with the striatal hCB2R (D80N) were pretreated either by i.v. injections of vehicle solution only, containing DMSO: Kolliphor EL: saline in a composition of 1:2:7 (control group, 3 months after AAV2/7 injection) or 5 mg/kg GW405833 for the displacement experiments 20 min after [^18^F]RM365 administration (4 months after AAV2/7 injection). [^18^F]RM365 (biodistribution in mice 5.1 ± 1 MBq, 161 ± 58 pmol; in rats, 26 ± 3 MBq, 344 ± 133 pmol; in rats with striatal hCB2R(D80N) overexpression 24 ± 4MBq, 251 ± 72 pmol) was injected into the lateral tail vein (bolus within 5 s) at the start of the PET acquisition. List-mode PET data were binned as a series of attenuation-corrected sinogram frames (12 × 10 s, 6 × 30 s, 5 × 60 s, and 10 × 300 s) and were reconstructed by ordered subset expectation maximization (OSEM3D) with four iterations, six subsets, and a voxel size of 0.4 mm^3^ (Nucline v2.01, Mediso, Hungary). The analysis of reconstructed data sets was performed with PMOD software (v4.205, PMOD Technologies LLC, Zurich, Switzerland). Nonparametric (semiquantitative) analysis of achieved time–activity curves (TACs) was performed with Microsoft Excel to determine the time to peak, the TAC peak value, and the area under the curve (AUC)
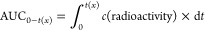
where *c* (radioactivity) is expressed as the standardized uptake value normalized to the bodyweight in g (SUV). Data are shown in mean ± standard deviation (SD). Group differences were tested by Student’s *t*-test, whereas the TACs of reference tissues with and without displacement by GW405833 were tested by a two-way ANOVA, and additionally, the target region by Bonferroni’s multiple comparison test, with *p* < 0.05 designated as significant. Area under the curves (AUCs) and corresponding 95% confidence intervals (CIs, 95%) were calculated with GraphPad Prism (v.8.2) following the assumptions described by Gagnon et al.^39^

## Supporting Information Available

NMR characterization of compounds **6**, **19A–H**, and **21A–E**, **23** and **24**; HPLC analysis of compounds **6**, **19A–H**, and **21A–E**; analytical UV and OR chromatograms of (+)-**6** and (−)-**6** (Figure S1); in vitro autoradiography of [18F]RM365 (Figure S2); computational chemistry (Figures S3–S6); PET imaging (Figures S7–S11) (PDF)
Molecular Formula Strings (XLS)
PDB_Docking (ZIP)

## Abbreviations Used

BOP: (benzotriazol-1-yloxy)tris(dimethylamino)phosphonium hexafluorophosphate
BBB: blood–brain barrier
CB1R: cannabinoid receptor type 1
CB2R: cannabinoid receptor type 2
K2.2.2: 2.2.2-cryptand
LPS: lipopolysaccharide
DMF: *N*,*N*-dimethylformamide
HPLC: high-performance liquid chromatography
PET: positron emission tomography
RCC: radiochemical conversion
SUV: standardized uptake value
SUVr: SUV ratio x-to-reference
SAR: structure–activity relationship
TBAF: tetrabutylammonium fluoride
THC: (−)-*trans*-Δ^9^-tetrahydrocannabinol
Et_3_N: triethylamine
