# Supplementary material for: Synthesis, Structure–Activity Relationships, Radiofluorination, and Biological Evaluation of [18F]RM365, a Novel Radioligand for Imaging the Human Cannabinoid Receptor Type 2 (CB2R) in the Brain with PET
Source: J Med Chem. 2023 Oct 10;66(20):13991–4010. doi: 10.1021/acs.jmedchem.3c01035 (PMC10614203; doi:10.1021/acs.jmedchem.3c01035)
Supplement: Supplementary file 1 — jm3c01035_si_001.pdf [file jm3c01035_si_001.pdf]

Supporting Information

Synthesis, Structure Activity Relationship, Radiofluorination and Biological Evaluation of [^{18}F]RM365, a Novel Radioligand for Imaging the Human Cannabinoid Receptor Type 2 (CB2R) in the Brain with PET

Rodrigo Teodoro [1], Daniel Gündel [1], Winnie Deuther-Conrad [1], Aleksandr Kazimir [2], Magali Toussaint [1], Barbara Wenzel [1], Guy Bormans [3], Evamarie Hey-Hawkins [2], Klaus Kopka [1,4], Peter Brust [1,5] and Rareş-Petru Moldovan [1,]*

1 Helmholtz-Zentrum Dresden-Rossendorf (HZDR), Institute of Radiopharmaceutical Cancer Research, Department of Neuroradiopharmaceuticals, Research site Leipzig, 04318 Leipzig, Germany

2 Universität Leipzig, Faculty of Chemistry and Mineralogy, Institute of Inorganic Chemistry, Johannisallee 29, 04103 Leipzig, Germany

3 Radiopharmaceutical Research, Department of Pharmaceutical and Pharmacological Sciences, KU Leuven, BE-3000 Leuven, Belgium

4 Faculty of Chemistry and Food Chemistry, School of Science, TU Dresden, 01069 Dresden, Germany

5 The Lübeck Institute of Experimental Dermatology, University Medical Center Schleswig-Holstein, 23562 Lübeck, Germany

* Correspondence: Rareş-Petru Moldovan (r.moldovan@hzdr.de, Tel.: +49-3412341794634)

Content:

- NMR characterization of compounds **6**, **19A**-**H** and **21A**-**E**, **23** and **24**
- HPLC analysis of compounds **6**, **19A**-**H** and **21A**-**E**
- Analytical UV and OR chromatograms of (+)-**6** and (–)-**6** (Figure S1)
- In vitro autoradiography of [^{18}F]RM365 (Figure S2)
- Computational Chemistry (Figure S3-S6)
- PET Imaging (Figure S7-S11)

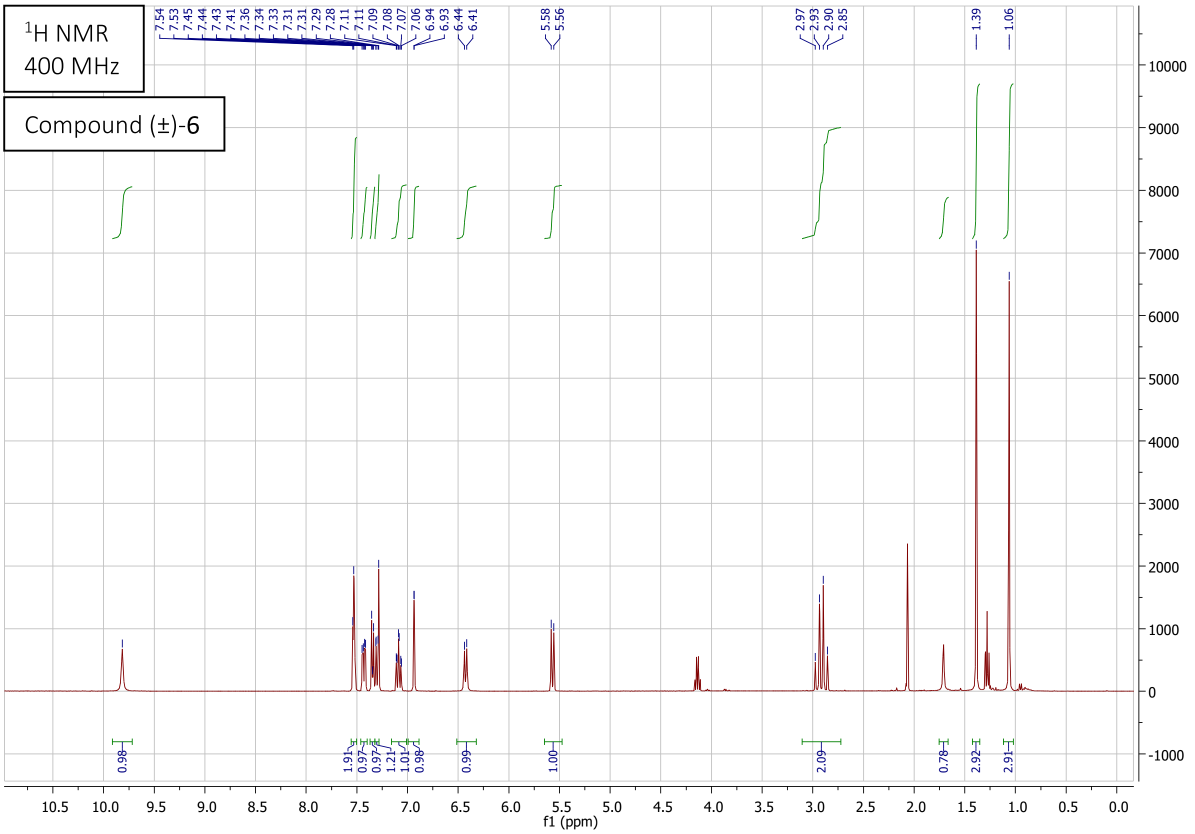
1H NMR
400 MHz
Compound (±)-6
7.54 7.53 7.45 7.44 7.43 7.41 7.36 7.34 7.33 7.31 7.31 7.29 7.28 7.11 7.11 7.09 7.08 7.07 7.06 6.94 6.93 6.44 6.41
5.58 5.56
2.97 2.93 2.90 2.85
1.39
1.06
0.98 1.91 0.97 0.97 1.21 1.01 0.98 0.99 1.00 2.09 0.78 2.92 2.91
f1 (ppm)

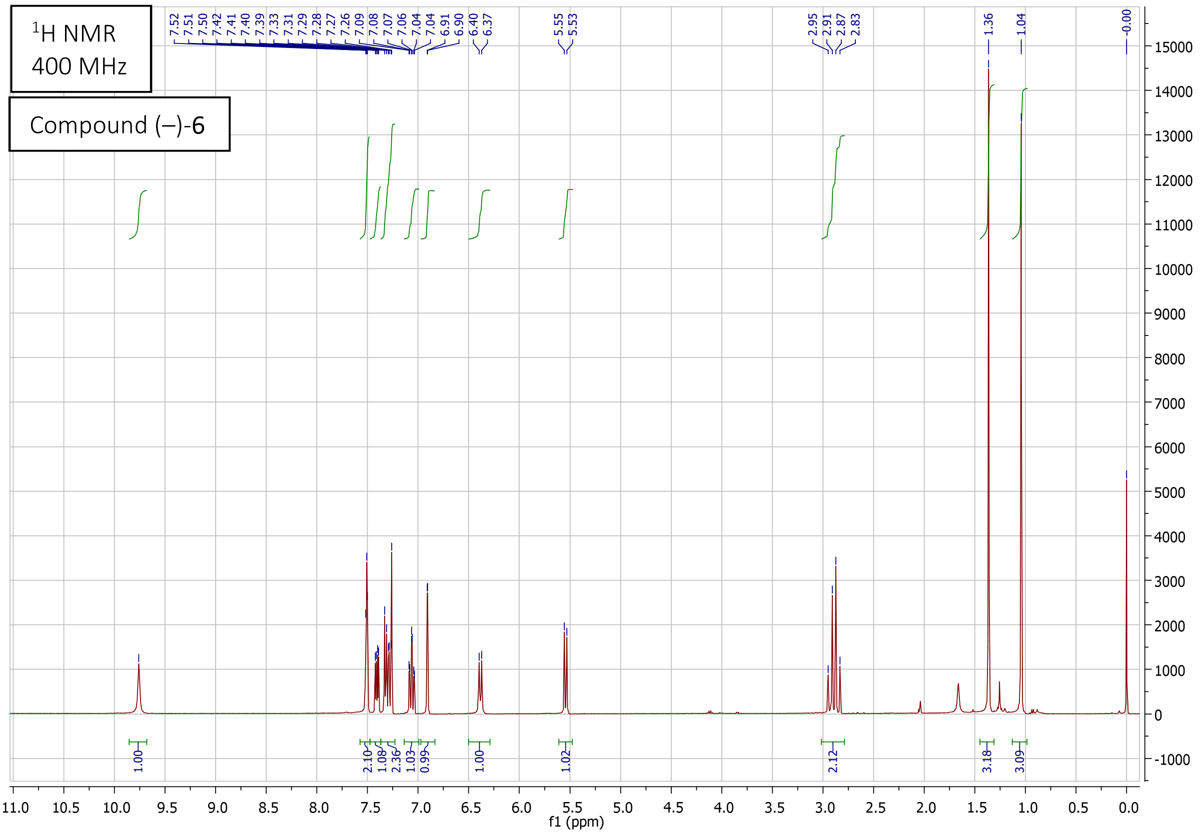
1H NMR
400 MHz
Compound (–)-6
7.52 7.51 7.50 7.42 7.41 7.40 7.39 7.33 7.31 7.29 7.28 7.27 7.26 7.09 7.08 7.07 7.06 7.04 7.04 6.91 6.90 6.40 6.37
5.55 5.53
2.95 2.91 2.87 2.83
1.36
1.04
-0.00
1.00 2.10 1.08 2.36 1.03 0.99 1.00 1.02 2.12 3.18 3.09
f1 (ppm)

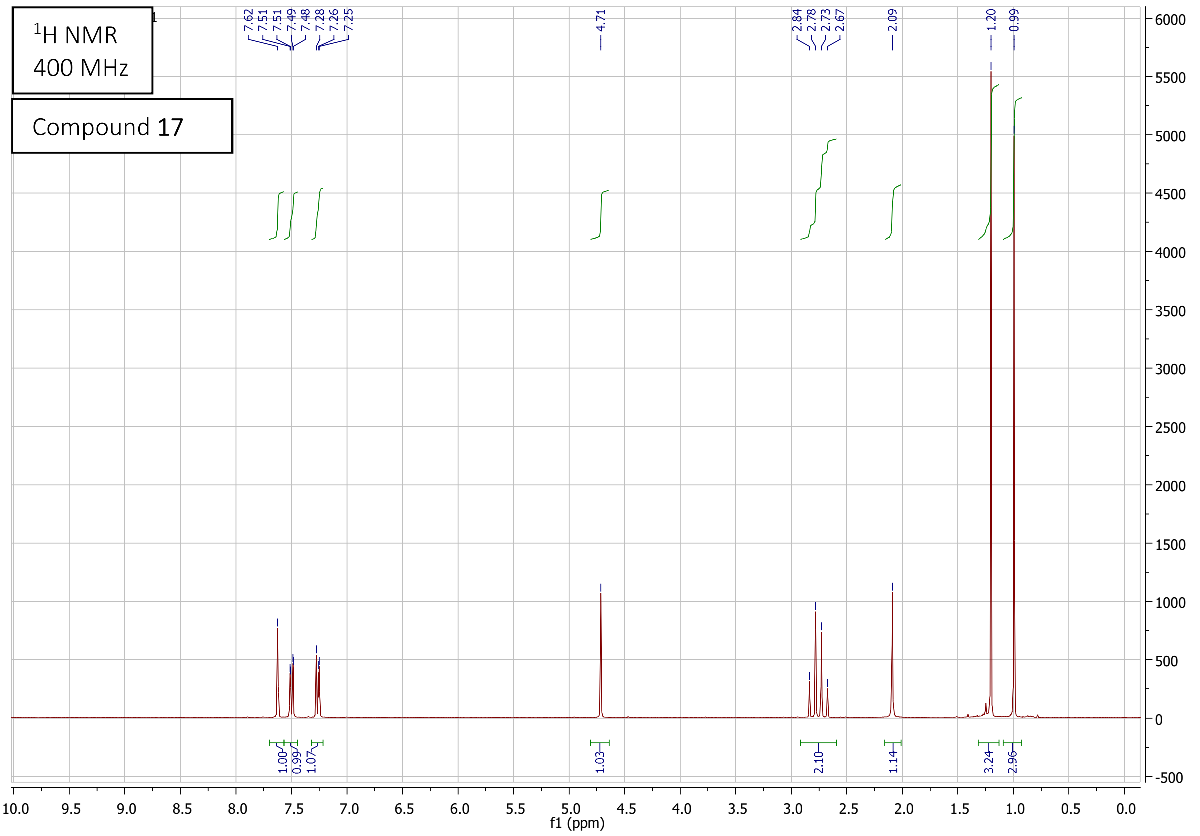

^{1}H NMR
400 MHz
Compound 17
f1 (ppm)

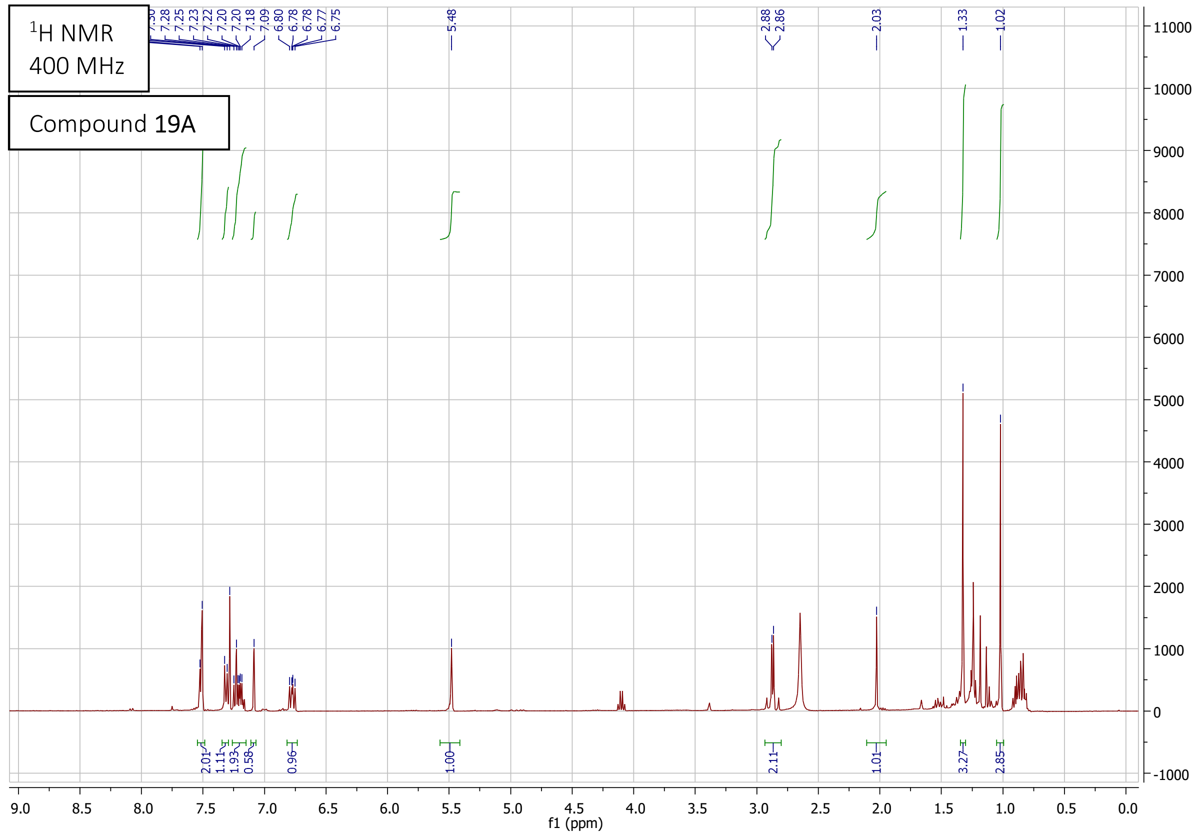

^{1}H NMR
400 MHz
Compound 19A
f1 (ppm)

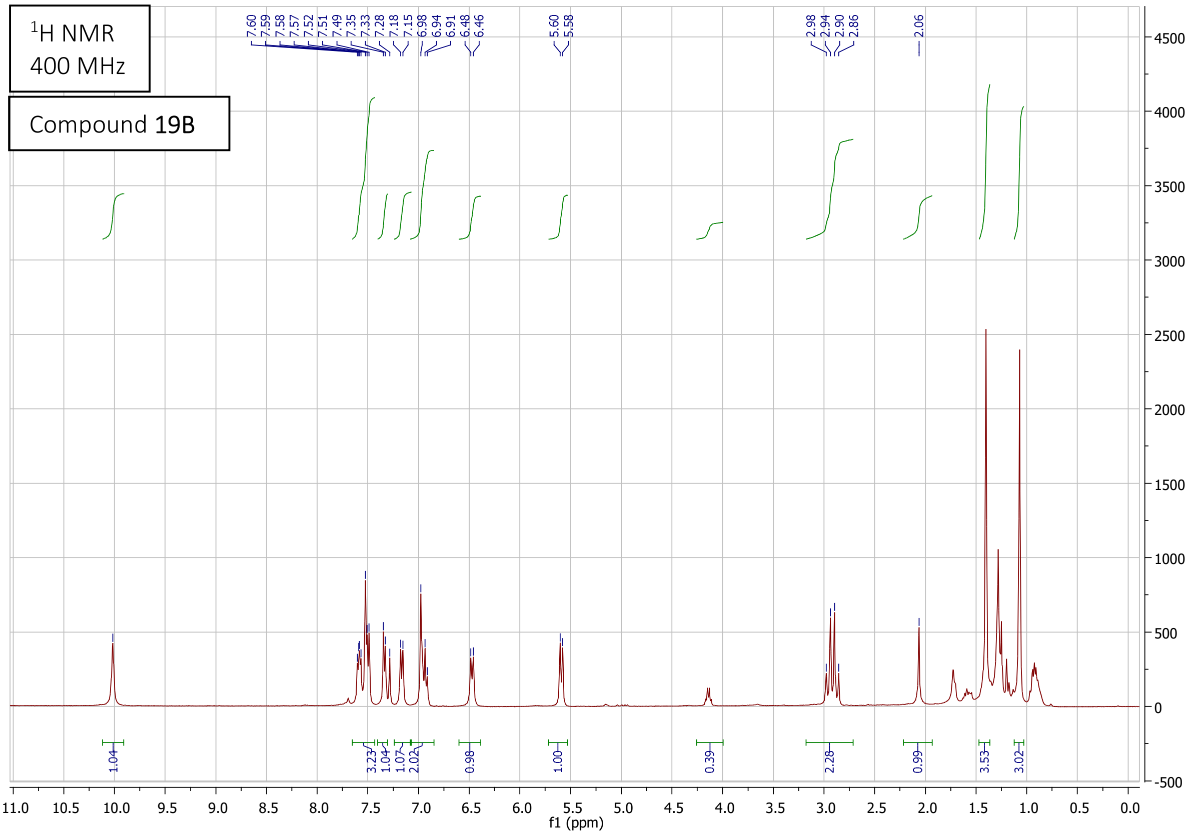

1H NMR
400 MHz
Compound 19B
f1 (ppm)

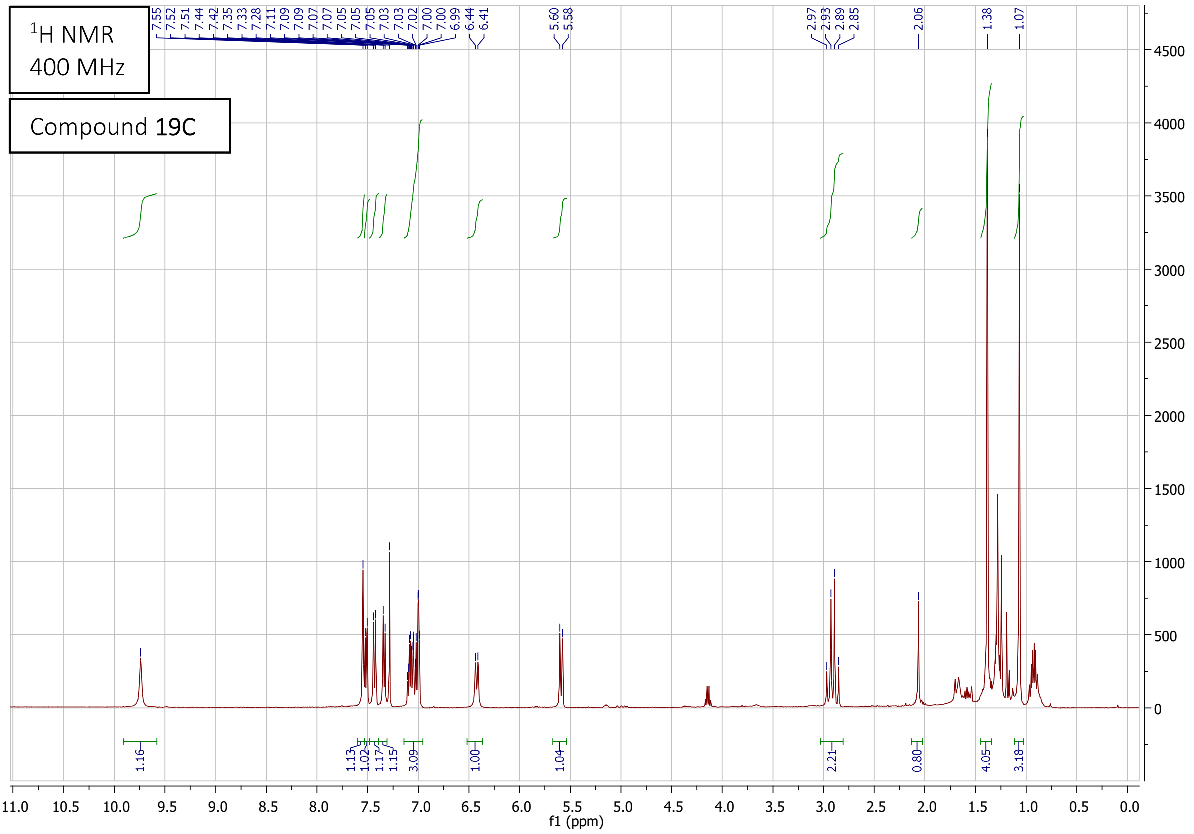

1H NMR
400 MHz
Compound 19C
f1 (ppm)

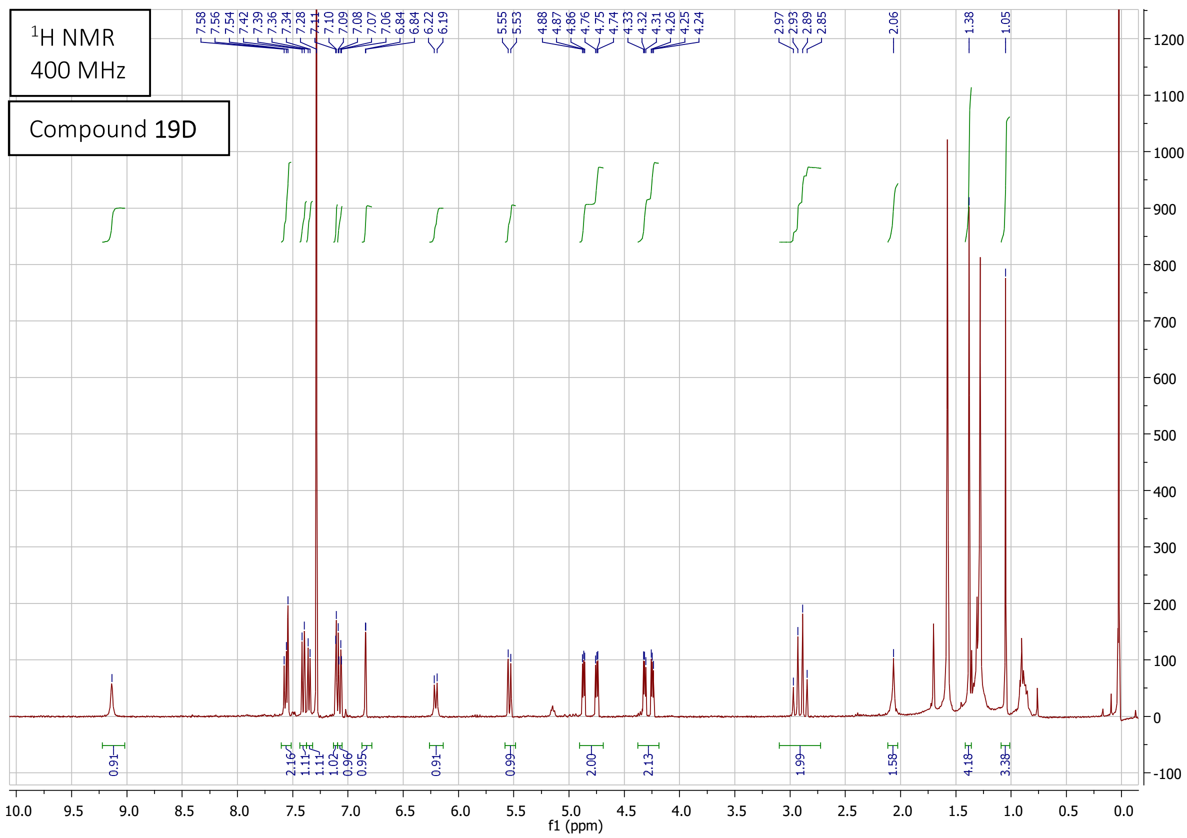
^{1}H NMR
400 MHz
Compound 19D
f1 (ppm)

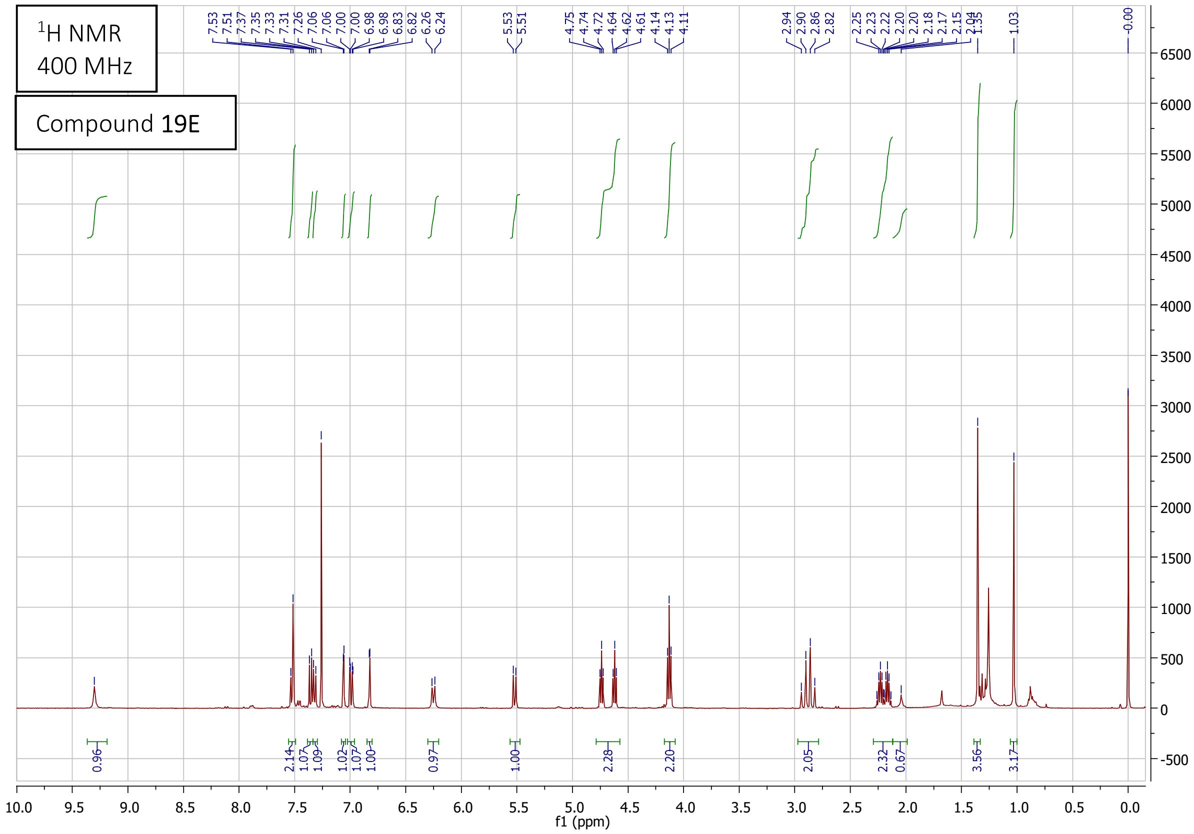
^{1}H NMR
400 MHz
Compound 19E
f1 (ppm)

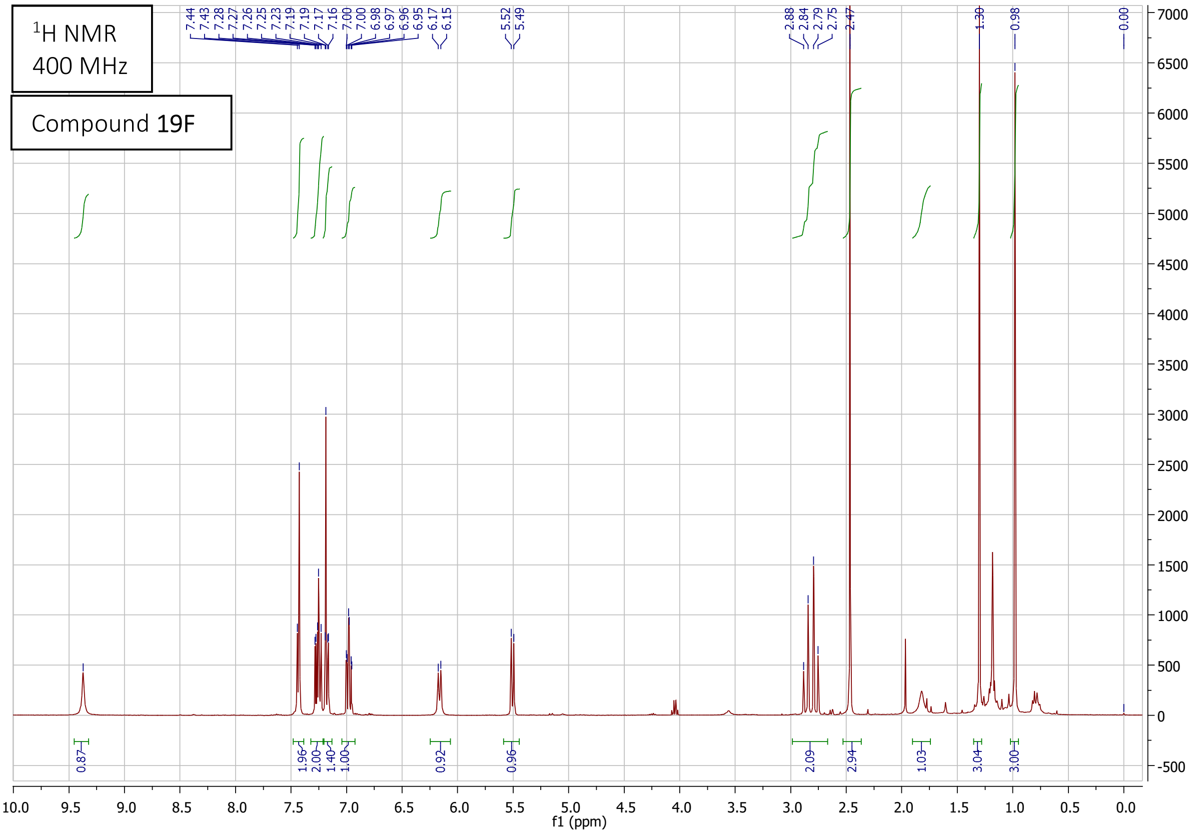

^{1}H NMR
400 MHz
Compound 19F
f1 (ppm)

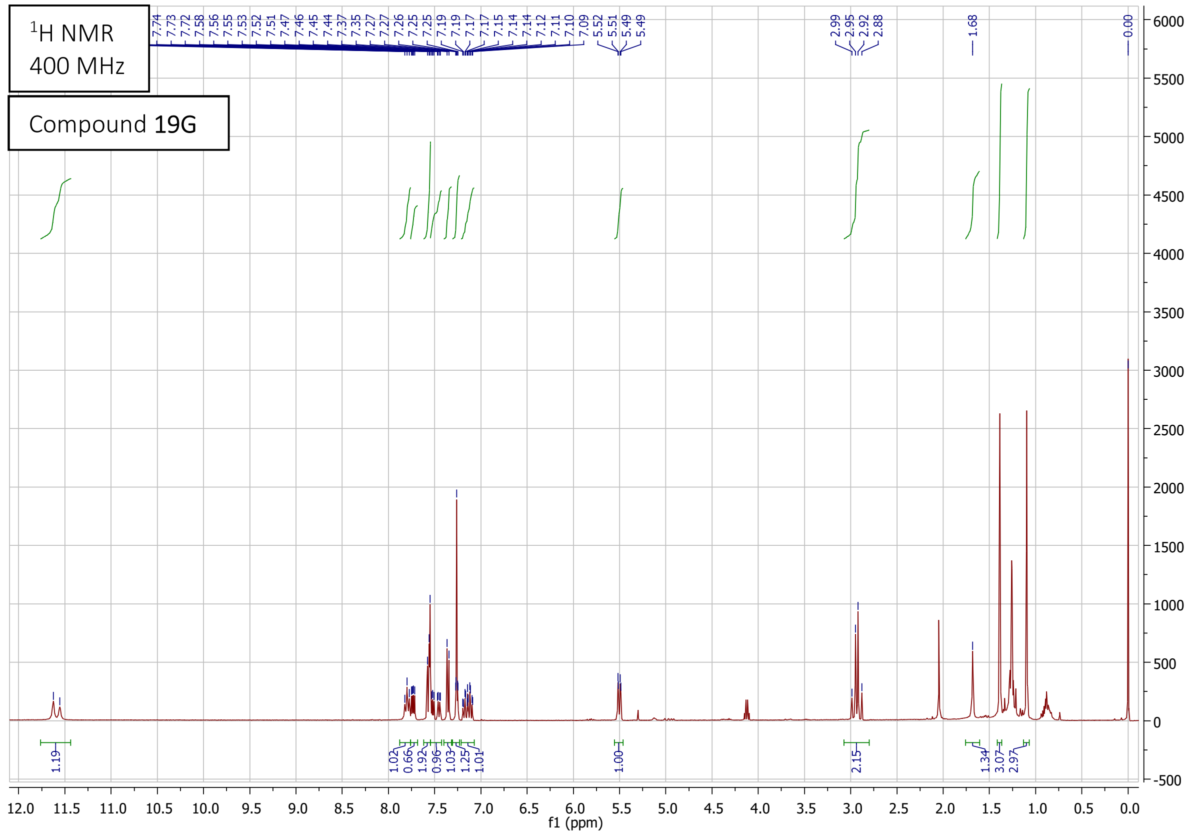

^{1}H NMR
400 MHz
Compound 19G
f1 (ppm)

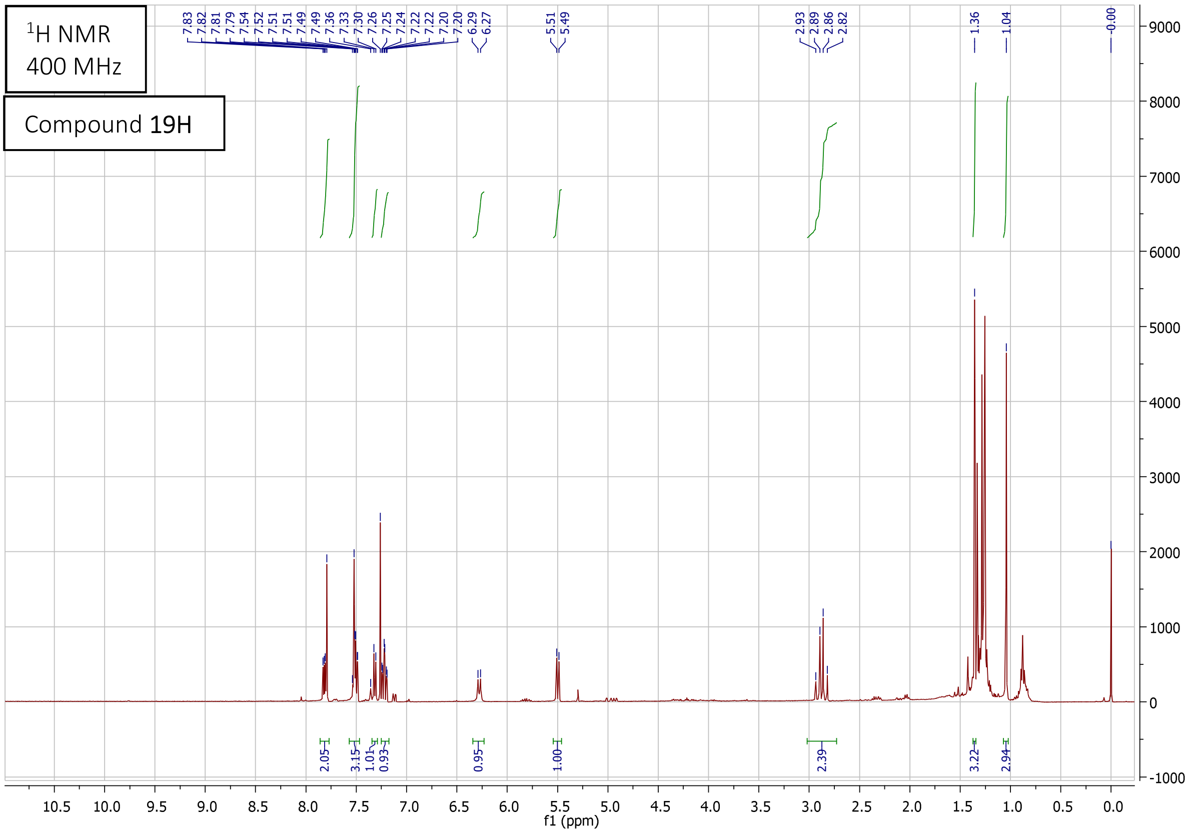

1H NMR
400 MHz
Compound 19H
7.83
7.82
7.81
7.79
7.54
7.52
7.51
7.51
7.49
7.49
7.36
7.33
7.30
7.26
7.25
7.24
7.22
7.22
7.20
7.20
6.29
6.27
5.51
5.49
2.93
2.89
2.86
2.82
1.36
1.04
0.00
2.05
3.15
1.01
0.93
0.95
1.00
2.39
3.22
2.94
f1 (ppm)

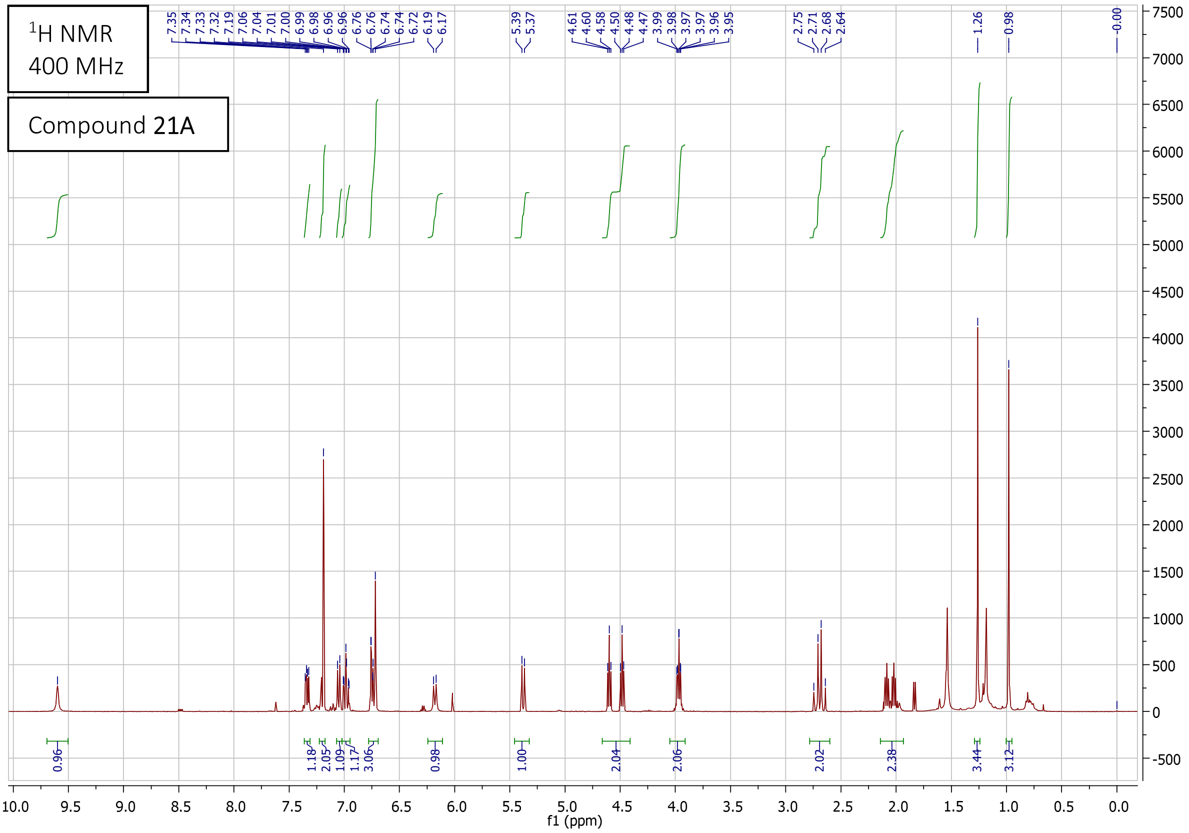

^{1}H NMR
400 MHz
Compound 21A
f1 (ppm)

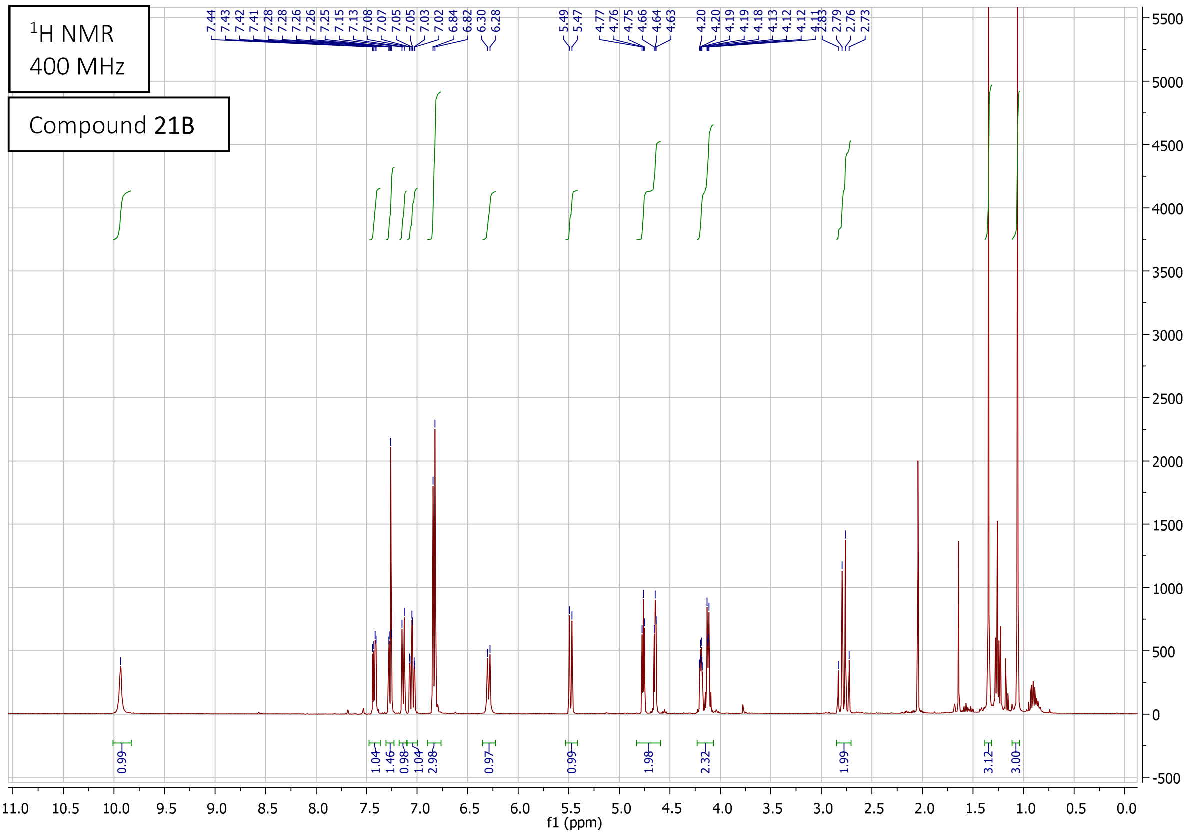

^{1}H NMR
400 MHz
Compound 21B
f1 (ppm)

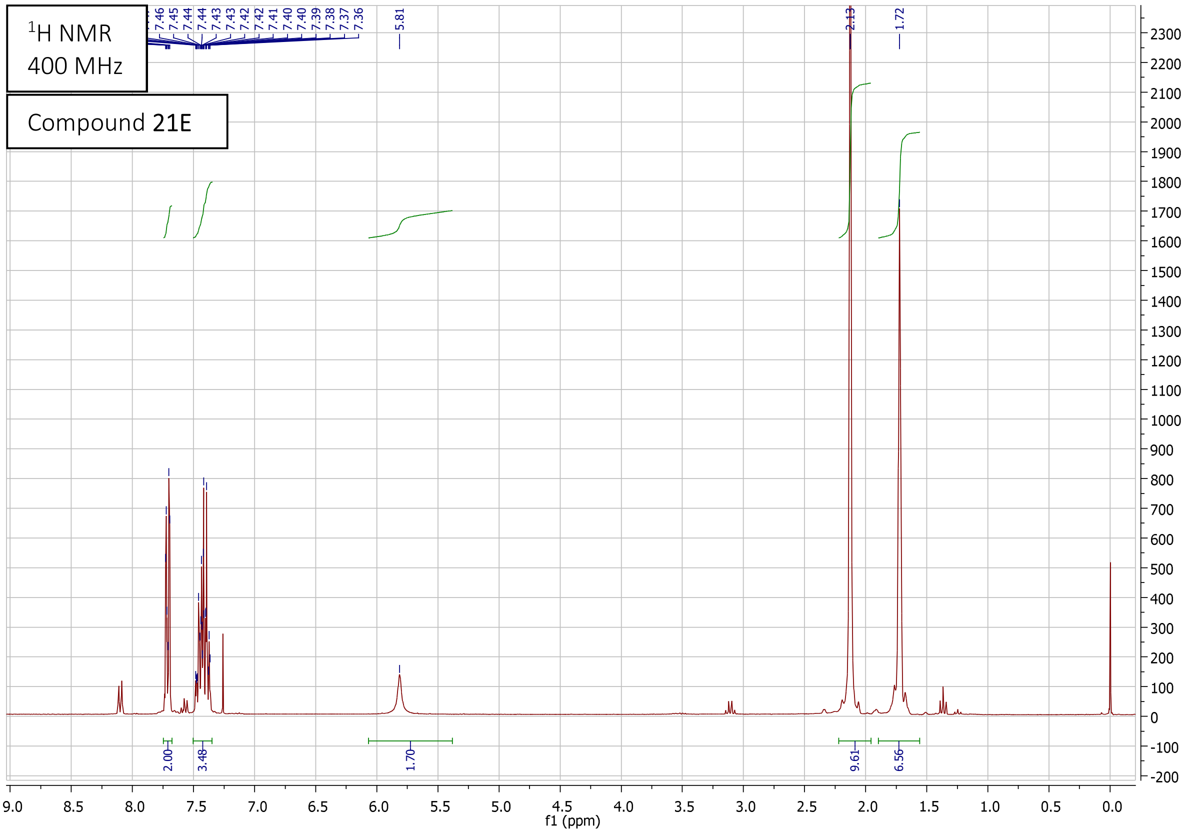

^{1}H NMR
400 MHz
Compound 21E
f1 (ppm)

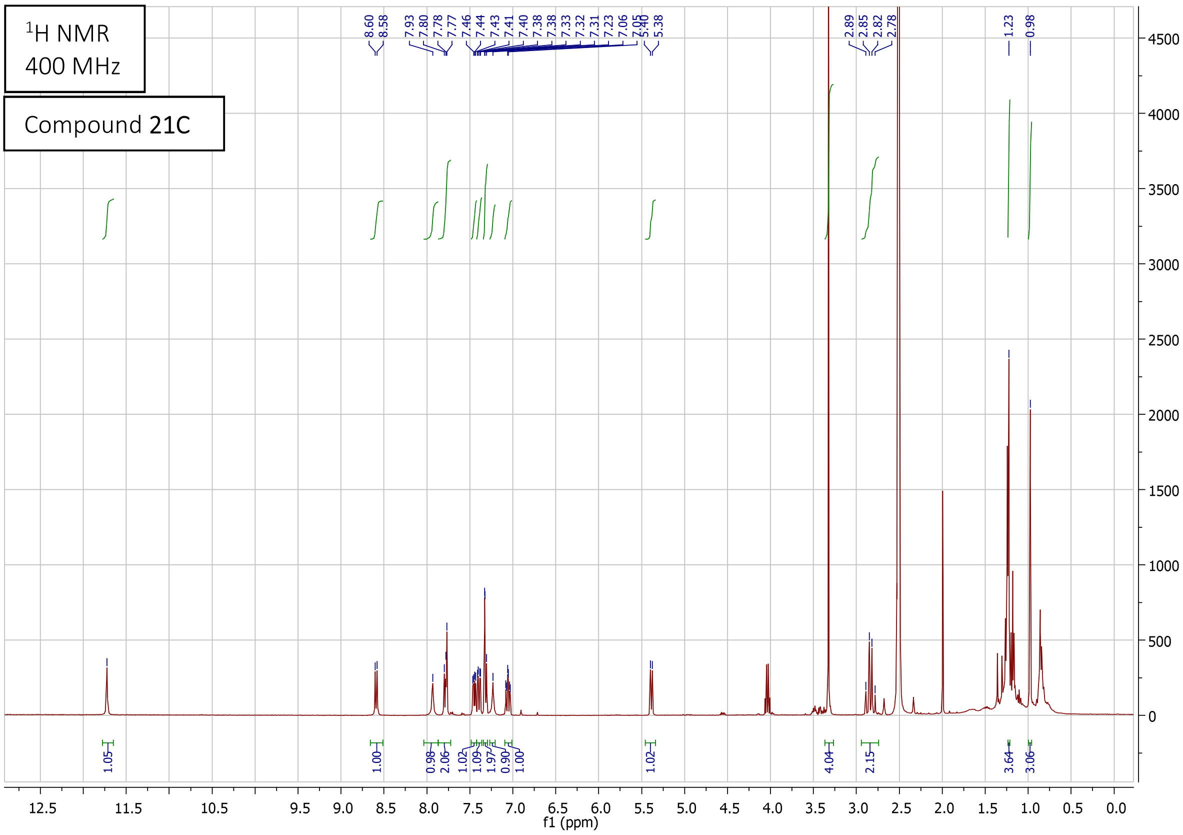

^{1}H NMR
400 MHz
Compound 21C
f1 (ppm)

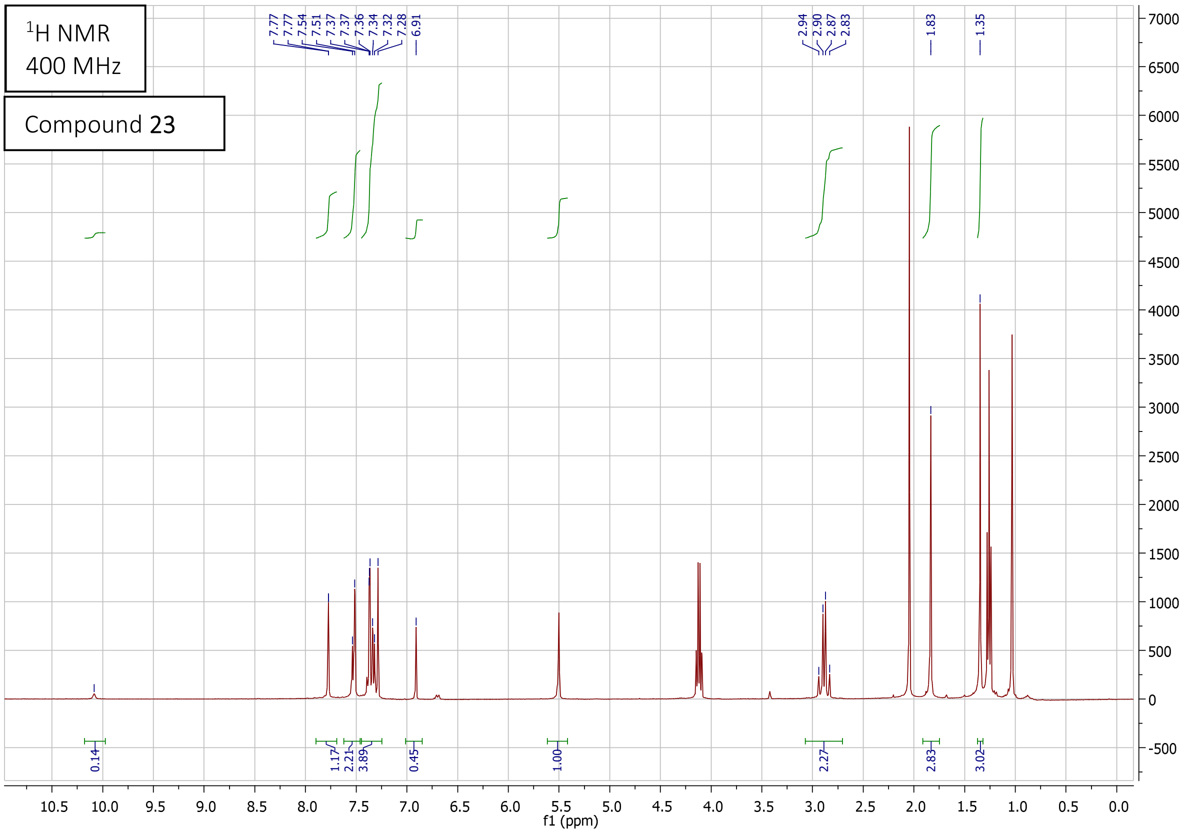

^{1}H NMR
400 MHz
Compound 23
f1 (ppm)

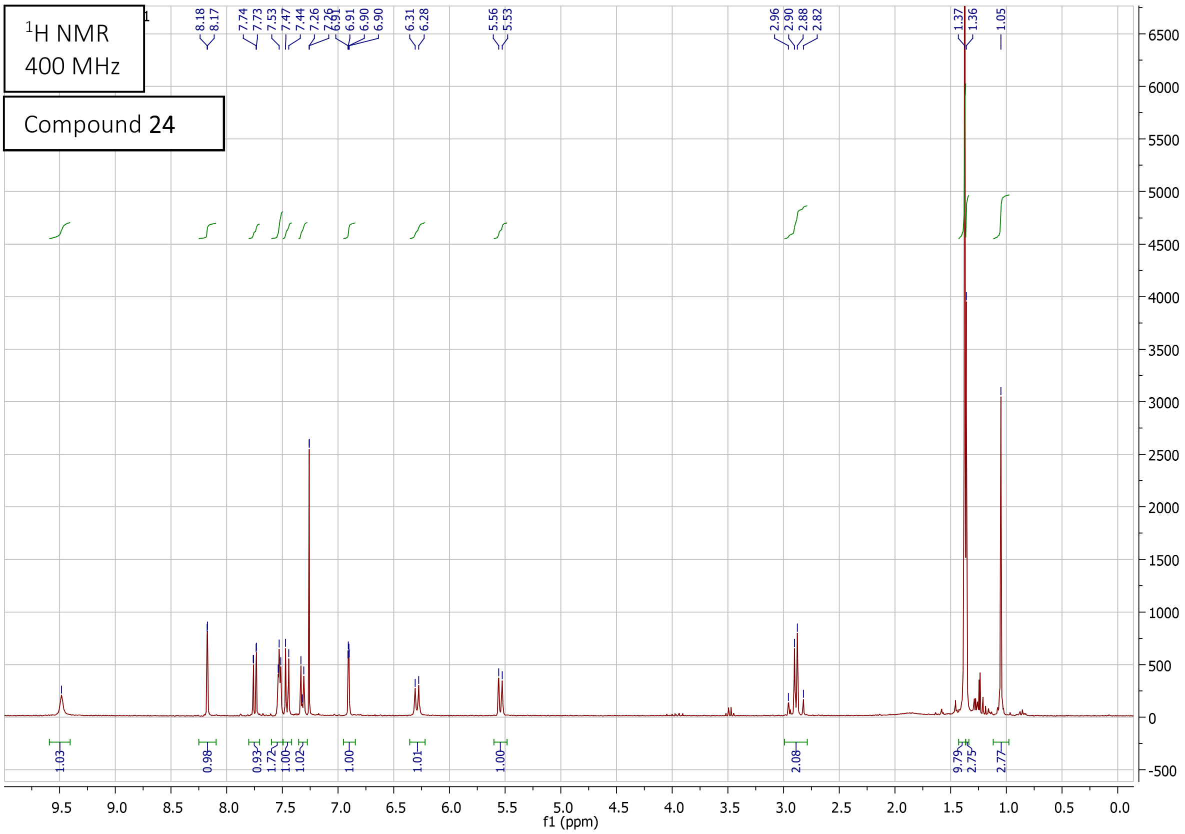

^{1}H NMR
400 MHz
Compound 24
f1 (ppm)

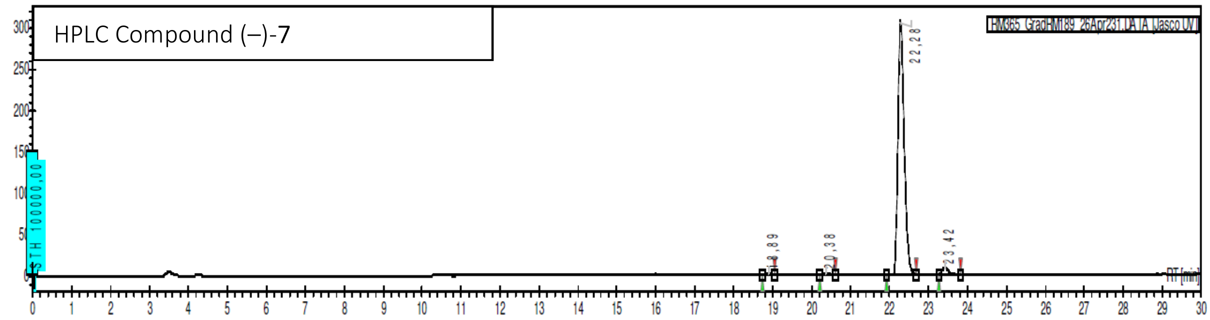

Index	Name	Time [Min]	Quantity [% Area]	Height [mV]	Area [mV.Min]	Area % [%]
3	UNKNOWN	18,89	0,13	0,4	0,1	0,128
2	UNKNOWN	20,38	0,58	1,9	0,4	0,578
4	UNKNOWN	22,28	96,91	308,8	59,2	96,905
1	UNKNOWN	23,42	2,39	7,7	1,5	2,388
Total			100,00	318,8	61,1	100,000

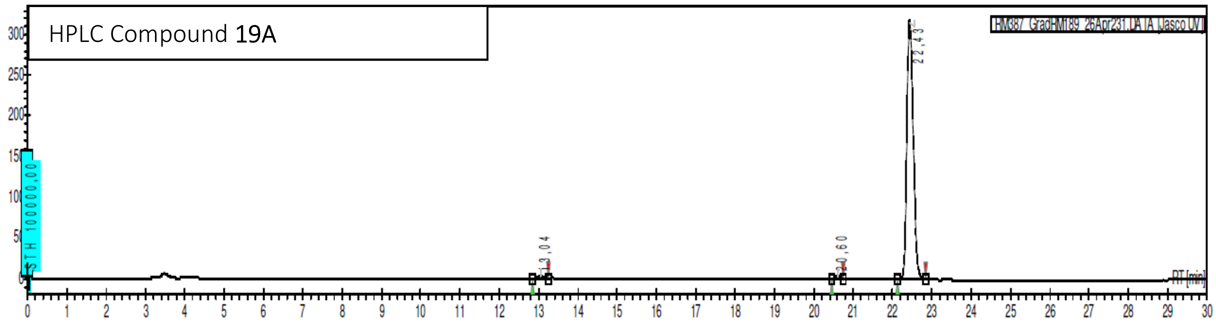

Index	Name	Time [Min]	Quantity [% Area]	Height [mV]	Area [mV.Min]	Area % [%]
3	UNKNOWN	13,04	0,90	3,7	0,5	0,900
2	UNKNOWN	20,60	1,00	3,9	0,6	1,000
1	UNKNOWN	22,43	98,10	318,9	58,4	98,100
Total			100,00	326,6	59,5	100,000

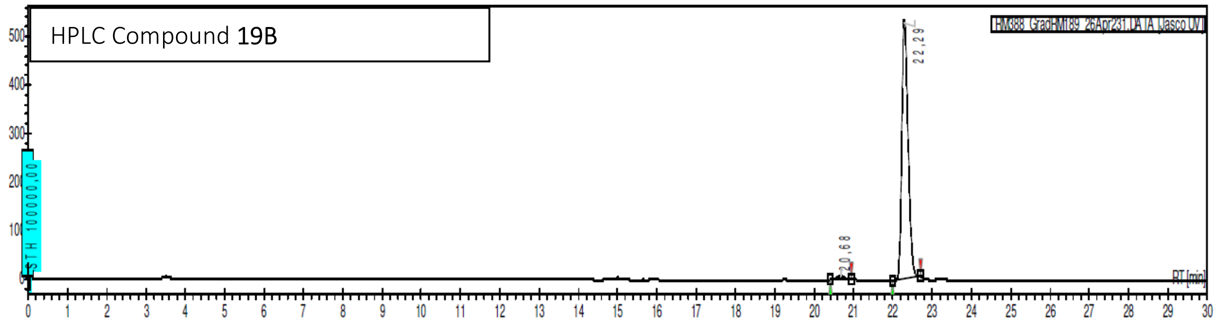

Index	Name	Time [Min]	Quantity [% Area]	Height [mV]	Area [mV.Min]	Area % [%]
2	UNKNOWN	20,68	1,45	7,2	1,5	1,452
1	UNKNOWN	22,29	98,55	532,1	100,2	98,548
Total			100,00	539,3	101,7	100,000

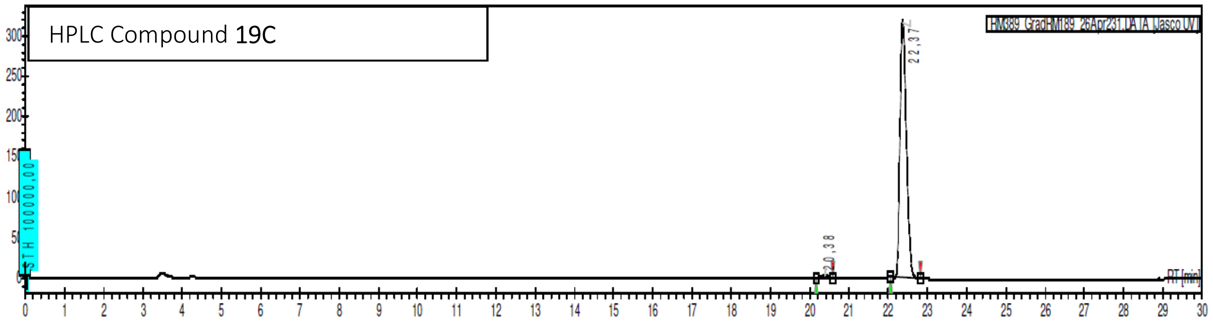

Index	Name	Time [Min]	Quantity [% Area]	Height [mV]	Area [mV.Min]	Area % [%]
2	UNKNOWN	20,38	1,18	3,7	0,7	1,175
1	UNKNOWN	22,37	98,82	320,3	59,0	98,825
Total			100,00	324,0	59,7	100,000

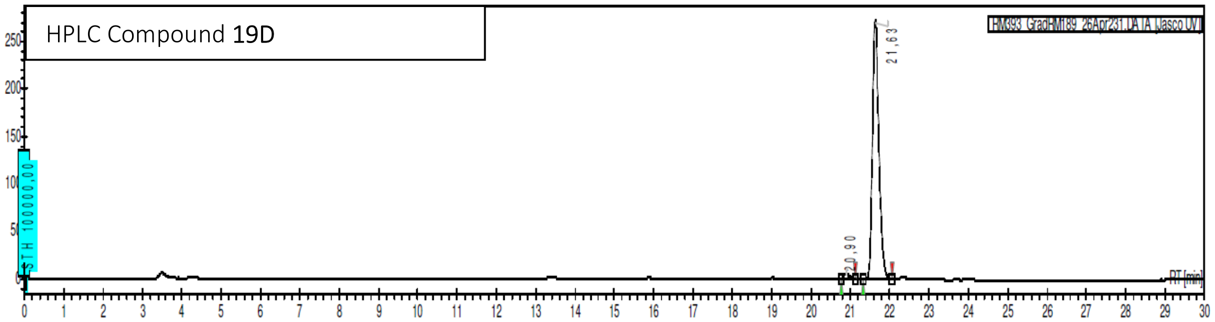

Index	Name	Time [Min]	Quantity [% Area]	Height [mV]	Area [mV.Min]	Area % [%]
2	UNKNOWN	20,90	1,70	5,3	0,9	1,696
1	UNKNOWN	21,63	98,30	273,8	52,6	98,304
Total			100,00	279,2	53,6	100,000

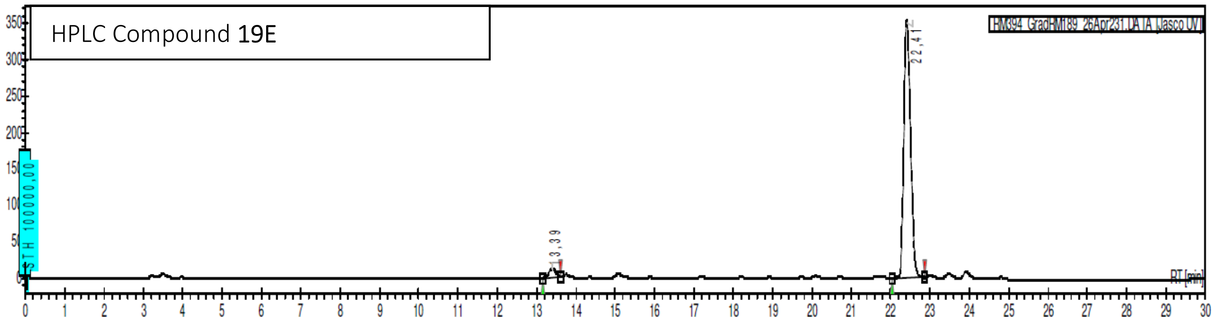

Index	Name	Time [Min]	Quantity [% Area]	Height [mV]	Area [mV.Min]	Area % [%]
2	UNKNOWN	13,39	3,56	13,1	2,5	3,556
1	UNKNOWN	22,41	96,44	353,8	68,6	96,444
Total			100,00	366,9	71,1	100,000

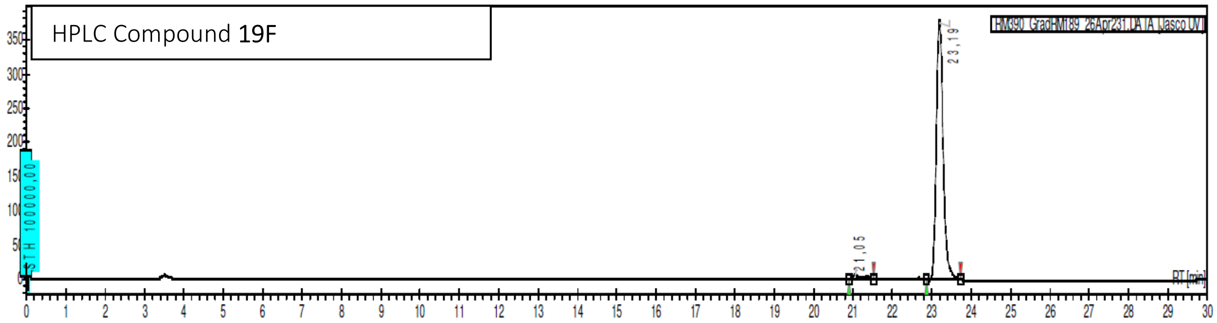

Index	Name	Time [Min]	Quantity [% Area]	Height [mV]	Area [mV.Min]	Area % [%]
2	UNKNOWN	21,05	2,60	7,1	2,1	2,599
1	UNKNOWN	23,19	97,40	380,8	77,0	97,401
Total			100,00	387,9	79,0	100,000

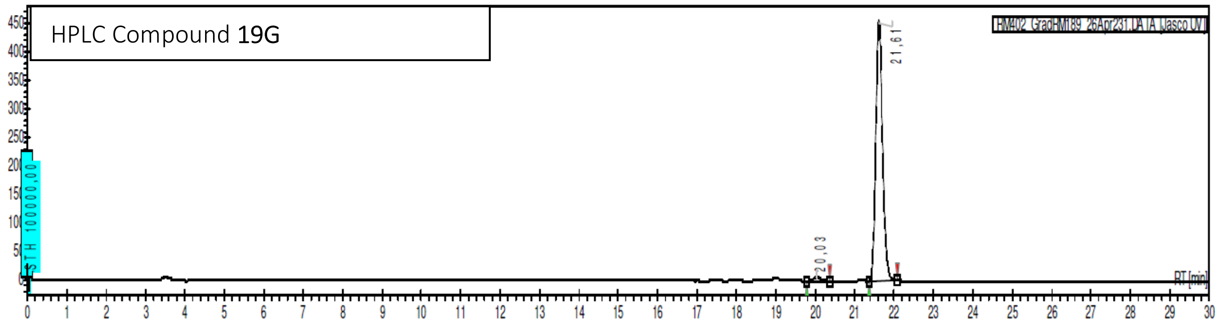

Index	Name	Time [Min]	Quantity [% Area]	Height [mV]	Area [mV.Min]	Area % [%]
2	UNKNOWN	20,03	1,44	6,7	1,3	1,440
1	UNKNOWN	21,61	98,56	457,3	91,6	98,560
Total			100,00	464,0	92,9	100,000

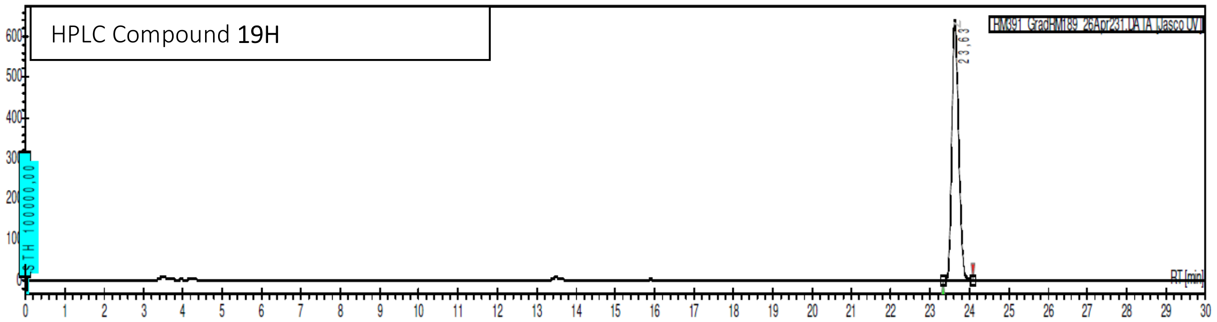

Index	Name	Time [Min]	Quantity [% Area]	Height [mV]	Area [mV.Min]	Area % [%]
1	UNKNOWN	23,63	100,00	644,2	130,5	100,000
Total			100,00	644,2	130,5	100,000

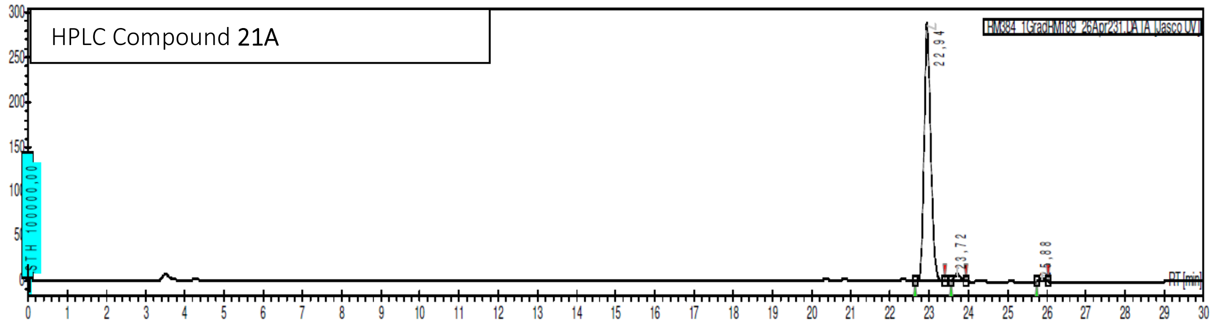

Index	Name	Time [Min]	Quantity [% Area]	Height [mV]	Area [mV.Min]	Area % [%]
1	UNKNOWN	22,94	96,96	290,4	57,9	96,964
2	UNKNOWN	23,72	2,20	7,2	1,3	2,198
3	UNKNOWN	25,88	0,84	3,0	0,5	0,837
Total			100,00	300,6	59,7	100,000

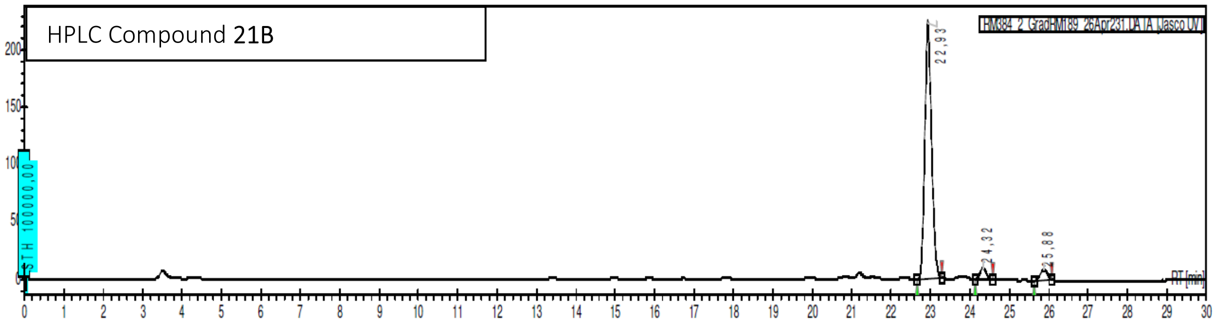

Index	Name	Time [Min]	Quantity [% Area]	Height [mV]	Area [mV.Min]	Area % [%]
1	UNKNOWN	22,93	92,51	226,6	45,5	92,513
2	UNKNOWN	24,32	3,82	10,1	1,9	3,821
3	UNKNOWN	25,88	3,67	8,6	1,8	3,666
Total			100,00	245,3	49,2	100,000

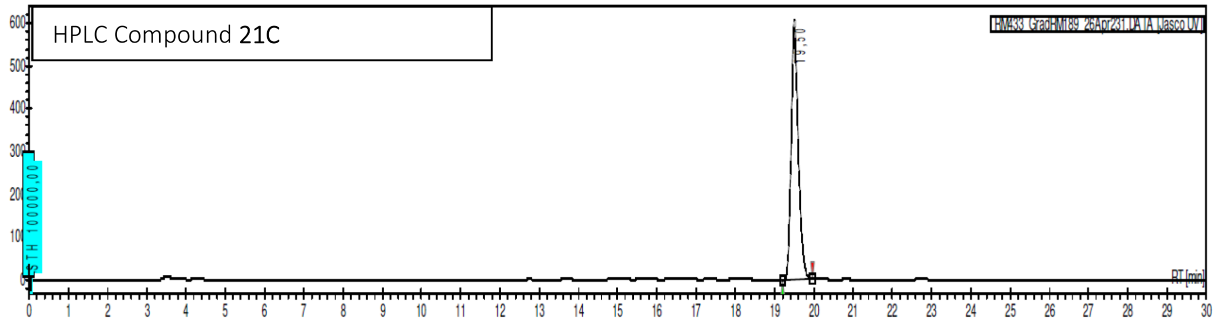

Index	Name	Time [Min]	Quantity [% Area]	Height [mV]	Area [mV.Min]	Area % [%]
1	UNKNOWN	19,50	100,00	609,2	129,3	100,000
Total			100,00	609,2	129,3	100,000

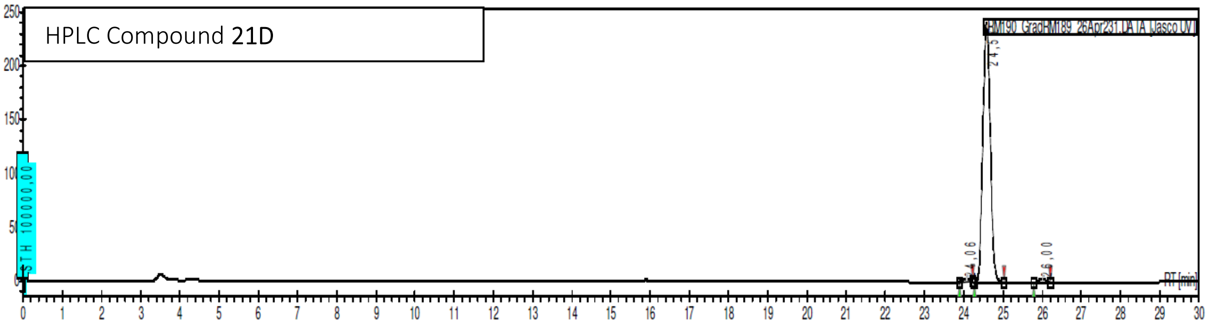

Index	Name	Time [Min]	Quantity [% Area]	Height [mV]	Area [mV.Min]	Area % [%]
2	UNKNOWN	24,06	0,96	3,0	0,5	0,958
1	UNKNOWN	24,58	97,63	243,1	53,0	97,634
3	UNKNOWN	26,00	1,41	3,6	0,8	1,408
Total			100,00	249,7	54,2	100,000

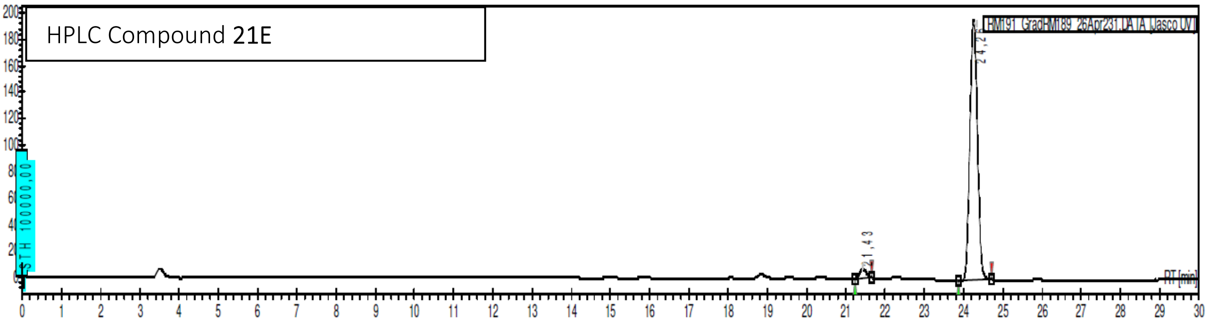

Index	Name	Time [Min]	Quantity [% Area]	Height [mV]	Area [mV.Min]	Area % [%]
2	UNKNOWN	21,43	3,20	7,0	1,4	3,205
1	UNKNOWN	24,26	96,80	196,6	43,5	96,795
Total			100,00	203,6	45,0	100,000

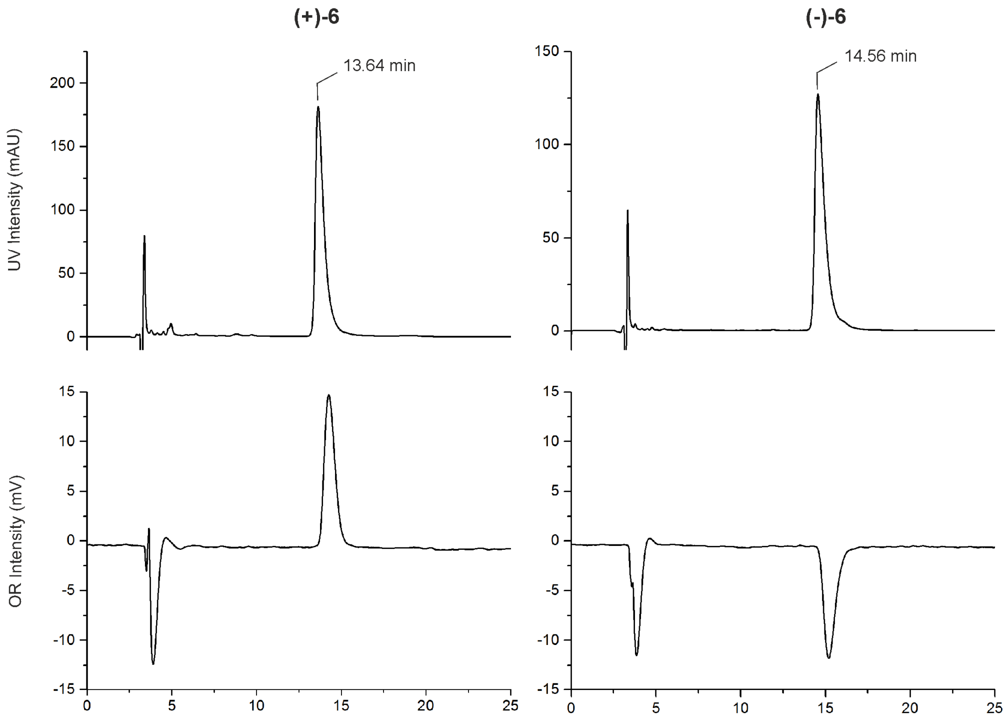

Figure S1. Analytical UV and OR chromatograms of (+)-**6** and (–)-**6**. Conditions: CHIRALPAK IA (250 × 4.6 mm), 58% ACN/ aq. 20mM NH_4OAc, flow 1.0

In vitro autoradiography

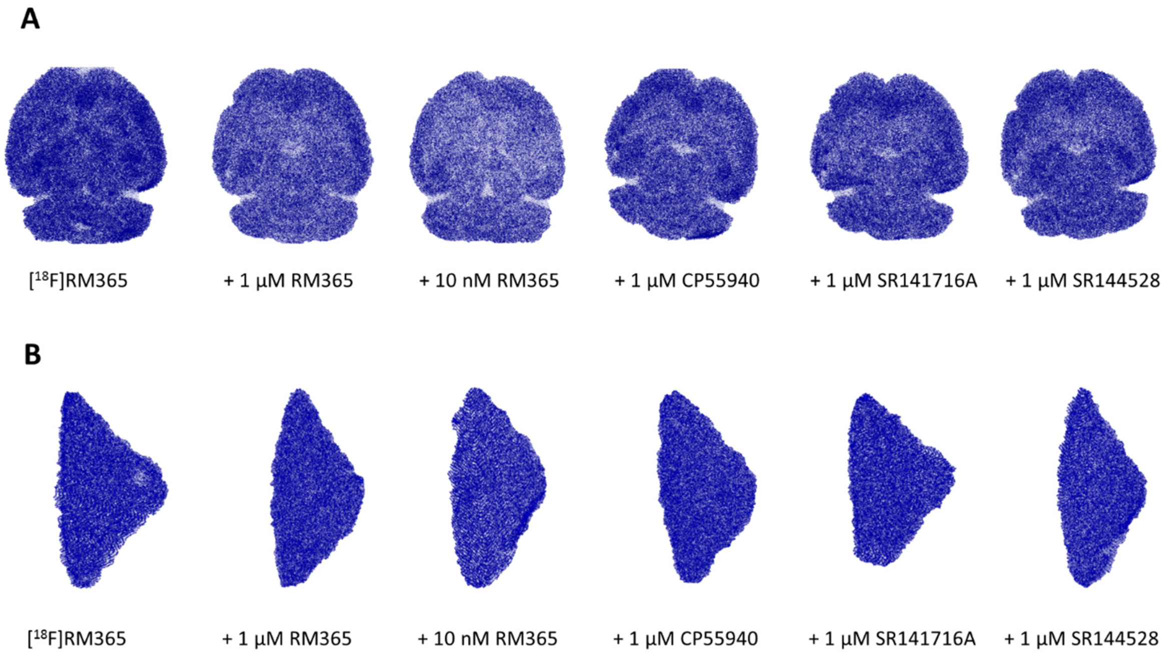

Figure S2. A) Mouse brain in vitro autoradiography of [^{18}F]RM365. Cryosections of a brain of a female CD-1 mouse were incubated with [^{18}F]RM365 alone (total binding) or

in the presence of RM365 at 1 µM or 10 nM (self blocking), or of 1 µM CP55940 (CB1R/CB2R non-selective agonist), SR141716A (CB1R-selective antagonist), or SR144528 (CB2R-selective antagonist). B) Rat spleen in vitro autoradiography of [^{18}F]RM365. Cryosections of spleens of two female SPRD rats were incubated with [^{18}F]RM365 alone (total binding) or in the presence of RM365 at 1 µM or 10 nM (self blocking), or of 1 µM CP55940 (CB1R/CB2R non-selective agonist), SR141716A (CB1R-selective antagonist), or SR144528 (CB2R-selective antagonist).

Computational Chemistry.

1. Geometry optimisation.

The geometry optimisation of R-(–)-**6** and S-(+)-**6** was done using density functional theory (DFT)[1] and performed with the ORCA 5.0 package[2]. The functional B3LYP[3] has been used. In order to improve the stability of the optimised geometry Becke-Johnson dispersion corrections of third order (D3BJ)[4] were included in the functional. We applied the density fitting technique resolution-of-identity approximation (RI-J)[5] and chain-of-sphere approximation (COSX)[5] in the geometry optimisation to speed up the calculations. The optimised geometry was verified based on the frequency calculation, where no imaginary frequencies were observed.

Protein structure preparation

1. Human Cannabinoid Receptor type 2 (hCB2R).

The crystal structure of the hCB2R is available from the protein data bank (PDB) under the code 3ZTY[6] and contains the synthetic ligand AM2051 in the binding pocket of the receptor. The ligand was eliminated from the binding pocket of the receptor and the initial structure of hCB2R was protonated using Reduce program[7].

2. Rattus Cannabinoid Receptor type 2 (rCB2R).

2.1 Folding.

Since the crystallographic structure of rat CB2R is not available we carried out *de novo* modelling. One of the main goals of this modelling is the folding of the primary sequence (mentioned below).

MEECWVTEIANGSKDGLDSNPMKDYMILSGPQKTAVAVLCTLLGLLSALENVAVLYLILS
SHQLRRKPSYLFIGSLAGADFLASVVFACSFVNFHVFHGVDSKAVFLLKIGSVTMTFTAS
VGSLLLTAIDRYLCLRYPPSYKALLTRGRALVTLGIMWVLSALVSYLPLMGWTCCPRPCS
ELFPLIPNDYLLSWLLFIAFLFSGIIYTYGHVLWKAHQHVASLSGHQDRQVPGMARMRLD
VRLAKTLGLVLAVLLICWFPVLALMAHSLATTLSDQVKKAFAFCSMLCLINSMVNPVIYA
LRSGEIRSSAHHCLAHWKKCVRGLGSEAKEEAPRSSVTETEADGKITPWPDSRDLDLSDC

For this purpose we applied I-TASSER[8-9] software. I-TASSER identifies PDB templates with the similar primary sequencies and constructs several models. Five models have been generated based on several PDB templates: 2HPY[10], 2KS9[11], 2ZIY[12], 5TGZ[13], 5UEN[14], 5ZTY[15], 7FEE[16] and 7WC4[17] (Figure S2). These five models differ from each other in their loop conformation and helix orientation. However for the further modelling we considered only the best model **1**. This model have been selected

based on the several scores: C-score, number of decoys and cluster density (Table S1).

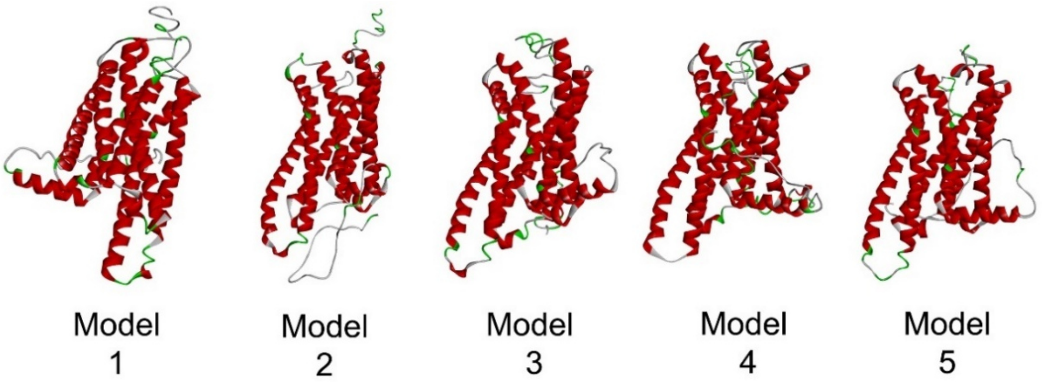

Figure S3. Five models of rat CB2R generated based on template modelling.

Table S1. Parameters estimating the quality of the generated models created via folding of the primary sequence.

**Number of model**	**1**	**2**	**3**	**4**	**5**
**C-score**	-1.51	-1.51	-2.54	-3.96	-2.80
**Number of decoys**	1165	889	481	105	269
**Cluster density**	0.0823	0.0821	0.0295	0.071	0.0226

C-score is a confident score allowing to estimate the quality of the predicted model. The better model has the higher C-score, which typically varies in the range [-5, 2]. Number of decoys (number of the low temperature replicas) are generated according to the algorithm and clustered by SPICKER. The cluster density is determined by the number of decoys at the unit space of the cluster. The higher number of decoys and cluster density indicate the model of higher quality. Thus, the model **1** based on these parameters is the best generated model. For further computations we considered only this model.

2.2 Molecular dynamic

In order to improve the generated model **1** we optimised its structure applying molecular dynamic in the water box. The force field CHARMM[18] was assigned in NAMD[19] for the atoms of the receptor. The molecular dynamic minimisation (Langevin dynamic)[20] has been applied in order to minimise the energy of the structure is the water. The dynamic was run for 500 ps with langevin thermostat at 310K. The integration timestep is 2 fs/step and the size of the water cell is 74 x 68 x 106 Å^3 with the centre placed at 70.10, 71.40, 73.11 of x,y,z-coordinates. The rigid model was applied for the water molecules (TIP3P). For the long-range electrostatic interaction we used the particle-meshed Edwald method[21] (PME).

Docking.

1. Human Cannabinoid receptor type 2.

The molecular docking was performed with the AutoDockTools4 software[22] using the Lamarckian Genetic Algorithm[23]. The water molecules were eliminated and the non-polar hydrogen atoms were merged. The docking area was limited by the constructed grid box of the size 40 x 52 x 48 centred at 8.21, −0.606, -52.343 of x,y,z-coordinates (based on the position of the ligand binding domain (LBD)). The following parameters were used in the docking: number of hybrid GA-LS runs: 500; population size: 150; maximum number of energy evaluations: 25,000,000, maximum number of top individuals to survive to next generation: 1; rate of gene mutation: 0.02; rate of crossover: 0.8; Mean of Cauchy distribution for gene mutation: 0.0; variance of Cauchy distribution for gene mutation: 1.0. The ligand binding domain (LBD) of hCB2R was selected based on the crystallographic data of the receptor.

2. Rat Cannabinoid receptor type 2.

After the energy of the model **1** was minimised, we took optimised coordinates of the receptor in order to carry out the docking. In order to establish the possible binding pockets of rCB2R, we determined the hydrophobic sites of the receptor model and compared them to the binding sites of the known protein templates. Based on this comparison we have selected three possible sites of the receptor (Fig. S3) and compared the binding energies of the best ranked poses of R-(–)**-6** inside of the each site. The molecular docking was performed with the similar algorithm and software as for hCB2R.

The following parameters were used for all three biding positions: number of hybrid GA-LS runs: 500; population size: 150; maximum number of energy evaluations: 25,000,000, maximum number of top individuals to survive to next generation: 1; rate of gene mutation: 0.02; rate of crossover: 0.8; Mean of Cauchy distribution for gene mutation: 0.0; variance of Cauchy distribution for gene mutation: 1.0. The constructed grid box limiting the docking area had the same size for A, B and C: 46, 40, 44 centered at 84.237, 62.779, 83.571 for A, at 72.544, 73.01, 60.599 for B and 61.498, 83.45, 76.266 for C of x,y,z-coordinates.

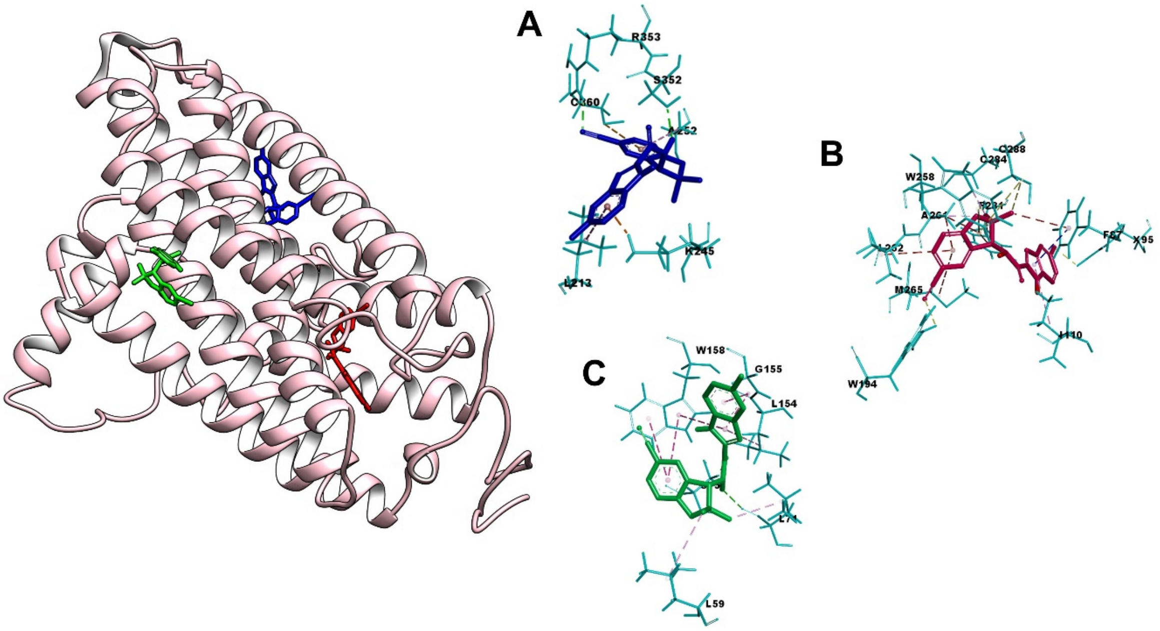

Figure S4. *In silico* investigation of the binding modes of compound **6** based on docking in the three binding sites of rCB2R (left). The highest ranked docked positions of R-(–)-**6** are shown as the pose A (blue) interacting with A252, R353, S352, C360, K245 and L213; the pose B (red) interacting with A261, W258, C284, C288, L252, M265, W194, I110, F87 and X95; the pose C (green) interacting with W158, G155, L154, L59 and L71.

The binding energies of the best three ranked poses made up -6.04, -6.64 and -6.79 kcal mol^{-1} for A, B and C accordingly. According to the binding energy score the most stable poses are B and C. The pose C exhibit slightly higher stability than B; however, these poses are almost equally stable. The pose B is involved in the interactions with the similar amino acid residues in rCB2R as R-(–)-**6** in hCB2R. For further comparison with hCB2R we considered only the pose B in the model of rCB2R.

3. Docking study of S-(+)-**6**.

In order to investigate the binding abilities of S-(+)-**6** in hCB2R and rCB2R we carried out docking of the optimised geometry of S-(+)-**6** in the ligand binding pockets of the receptors. The same docking parameters, algorithm and software were applied as described above for R-(–)-**6**. In spite of docking was not able to reproduce the affinity for R and S isomers precisely[24], it shows the correlation with the experimental results. First of all, the best ranked pose of S-(+)-**6** exhibited the less stable binding with the binding energy -9.71 kcal mol^{-1} in the LBD of hCB2R compare to R-(–)-**6** with the energy -10.45 kcal mol^{-1}. Interesting that the best binding pose of S-isomer is moved closer to the loop of the receptors, where the dominating pi-alkyl interactions have weaker stabilising factor on S-isomer than the pi-pi-T shaped interactions on R-(–)-**6** (Figure S5).

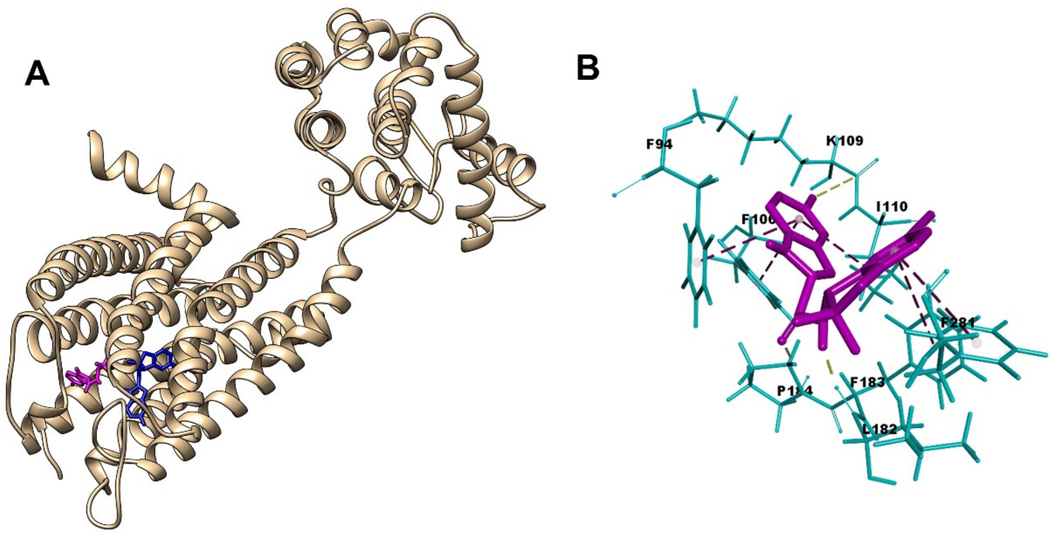

Figure S5. *In silico* investigation of the binding modes of compound **6** based on docking in hCB2R. The highest ranked docked positions of the compounds R-(–)**-6** (blue) and S-(+)**-6** (purple) are shown in the ligand binding domain (**A**). S-(+)**-6** interacts with K109, I110, F94, F281, F183, P184, L182 and F106 (**B**).

The weaker binding was observed also when S-(+)**-6** was docked in rCB2R. The binding energy for the best docked pose of S-isomer made up -6.15 kcal mol^{-1} compare to -6.64 kcal mol^{-1} of R-(–)**-6** (Figure S5).

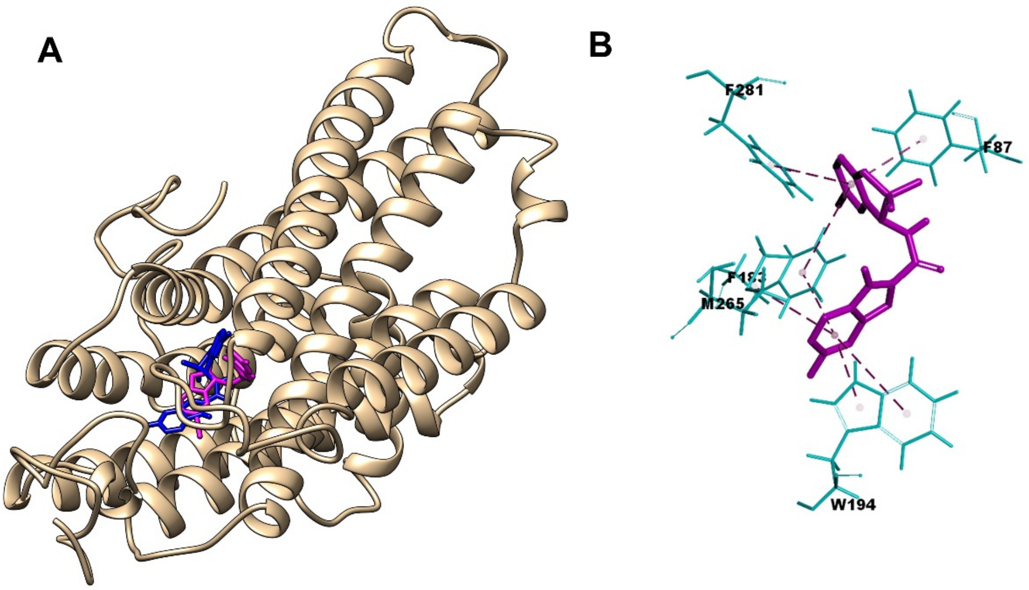

Figure S6. *In silico* investigation of the binding modes of compound **6** based on docking in rCB2R. The highest ranked docked positions of the compound R-(–)**-6** (blue) and S-(+)**-6** (purple) are shown (**A**). S-(+)**-6** interacts with W194, F87, F281, F183 and M265 (**B**).

PET imaging

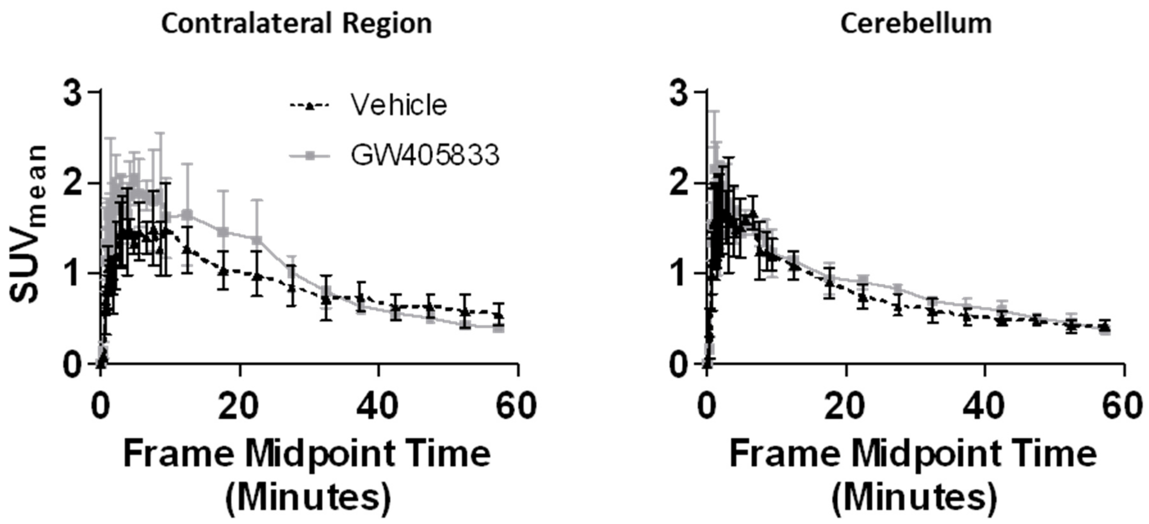

TAC Parameter	Contralateral Vehicle	Contralateral GW405833	Contralateral p-Value	Cerebellum Vehicle	Cerebellum GW405833	Cerebellum p-Value
$AUC_{0\text{-}10}$ (SUV · min)	12 (9 to 15)	14 (2 to 34)	0.077	13 (2 to 28)	14 (2 to 30)	0.293
$AUC_{10\text{-}20}$ (SUV · min)	10 (7 to 12)	11 (2 to 27)	0.176	8 (1 to 18)	9 (1 to 19)	0.368
$AUC_{20\text{-}40}$ (SUV · min)	17 (12 to 23)	20 (3 to 45)	0.205	13 (2 to 29)	16 (2 to 34)	0.109
$AUC_{40\text{-}60}$ (SUV · min)	12 (9 to 16)	10 (1 to 21)	0.138	9 (1 to 20)	10 (1 to 22)	0.251

Figure S7. Time activity curves of the contralateral site and the cerebellum, with and without (Vehicle) GW405833 (5 mg/Kg, n = 3) administered at 20 min post injection of [^{18}F]LU13 and the corresponding AUCs (n = 3; Mean ± SD).

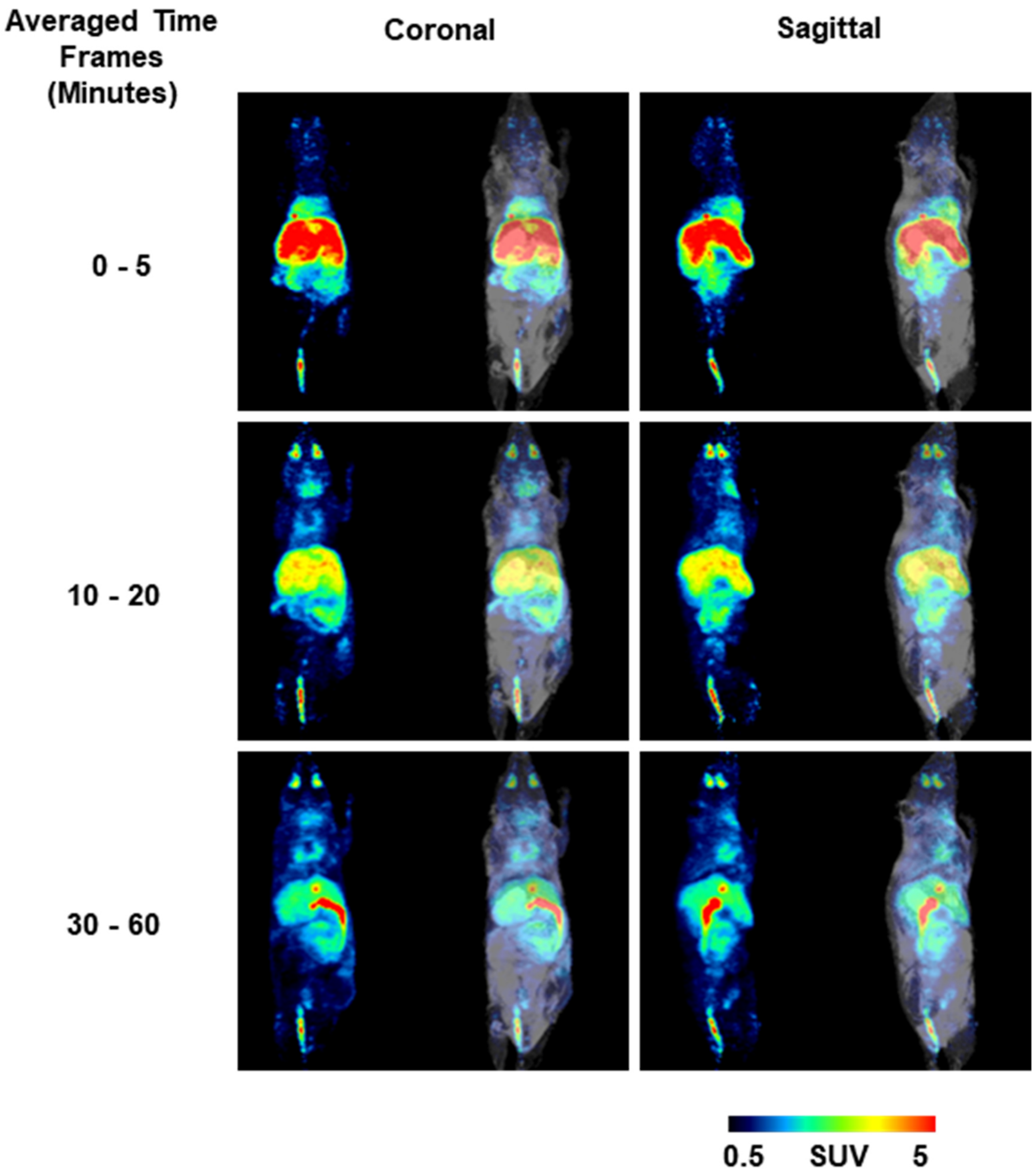

Figure S8. In vivo biodistribution of [^{18}F]RM365 after intravenous administration in an exemplary CD-1 mouse. Shown are maximal intensity projections (MIP) of the time averaged reconstructed PET recordings in coronal and sagittal view. MIPs of PET merged with T1w MR image (right).

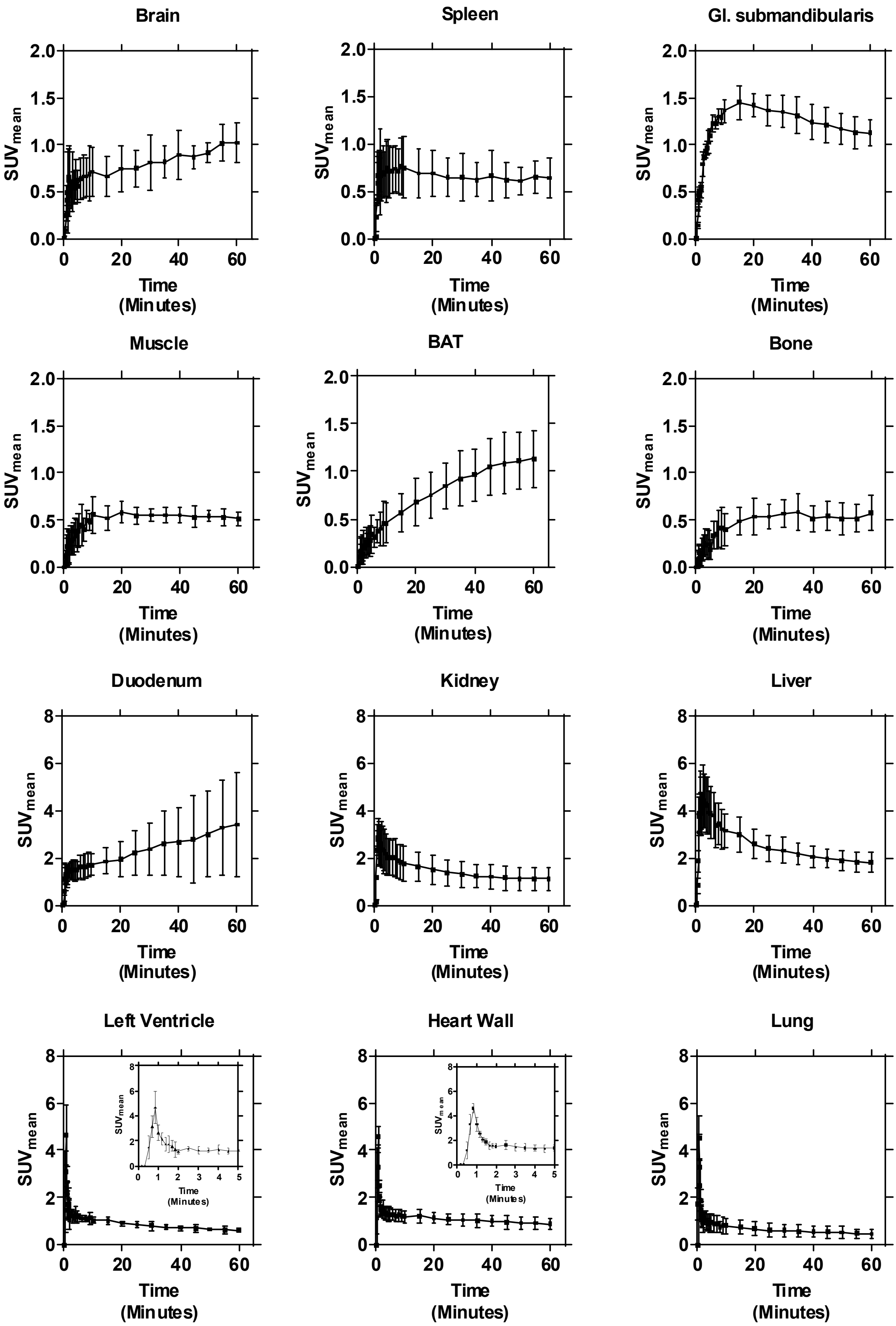

Figure S9. [18F]RM365 uptake over time after intravenous administration in indicated tissues of CD-1 mice represented in time activity curves. N = 4, mean ± SD.

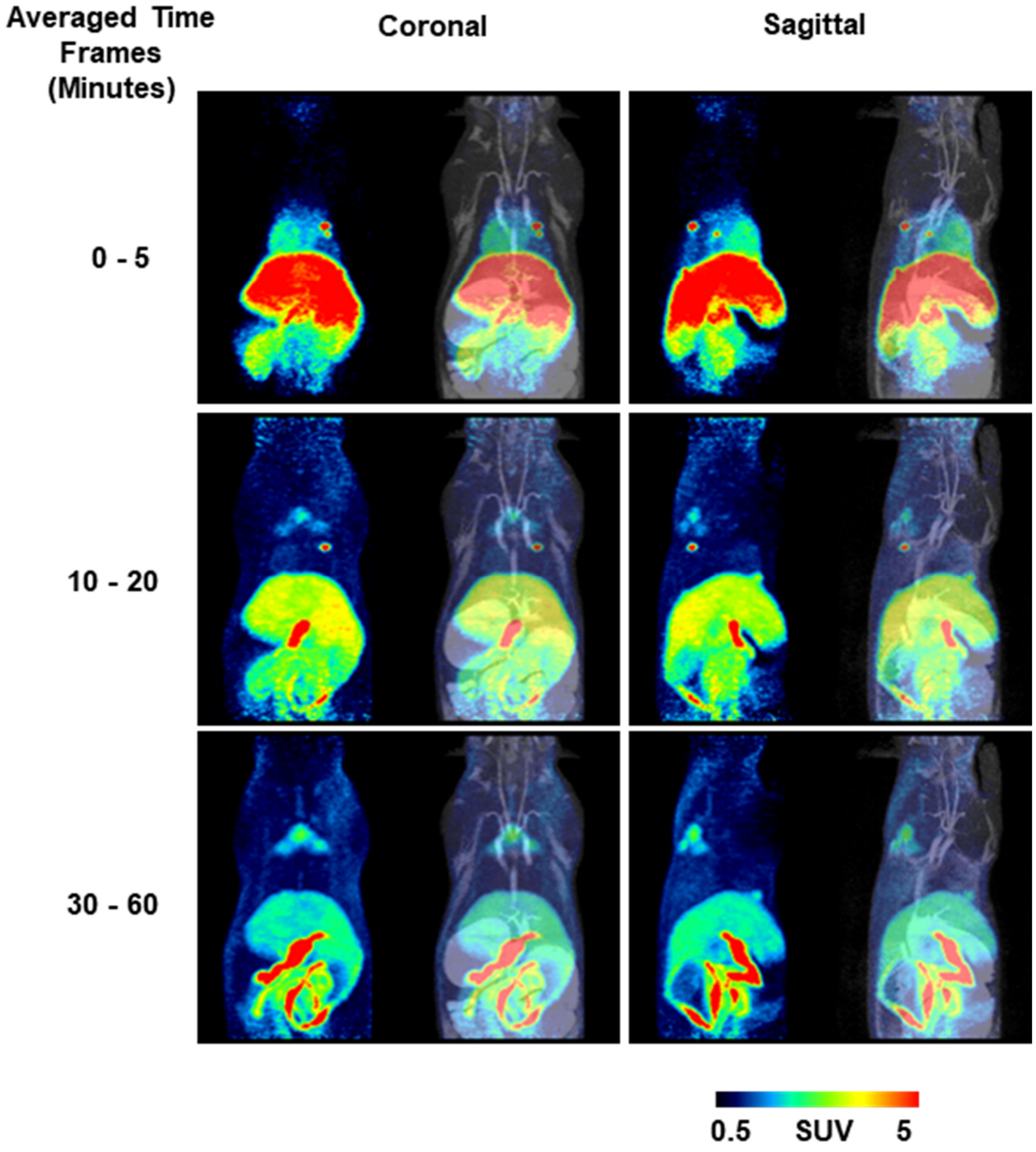

Figure S10. Thoracal and abdominal biodistribution of [^{18}F]RM365 after intravenous administration in an exemplary Wistar rat. Shown are maximal intensity projections (MIP) of the time averaged reconstructed PET recordings in coronal and sagittal view. MIPs of PET merged with T1w MR image (right).

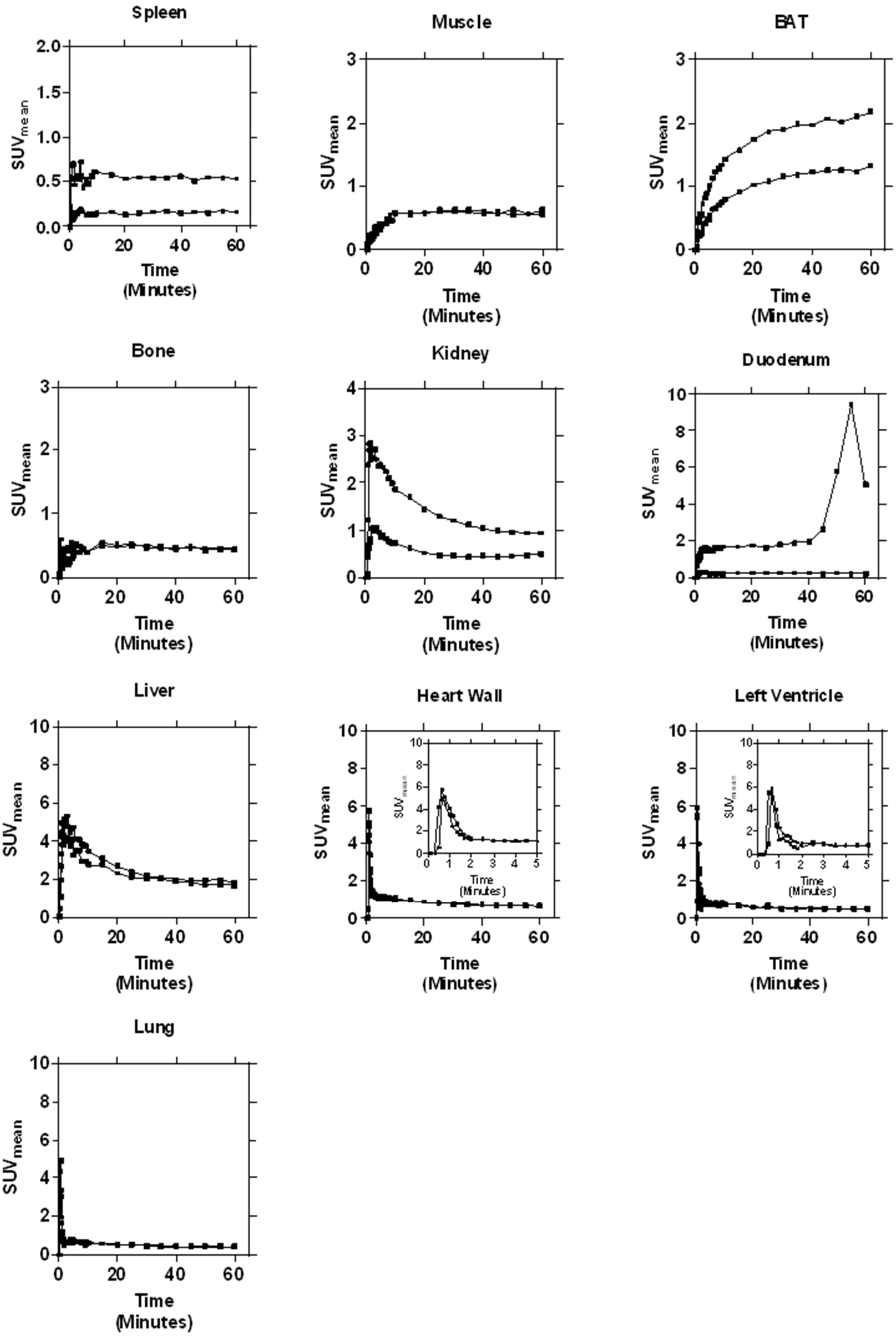

Figure S11. [^{18}F]RM365 uptake over time after intravenous administration in indicated tissues of Wistar rats represented in time activity curves (n = 2).

References

(1) Kohn, W. Density Functional Theory: Basic Results and Some Observations. In *Density Functional Methods In Physics*; Dreizler, R. M., da Providência, J., Eds.; NATO ASI Series; Springer US: Boston, MA, 1985; pp 1–9. https://doi.org/10.1007/978-1-4757-0818-9_1.

(2) Neese, F. The ORCA Program System. *WIREs Computational Molecular Science* **2012**, *2* (1), 73–78. https://doi.org/10.1002/wcms.81.

(3) Coskun, D.; Jerome, S. V.; Friesner, R. A. Evaluation of the Performance of the B3LYP, PBE0, and M06 DFT Functionals, and DBLOC-Corrected Versions, in the Calculation of Redox Potentials and Spin Splittings for Transition Metal Containing Systems. *J. Chem. Theory Comput.* **2016**, *12* (3), 1121–1128. https://doi.org/10.1021/acs.jctc.5b00782.

(4) Schröder, H.; Creon, A.; Schwabe, T. Reformulation of the D3(Becke–Johnson) Dispersion Correction without Resorting to Higher than C6 Dispersion Coefficients. *J. Chem. Theory Comput.* **2015**, *11* (7), 3163–3170. https://doi.org/10.1021/acs.jctc.5b00400.

(5) Kossmann, S.; Neese, F. Efficient Structure Optimization with Second-Order Many-Body Perturbation Theory: The RIJCOSX-MP2 Method. *J Chem Theory Comput* **2010**, *6* (8), 2325–2338. https://doi.org/10.1021/ct100199k.

(6) Gonçalves, S.; Esteves, A. M.; Santos, H.; Borges, N.; Matias, P. M. Three-Dimensional Structure of Mannosyl-3-Phosphoglycerate Phosphatase from Thermus Thermophilus HB27: A New Member of the Haloalcanoic Acid Dehalogenase Superfamily. *Biochemistry* **2011**, *50* (44), 9551–9567. https://doi.org/10.1021/bi201171h.

(7) Word, J. M.; Lovell, S. C.; Richardson, J. S.; Richardson, D. C. Asparagine and Glutamine: Using Hydrogen Atom Contacts in the Choice of Side-Chain Amide Orientation11Edited by J. Thornton. *Journal of Molecular Biology* **1999**, *285* (4), 1735–1747. https://doi.org/10.1006/jmbi.1998.2401.

(8) Zheng, W.; Zhang, C.; Li, Y.; Pearce, R.; Bell, E. W.; Zhang, Y. Folding Non-Homologous Proteins by Coupling Deep-Learning Contact Maps with I-TASSER Assembly Simulations. *Cell Reports Methods* **2021**, *1* (3), 100014. https://doi.org/10.1016/j.crmeth.2021.100014.

(9) Yang, J.; Zhang, Y. I-TASSER Server: New Development for Protein Structure and Function Predictions. *Nucleic Acids Res* **2015**, *43* (W1), W174–W181. https://doi.org/10.1093/nar/gkv342.

(10) Nakamichi, H.; Okada, T. Local Peptide Movement in the Photoreaction Intermediate of Rhodopsin. *Proceedings of the National Academy of Sciences* **2006**, *103* (34), 12729–12734. https://doi.org/10.1073/pnas.0601765103.

(11) Gayen, A.; Goswami, S. K.; Mukhopadhyay, C. NMR Evidence of GM1-Induced Conformational Change of Substance P Using Isotropic Bicelles. *Biochimica et Biophysica Acta (BBA) - Biomembranes* **2011**, *1808* (1), 127–139. https://doi.org/10.1016/j.bbamem.2010.09.023.

(12) *Crystal Structure of Squid Rhodopsin with Intracellularly Extended Cytoplasmic Region - ScienceDirect*. https://www.sciencedirect.com/science/article/pii/S0021925820815221?via%3Dihub (accessed 2023-01-10).

(13) *Crystal Structure of the Human Cannabinoid Receptor CB1 | Elsevier Enhanced Reader*. https://doi.org/10.1016/j.cell.2016.10.004.

(14) Glukhova, A.; Thal, D. M.; Nguyen, A. T.; Vecchio, E. A.; Jörg, M.; Scammells, P. J.; May, L. T.; Sexton, P. M.; Christopoulos, A. Structure of the Adenosine A1 Receptor Reveals the Basis for Subtype Selectivity. *Cell* **2017**, *168* (5), 867-877.e13. https://doi.org/10.1016/j.cell.2017.01.042.

(15) Li, X.; Hua, T.; Vemuri, K.; Ho, J.-H.; Wu, Y.; Wu, L.; Popov, P.; Benchama, O.; Zvonok, N.; Locke, K.; Qu, L.; Han, G. W.; Iyer, M. R.; Cinar, R.; Coffey, N. J.; Wang, J.; Wu, M.; Katritch, V.; Zhao, S.; Kunos, G.; Bohn, L. M.; Makriyannis, A.; Stevens, R. C.; Liu, Z.-J. Crystal Structure of the Human Cannabinoid Receptor CB2. *Cell* **2019**, *176* (3), 459-467.e13. https://doi.org/10.1016/j.cell.2018.12.011.

(16) Arango, D.; Wilson, A. J.; Shi, Q.; Corner, G. A.; Arañes, M. J.; Nicholas, C.; Lesser, M.; Mariadason, J. M.; Augenlicht, L. H. Molecular Mechanisms of Action and Prediction of Response to Oxaliplatin in Colorectal Cancer Cells. *Br J Cancer* **2004**, *91* (11), 1931–1946. https://doi.org/10.1038/sj.bjc.6602215.

(17) Cao, D.; Yu, J.; Wang, H.; Luo, Z.; Liu, X.; He, L.; Qi, J.; Fan, L.; Tang, L.; Chen, Z.; Li, J.; Cheng, J.; Wang, S. Structure-Based Discovery of Nonhallucinogenic Psychedelic Analogs. *Science* **2022**, *375* (6579), 403–411. https://doi.org/10.1126/science.abl8615.

(18) Brooks, B. R.; Brooks III, C. L.; Mackerell Jr., A. D.; Nilsson, L.; Petrella, R. J.; Roux, B.; Won, Y.; Archontis, G.; Bartels, C.; Boresch, S.; Caflisch, A.; Caves, L.; Cui, Q.; Dinner, A. R.; Feig, M.; Fischer, S.; Gao, J.; Hodoscek, M.; Im, W.; Kuczera, K.; Lazaridis, T.; Ma, J.; Ovchinnikov, V.; Paci, E.; Pastor, R. W.; Post, C. B.; Pu, J. Z.; Schaefer, M.; Tidor, B.; Venable, R. M.; Woodcock, H. L.; Wu, X.; Yang, W.; York, D. M.; Karplus, M. CHARMM: The Biomolecular Simulation Program. *Journal of Computational Chemistry* **2009**, *30* (10), 1545–1614. https://doi.org/10.1002/jcc.21287.

(19) Phillips, J. C.; Hardy, D. J.; Maia, J. D. C.; Stone, J. E.; Ribeiro, J. V.; Bernardi, R. C.; Buch, R.; Fiorin, G.; Hénin, J.; Jiang, W.; McGreevy, R.; Melo, M. C. R.; Radak, B. K.; Skeel, R. D.; Singharoy, A.; Wang, Y.; Roux, B.; Aksimentiev, A.; Luthey-Schulten, Z.; Kalé, L. V.; Schulten, K.; Chipot, C.; Tajkhorshid, E. Scalable Molecular Dynamics on CPU and GPU Architectures with NAMD. *J Chem Phys* **2020**, *153* (4), 044130. https://doi.org/10.1063/5.0014475.

(20) Shang, X.; Kröger, M. Time Correlation Functions of Equilibrium and Nonequilibrium Langevin Dynamics: Derivations and Numerics Using Random Numbers. *SIAM Rev.* **2020**, *62* (4), 901–935. https://doi.org/10.1137/19M1255471.

(21) de Souza, O. N.; Ornstein, R. L. Effect of Periodic Box Size on Aqueous Molecular Dynamics Simulation of a DNA Dodecamer with Particle-Mesh Ewald Method. *Biophysical Journal* **1997**, *72* (6), 2395–2397. https://doi.org/10.1016/S0006-3495(97)78884-2.

(22) Morris, G. M.; Huey, R.; Lindstrom, W.; Sanner, M. F.; Belew, R. K.; Goodsell, D. S.; Olson, A. J. AutoDock4 and AutoDockTools4: Automated Docking with Selective Receptor Flexibility. *J Comput Chem* **2009**, *30* (16), 2785–2791. https://doi.org/10.1002/jcc.21256.

(23) Morris, G. M.; Goodsell, D. S.; Halliday, R. S.; Huey, R.; Hart, W. E.; Belew, R. K.; Olson, A. J. Automated Docking Using a Lamarckian Genetic Algorithm and an Empirical Binding Free Energy Function. *J. Comput. Chem.* **1998**, *19* (14), 1639–1662. https://doi.org/10.1002/(SICI)1096-987X(19981115)19:14<1639::AID-JCC10>3.0.CO;2-B.

(24) Phyo, Y. Z.; Cravo, S.; Palmeira, A.; Tiritan, M. E.; Kijjoa, A.; Pinto, M. M. M.; Fernandes, C. Enantiomeric Resolution and Docking Studies of Chiral Xanthonic Derivatives on Chirobiotic Columns. *Molecules* **2018**, *23* (1), 142. https://doi.org/10.3390/molecules23010142.
